# Integrative Taxonomy and Species Delimitation in Harvestmen: A Revision of the Western North American Genus *Sclerobunus* (Opiliones: Laniatores: Travunioidea)

**DOI:** 10.1371/journal.pone.0104982

**Published:** 2014-08-21

**Authors:** Shahan Derkarabetian, Marshal Hedin

**Affiliations:** 1 Department of Biology, San Diego State University, San Diego, California, United States of America; 2 Department of Biology, University of California Riverside, Riverside, California, United States of America; University of Innsbruck, Austria

## Abstract

Alpha taxonomy, and specifically the delimitation of species, is becoming increasingly objective and integrative. The use of coalescent-based methods applied to genetic data is providing new tools for the discovery and delimitation of species. Here, we use an integrative approach via a combination of discovery-based multivariate morphological analyses to detect potential new species. These potential species are then used as *a priori* species in hypothesis-driven validation analyses with genetic data. This research focuses on the harvestmen genus *Sclerobunus* found throughout the mountainous regions of western North America. Based on our analyses, we conduct a revision of *Sclerobunus* resulting in synonymy of *Cyptobunus* with *Sclerobunus* including transfer of *S. cavicolens* **comb. nov.** and elevation of both subspecies of *S. ungulatus*: *S. ungulatus* **comb. nov.** and *S. madhousensis* **comb. nov., stat. nov.** The three subspecies of *S. robustus* are elevated, *S. robustus*, *S. glorietus* **stat. nov.**, and *S. idahoensis* **stat. nov.** Additionally, five new species of *Sclerobunus* are described from New Mexico and Colorado, including *S. jemez* **sp. nov.**, *S. klomax* **sp. nov.**, *S. skywalkeri* **sp. nov.**, *S. speoventus* **sp. nov.**, and *S. steinmanni* **sp. nov.** Several of the newly described species are single-cave endemics, and our findings suggest that further exploration of western North American cave habitats will likely yield additional new species.

## Introduction

In order to accurately identify and describe the diversity of life, species must be delimited operationally [1]. With increasing stability of the conceptual definition of a species [2], the operational delimitation of species becomes an easier task [3], [4]. It is becoming increasingly clear that taxonomy, and species delimitation in particular, must become an integrative field utilizing multiple independent lines of evidence [3], [5] as any single dataset may not accurately reflect species limits and relationships (e.g., due to morphological convergence, gene tree/species tree discordance, etc.). Proper integrative species delimitation should incorporate a combination of discovery-based approaches without *a priori* species hypotheses and, following this, hypothesis-driven approaches, which test those groups that are delimited based on discovery methods [3], [4], [6]. Additionally, in order to decrease the degree of subjectivity that is common in traditional alpha-taxonomic practices, species delimitation methods are moving towards increasing objectivity [4]. For example, recently developed methods accomplish this by relying on statistical thresholds to delimit species in multivariate morphological space [7], or posterior probability distributions to determine support for the presence of a species tree node [8].

The discovery and documentation of biodiversity at the species level is especially important for conservation biology [9], particularly in taxa that are relatively poorly studied (either due to sheer diversity of species and/or proportionally few taxonomists). With the almost ubiquitous incorporation of genetic data in systematics, the discovery and documentation of new and cryptic species is increasing [10], seemingly correlated with the use of coalescent-based species delimitation methods [4], [11]. Recent studies have shown the potential for these methods in the discovery of new and cryptic species in taxa that are either poorly known, morphologically conserved, show low vagility, and/or have narrow ecological limits [12]–[16]. The order Opiliones (commonly called harvestmen) is a diverse group of arachnids with over 6500 described species [17], distributed on every continent except Antarctica. Despite being a fairly diverse group (i.e., more described species than mammals), harvestmen are relatively poorly studied. The vast majority of harvestmen species show low vagility with high ecological constraints; attributes that have resulted in the discovery of many new, and sometimes cryptic, species when studied using modern molecular approaches [18]–[24].

In this study we focus on the harvestmen genera *Sclerobunus* and *Cyptobunus*, which are broadly distributed throughout the mountainous regions of western North America. These genera are ecologically limited to moist, dark microhabitats, typically found under logs and rocks in high-elevation forests or in caves. The first species of *Sclerobunus* was described from Colorado by A. S. Packard in 1877 as *Scotolemon robustum* [25]. Later, Banks [26] described the new genus *Sclerobunus*, and included two species: Packards' *Scotolemon robustum*, renamed *Sclerobunus robustus* (Packard, 1877), and *Sclerobunus brunneus*. This second species was later transferred to the genus *Paranonychus* Briggs, 1971. Banks' [26] description of *Sclerobunus robustus* was based on specimens from Colorado, Utah (originally described by Packard), and Washington. Roewer [27] then described a new species, *Sclerobunus parvus*, from Vancouver Island, British Columbia. Many years later in a revision of the North American “Triaenonychidae”, Briggs [28] included two species of *Sclerobunus*. The first species, *Sclerobunus nondimorphicus* was described as new from Oregon, Washington, and southern British Columbia (this species includes Banks' [26] specimen of *S. robustus* from Washington). The second species, *S. robustus*, was redescribed and recorded with a very broad distribution throughout the American southwest and Rocky Mountains. Within this species, Briggs described three subspecies: *S. robustus robustus* from Arizona and New Mexico, *S. r. glorietus* from a single locality in northern New Mexico, and *S. r. idahoensis* from Idaho. More recently, Shear and Derkarabetian [29], upon examination of the type specimens of *S. parvus*, synonymized this taxon with *Paranonychus brunneus* (Banks, 1893). Currently, *Sclerobunus* includes two species: *Sclerobunus nondimorphicus* and *Sclerobunus robustus*, itself including three subspecies.

The taxonomic and phylogenetic relationship between *Sclerobunus* and its sister genus *Cyptobunus* remains uncertain. All *Cyptobunus* are known only from cave habitats and are highly troglomorphic. Banks [30] described the new genus and species, *Cyptobunus cavicolus*, from Lewis and Clark Caverns, Montana based on a juvenile specimen (as mentioned by Roewer, [31]) and remarked that this taxon “is but a cavernicolous adaptation of *Sclerobunus*” [30]. With adult specimens, Crosby and Bishop [32] synonymized *Cyptobunus* with *Sclerobunus* and this synonymy was acknowledged by Goodnight and Goodnight [33]. Later, Briggs [28] described the new species *C. ungulatus*, with two subspecies: *C. u. ungulatus* from Model Cave in Great Basin National Park, Nevada and *C. u. madhousensis* from North Madhouse Cave near Provo, Utah. Briggs retained separate genera noting differences in several somatic characters, but also remarked on the general similarity in male genitalia. Historically, *Sclerobunus* are only known from epigean habitats. However, recent fieldwork has uncovered many new populations of troglomorphic *Sclerobunus* from cave and talus habitats, extending the known habitat preference for this taxon [21]. *Sclerobunus* populations found in caves show varying degrees of troglomorphy including decreased pigmentation, elongation of appendages, and attenuation of spines. Based on this, it is possible that *Cyptobunus* taxa merely represent highly derived *Sclerobunus*. A recent genetic analysis by Derkarabetian et al. [21] recovered *Cyptobunus* phylogenetically nested within *Sclerobunus* and showed that troglomorphy evolved independently at least three times; however, this was only a two-gene study. Although there remains uncertainty in the relationship between *Sclerobunus* and *Cyptobunus*, their monophyly relative to other closely related genera is certain and reflected in penis morphology [28] and genetic data [21].

The morphological and genetic analyses of Derkarabetian et al. [21] suggested that there are several new morphologically distinct, genetically divergent species within *Sclerobunus*. Here, we use discovery-based methods to identify multivariate clusters using morphometric data, which are then treated as putative species in species tree analyses using eight newly developed nuclear loci. Following this, we use a hypothesis-driven approach to test alternative species delimitation hypotheses using the multigenic nuclear dataset. Based on results of our species delimitation analyses, we conduct a revision of *Sclerobunus*, including *Cyptobunus*.

## Materials and Methods

### Taxon Sampling

Fieldwork was conducted in the summer months of 2006, 2007, and 2008 throughout the mountainous regions of western North America. Samples representing all species and subspecies of *Sclerobunus* and *Cyptobunus* were collected for analyses (TABLE S1). Fresh specimens were collected from type localities for all species and subspecies, except *S. nondimorphicus*, *S. r. robustus*, and *S. r. idahoensis*. For these taxa, type localities could not be accessed either due to vague locality records or unsuitable habitat, and fresh samples were collected from as near the type locality as possible. All specimens were collected by hand; those destined for molecular analyses were placed in 100% EtOH and stored at −80°C, while specimens to be used in morphological analyses were stored in 80% EtOH.

Samples from caves in Great Basin National Park, Nevada were collected under a Scientific Research Collecting Permit from the U.S. National Park Service permit granted to the authors (GRBA-2007-SCI-0008). Permission to collect in Lewis and Clark Caverns, Montana was given by the Montana State Park staff and samples from Cave of the Winds, Colorado were collected with permission from the management staff. In general, sample sizes from cave populations were limited either purposefully due to conservation concerns, or due to rarity of individuals. To increase the sample size for some cave populations, recently collected specimens from Cave of the Winds and newly discovered Mallory Cave, Colorado specimens from the Denver Museum of Nature and Science were included. In addition, specimens collected from Terrero Cave, New Mexico held in the California Academy of Sciences collection were included in morphological analyses.

### Morphometric Analyses

The morphometric dataset included 18 linear measurements taken using an Olympus SZX12 microscope equipped with an ocular micrometer (TABLE S2). The characters chosen included the length and width of the scute, chelicerae, and genital operculum, height and width of the ocularium, height of the pedipalpal femur, and length of the second leg. For some specimens, certain characters could not be measured, for example, missing genital opercula or legs (total of 2.4% missing data). In these cases, missing values were estimated with SPSS v. 20 (IBM Corp.) using the Multiple Imputation function with default settings and 10 iterations. The average of 10 iterations was used as the missing value in the final analyses. Because of sexual dimorphism, analyses were conducted on separate male and female datasets.

We utilize an algorithmic approach described by Ezard et al. [7] that relies on statistical thresholds at multiple steps to objectively define species clusters in multivariate morphospace. This method is used as a discovery-based approach to identify putative species, which we then further tested with genetic data. The Ezard et al. method has several advantages over traditional PCA [7]. First, traditional PCA using a covariance matrix assume normally distributed data, which is usually not met in biological datasets that contain multiple species. Our data do not meet this assumption as multiple species are included and 7 of 18 characters are not normally distributed (Shapiro-Wilks test; results not shown). Second, analyses computed via correlation matrices can be negatively influenced by outliers, which can skew orientation of the axes. To circumvent these issues, this method uses robust scale estimators that perform better with non-normal data. Use of the median to scale the data de-emphasizes the effects of any outliers, and is more useful in discriminating incipient species. The Bayesian Information Criteria (BIC) is used to choose the optimal model among possible models that vary in the size, orientation, and number of clusters. Importantly, this method can be used as a null model of morphological homogeneity. Finally, the automation and use of multiple thresholds increases reproducibility and similarity in interpretations across datasets and taxa. We implemented the Ezard et al. method using the default protocol with the k-value set to 18 (total number of characters measured in this study) using R 3.0.2 (R Core Team, 2013). In addition, standard principal components (PCA) and discriminant function (DFA) analyses were conducted (see FILE S1 for details); however, we do not use these results in our species delimitation decisions. The PCA and DFA analyses and results are included only as a simple results comparison of the recently developed method of Ezard et al. [7].

We did not include holotype specimens in morphometric analyses for two main reasons: First, all species of *Sclerobunus* are allopatric in distribution, with one exception at Taos Ski Valley, New Mexico, which is discussed below and at length in Derkarabetian et al. [21]. As such, we argue that inclusion of fresh specimens from the type locality is just as valuable as including holotypes. The allopatric distribution of *Sclerobunus* species eliminates the possibility that type series specimens are heterospecific or that specimens collected from type localities represent multiple species. Second, holotype specimens range from 40–100+ years old and may not be in suitable condition for morphometric analyses, particularly the more fragile cave species.

### Molecular Data Collection

Based on a comparison of transcriptomic data (see FILE S2), we developed primers for 61 exon and untranslated (UTR) nuclear gene regions, 23 of which successfully amplified for a small panel of 4 *Sclerobunus* taxa (2 *S. robustus*, 1 *S. glorietus*, and 1 *S. nondimorphicus*). Of these, 8 gene regions were pursued further based on informativeness and minimal levels of heterozygosity. It has been shown that species limits can be successfully determined with ≥5 loci and moderate sample sizes (5 specimens per species), even with short node depths and gene flow [34], [35]. The 8 nuclear loci included six exonic regions and two 3′UTR regions, and were sequenced for 43 total samples of *Sclerobunus* and *Cyptobunus* (TABLE S3) using the primers and PCR conditions detailed in FILE S2. Bi-directional Sanger reads were assembled into contigs, edited, and unambiguously aligned using Geneious Pro 6 (http://www.geneious.com). Diversity statistics and tests for recombination and neutrality for each locus were calculated using DnaSP v 5 [36]. Haplotypes were determined using Phase 2.1.1 [37]. Both of the 3′ UTR loci contained some sequences that could not be resolved to haplotypes. For these two loci, unresolved sequences were removed prior to tests of recombination and neutrality. GenBank accession numbers are provided in TABLE S3. Matrices with phased sequences are available from the Dryad Digital Repository: doi:10.5061/dryad.hj6r1.

### Species Tree Analyses

**Figure 1:**
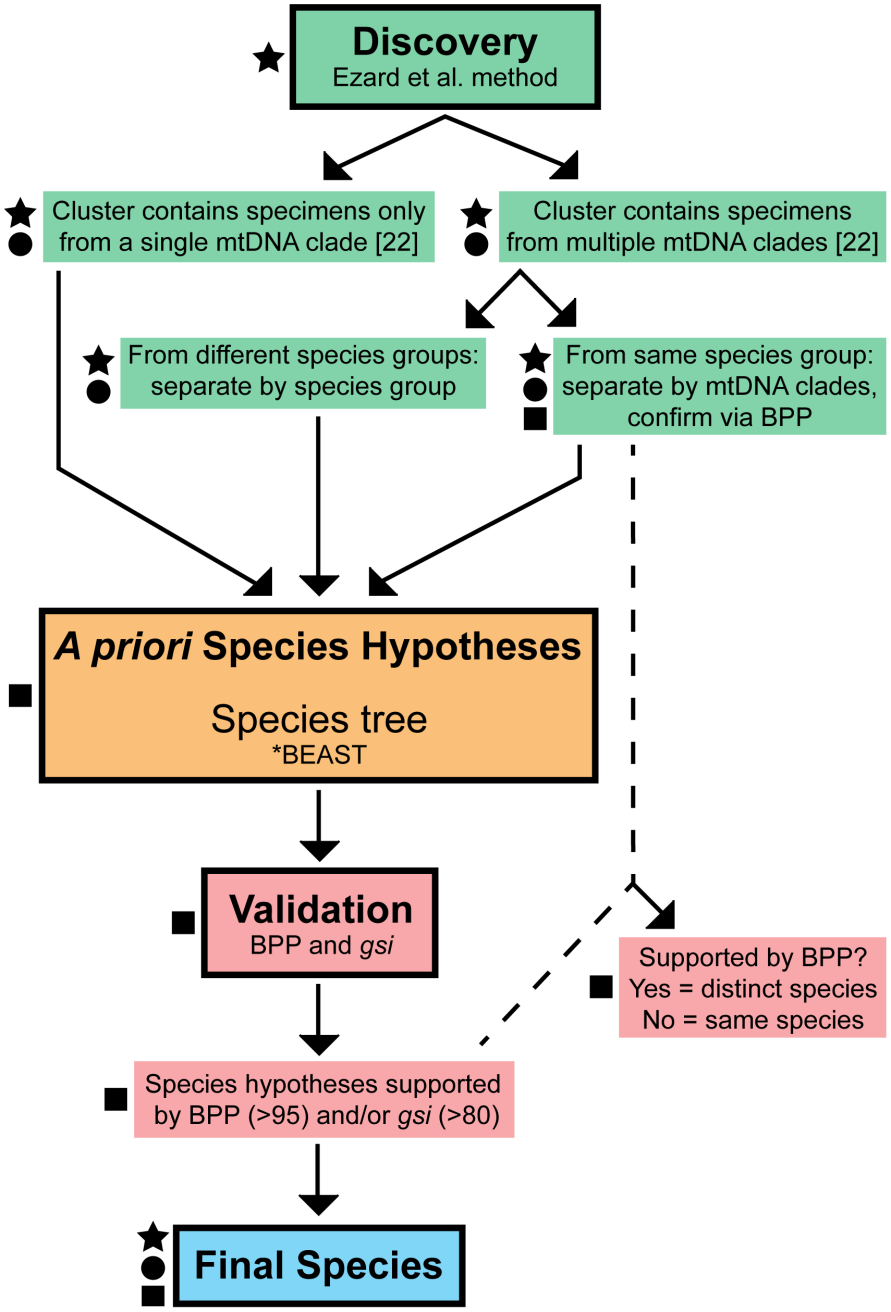
Species delimitation decision-making workflow. Symbols to the left of each step represent the different classes of data used at that particular step: star = morphological data; circle = mitochondrial data (COI); square = nuclear data.

The clusters recovered from morphological, discovery-based analyses were used in conjunction with clades recovered in the COI mtDNA analyses of Derkarabetian et al. [22] to define *a priori* species hypotheses for species trees analyses. In previous analyses, three well-supported, deeply divergent species groups are recovered: a clade from the Pacific Northwest including *S. nondimorphicus* and *S. r. idahoensis*, a clade including all *Cyptobunus*, and a clade including all southwestern *Sclerobunus*. The monophyly and distinctiveness of these three species groups is also reflected in genitalic morphology [21], [28]. Morphometric clusters may include multiple species; in these clusters, species group assignments can help resolve cases of clustering due to broad “morphospace convergence” between taxa from different species groups, or cases of potential conspecificity of putatively different species within the same species groups (FIGURE 1). In instances where clusters contain specimens from two different species groups, those divergent taxa will be considered separate putative species. Clusters with potentially different taxa from the same species group will rely on validation analyses.

All genetic analyses conducted use only the 8 nuclear genes sequenced here; we do not reanalyze or include previously collected nuclear [21] or mitochondrial data [22] (see Discussion). Different populations of the geographically widespread subspecies *S. robustus robustus* represent potential cryptic species [22], but because our current nuclear genetic sampling for this taxon is limited, this subspecies was treated as a single taxon in this paper.

To determine rooting and ingroup polarity, we conducted preliminary analyses including only the six exons with *Theromaster* as the outgroup. The 3′ UTR gene regions were either not present in the sequenced *Theromaster* transcriptome, or could not be unambiguously aligned to the ingroup. Following this, all subsequent species tree analyses were conducted on the complete 8-gene set with *Theromaster* sequences removed. Models of evolution and optimal partitioning strategy were determined with PartitionFinder [38] using the BIC to choose optimal models. The species tree was reconstructed using the *BEAST algorithm [39]. Analyses were run for 200 million generations logging every 1000 generations, implemented in BEAST 1.8 [40] using the CIPRES portal (http://www.phylo.org/sub_sections/portal/). Each resulting run was checked for stationarity and for ESS values above 200 with Tracer 1.5 [41]. Species trees were reconstructed with TreeAnnotator (http://beast.bio.ed.ac.uk/TreeAnnotator) from 180,000 trees (10% burnin removal). Individual gene trees were reconstructed using RAxML version 8 [42] with the GTRGAMMA model and 1000 rapid bootstrap replicates. All gene and species trees are available from the Dryad Digital Repository: doi:10.5061/dryad.hj6r1.

### Species Delimitation Analyses

We use the general lineage concept (as defined in [2]) as our theoretical concept of a species and utilize multiple analyses to provide supporting criteria with which we can operationally delimit general lineage species. Groups of samples showing concordance across different data sets and analyses (identified as different morphometric clusters, highly supported mtDNA clades, validated nuclear clades, and distinguishable via general morphology) are considered species [43]. A general workflow for the species delimitation decision-making process is provided in FIGURE 1.

The Bayesian program BPP [8], [44] was used to test specific species delimitation hypotheses. This program conducts multilocus, coalescent-based analyses requiring a guide tree and specification of two priors controlling population size and divergence time. The program incorporates a reversible-jump Markov chain Monte Carlo algorithm (rjMCMC) that explores all possible species delimitation models, ultimately providing an assessment of the probability of a node being present. Although BPP is known to recover a higher number of partitions relative to other programs, particularly for micro-allopatric taxa with deep genetic divergences, we believe that BPP used in conjunction with discovery-based analyses can lead to informed, conservative decisions about species delimitations [16]. Specific hypotheses tested included the following: *S. nondimorphicus*+*S. robustus idahoensis*, *C. ungulatus ungulatus*+*C. u. madhousensis*, Cave of the Winds+Mallory Cave, and within the *S. r. glorietus* complex: *S. r. glorietus* (Glorieta Canyon, New Mexico)+*S. r. glorietus* (Taos Ski Valley), southern *S. r. glorietus*+*S. r. glorietus* (Glorieta Canyon), and the syntopic surface and troglomorphic populations of *S. r. glorietus* from Taos Ski Valley. The species tree recovered from *BEAST analyses based on morphological clusters was used as the input guide tree. In BPP, the rjMCMC species delimitation method [44] was used with algorithm 0 (ε = 10), rates were allowed to vary among loci (locusrate = 1), gaps and ambiguous columns were removed (cleandata = 1), and the analysis was set for automatic fine-tune adjustments. Four different combinations of theta (θ) and tau (τ) priors were used to span a diversity of possible population sizes and divergence times. Each prior combination was run twice to check convergence of runs and proper mixing. Analyses were run for 200,000 generations, sampling every 5 generations with 20,000 burnin. Posterior probabilities greater than 95 for the presence of a node are considered supported species delimitations.

Using the gene trees estimated with RAxML, we calculated the genealogical sorting index (*gsi*) for each gene and the ensemble genealogical sorting index (*gsi_T_*) [45] using the *gsi* website (http://www.genealogicalsorting.org) with 10,000 replicates. The *gsi* statistic assesses the level of genealogical exclusivity of a group where values range from 0 (a random distribution of haplotypes) to 1 (exclusive ancestry, monophyly), and the ensemble analysis (*gsi_T_*) incorporates uncertainty of relationships and integrates across all gene trees. The groups assessed consisted of the putative species identified via discovery-based analyses (FIGURE 1).

Based on multivariate morphological clustering, there is evidence that the *S. r. glorietus* subspecies comprises more than one species; however, species limits are uncertain due to sparse geographic sampling. It is important to note that the subspecies *S. r. glorietus* was described from only a single locality, Glorieta Canyon, New Mexico. As such, and because one of the highly troglomorphic populations is recovered *within* the *S. r. glorietus* complex, we simultaneously estimated species limits and species trees for this complex using the Bayes factor delimitation (BFD) method of Grummer et al. [46], testing several different species limit combinations (FILE S3). This method, based on Bayes factors, is beneficial in cases where species limits are uncertain as topological uncertainty is accounted for, and *a priori* species relationships are not required [46]. Additionally, we ran a *BEAST analysis in which each individual *S. r. glorietus* population was considered a separate species, then tested each species (population) using BPP (FILE S3).

### Taxonomy

Digital images were taken using a Visionary Digital system (http://www.visionarydigital.com). Several images were taken at different focal planes and combined using Zerene Stacker (Zerene Systems LLC). Scanning electron microscopy was conducted using methods outlined in Derkarabetian et al. [22]. Leg and pedipalp illustrations were done using a drawing tube attached to an Olympus SZX12 microscope with subsequent tracing in Adobe Illustrator CS5. Penis illustrations were traced from digital images. Digital images have been deposited to Morphbank; image ID numbers are included in the figure legends. A Google Earth kmz file with all known localities including sites from this study, previous studies, and geo-referenced museum records for each species is included as FILE S4. All relevant information will be integrated into Encyclopedia of Life webpages (http://www.eol.org) for each species.

### Nomenclatural Acts

The electronic edition of this article conforms to the requirements of the amended International Code of Zoological Nomenclature, and hence the new names contained herein are available under that Code from the electronic edition of this article. This published work and the nomenclatural acts it contains have been registered in ZooBank, the online registration system for the ICZN. The ZooBank LSIDs (Life Science Identifiers) can be resolved and the associated information viewed through any standard web browser by appending the LSID to the prefix “http://zoobank.org/”. The LSID for this publication is: urn:lsid:zoobank.org:pub:46DB0A1A-E317-4CD3-A124-53E97418C4B0. The electronic edition of this work was published in a journal with an ISSN, and has been archived and is available from the following digital repositories: PubMed Central, LOCKSS.

## Results

### Taxon Sampling

**Figure 2:**
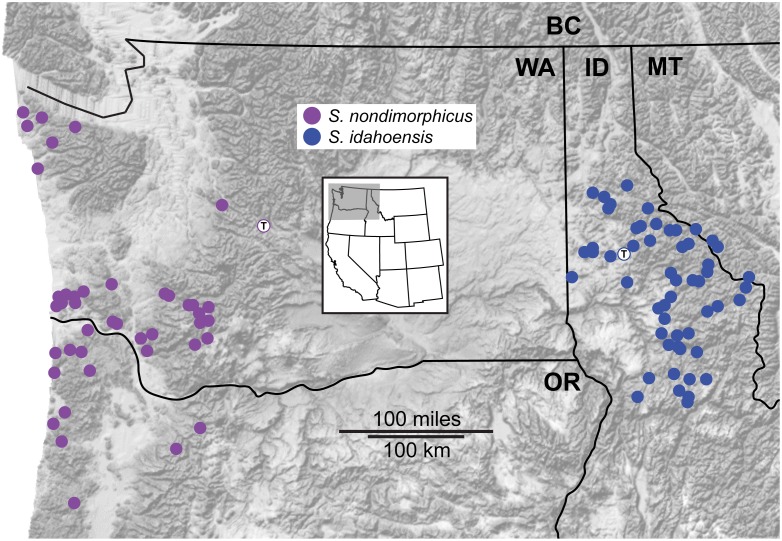
Geographic sampling of the *nondimorphicus* group. Localities are those from recent fieldwork. Localities with “T” correspond to type localities.

**Figure 3:**
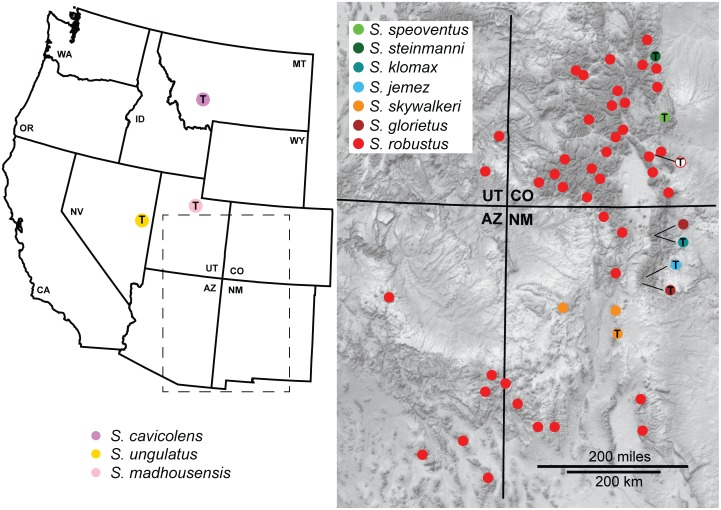
Geographic sampling of the *cavicolens* and *robustus* groups. Localities are those from recent fieldwork. Localities with “T” correspond to type localities. Type localities with solid circles correspond to localities sampled during this study.

All collecting localities from recent fieldwork for this study, including specimens used in morphological and/or genetic analyses, are shown in FIGURES 2 and 3.

### Morphometric Analyses

**Figure 4:**
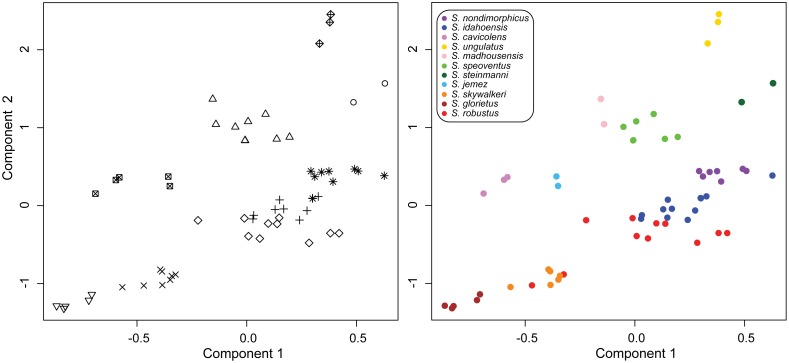
Multivariate clustering results. Left panel: specimens grouped by delimited clusters. Right panel: specimens colored by species (both previously and newly described).

The results of the traditional PCA and DFA are presented in FILE S1. Here, we present the results of the multivariate clustering method of Ezard et al. [7]. The analysis of male specimens retained 3 components (FIGURE 4, FILE S5) and selected the EEE model (ellipsoidal, equal volume, shape and orientation) with 9 clusters as optimal. Clusters correspond to *ungulatus*, Mallory Cave, Cave of the Winds+*madhousensis*, *cavicolens*+Terrero Cave, northern *glorietus* (Taos Ski Valley and Glorieta Canyon), southern *glorietus*+*robustus* from Bradford Canyon, New Mexico, and three separate clusters largely corresponding to *nondimorphicus*, *idahoensis*, and *robustus*. The recovery of the Bradford Canyon *robustus* specimens with the generally smaller-bodied *glorietus* subspecies is not surprising considering Bradford Canyon specimens have the smallest body size of any *robustus* population known. The *nondimorphicus* cluster included 2 specimens of *idahoensis*, one of which is the largest *idahoensis* sampled (Goose Creek, Montana). Two specimens of *ungulatus* were considered significant outliers but were not removed from analyses.

Analysis of female specimens retained 2 components (FILE S5) and the optimal model chosen was the EEE model with 4 clusters corresponding to *glorietus* (northern and southern), *robustus*+*idahoensis*+*nondimorphicus*, Cave of the Winds+Mallory Cave, and *cavicolens*+Terrero Cave+Taos Ski Valley troglomorph. Although we include female morphometric analyses, we mainly rely on results of the male analyses for downstream decisions, as males are more variable and easily distinguished.

### Molecular Data Collection

GenBank accession numbers and the final genetic dataset are shown in TABLE S3 and alignment statistics are provided in TABLE 1. In total, original data for 8 nuclear gene regions were gathered from 36 *Sclerobunus* and 7 *Cyptobunus* samples. For each locus, no more than 5 samples were missing data (average of 2 sequences missing per gene), and no single specimen had more than one missing gene region (in total, 4.3% missing data).

**Table 1:** Nuclear gene data alignment and diversity statistics.

Locus	Region	n	Length	VarS	PIS	s	N	H	θ (site)	θ (seq)	R	Taj D	BIC
TPR	Exon	68	917 (784)	153	135	153	50	0.988	0.0407	31.946	0.6	0.21135	HKY+G
CHP1	Exon	52	901 (752)	120	103	120	36	0.983	0.03531	26.556	3.8	0.48029	TrN+G
CHP2	Exon	52	708 (673)	96	85	96	40	0.988	0.03157	21.245	1.4	0.07763	K80+G
nrm	3′UTR	49 (56)	978 (591)	91	76	91	31	0.974	0.03453	20.409	1	−0.09569	HKY+G
DNO	Exon	59	645 (560)	92	81	92	44	0.989	0.03536	19.801	3.7	−0.21795	HKY+G
SKI	Exon	68	701 (684)	142	124	142	57	0.993	0.04335	29.649	0.7	−0.10803	GTR+I+G
ADARB2	Exon	54	839 (784)	97	90	97	35	0.978	0.02715	21.286	0.6	0.41108	K80+G
PGM	3′UTR	41 (49)	591 (313)	120	105	120	22	0.968	0.08961	28.047	6.6	−0.04529	HKY+G

**Notes:** Locus abbreviations in File S2. n, number of phased sequences (for UTRs, number of total sequences); length, length of alignment (length with any missing sites removed); VarS, variable sites; PIS, parsimony informative sites; s, segregating sites; N, number of haplotypes; H, haplotype diversity; θ (site), theta per site; θ (seq), theta per sequence; R, recombination value; Taj D, Tajima's D (no significant values); BIC, model chosen with PartitionFinder [38].

### Species Tree Analyses

**Figure 5:**
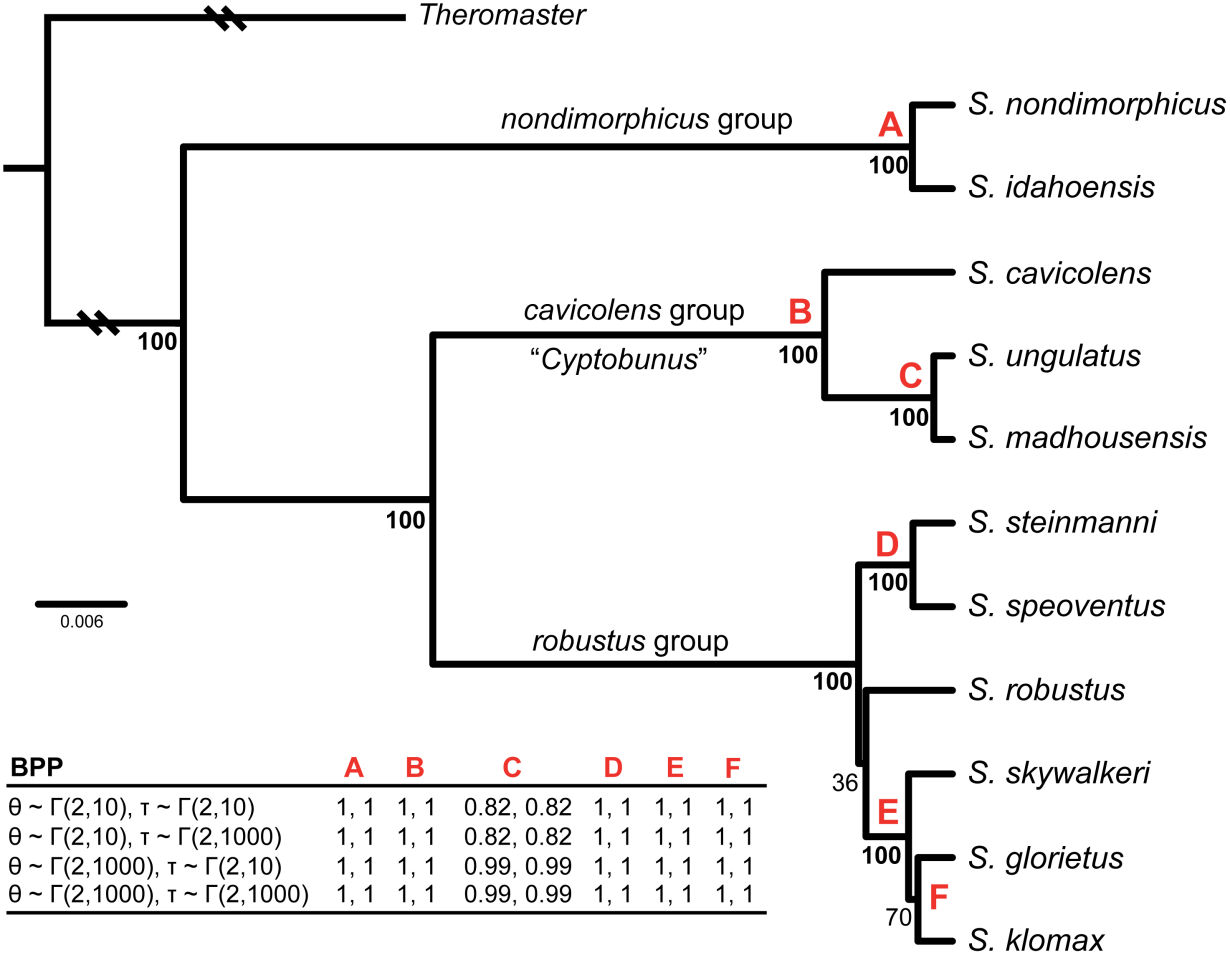
*BEAST species tree with BPP species delimitations. Numbers below nodes on species tree correspond to posterior probability support. Earliest diverging braches shortened for graphical purposes. At bottom left, BPP results with 4 different prior combinations. Numbers correspond to results of the two separate BPP runs. Red letters correspond to those nodes on the species tree.

Model and partition selection via BIC resulted in single partitions for each gene region (TABLE 1). Individual RAxML gene trees are included in FILE S6. Preliminary rooting analyses recovered northwestern *Sclerobunus* (*nondimorphicus* and *idahoensis*) as sister to all remaining lineages. Nodes were considered highly supported if the posterior probability values exceeded 95. In the species tree (FIGURE 5), all nodes were resolved with 100% posterior probability, with the exception of the node containing the southwestern *Sclerobunus* and the node between *glorietus* and *klomax*. The genus *Cyptobunus* is phylogenetically nested within *Sclerobunus* with 100% posterior probability. Therefore, as the generic name *Sclerobunus* has precedence [26], we synonymize *Cyptobunus* with *Sclerobunus*, and all species of *Cyptobunus* are transferred to *Sclerobunus* (see below).

The fact that the species tree is well resolved, with the exception of two nodes, confirms the presence of three species groups recovered in previous analyses [21], [22] and helps resolve those morphological clusters containing more than one putative species. In this regard, morphometric clusters containing species from different species groups are easily separated. In the male analysis (FIGURE 4) a single specimen of *idahoensis* was recovered within a cluster comprised of mostly *robustus* specimens, a cluster included all *cavicolens* and Terrero Cave specimens, and another cluster included all Cave of the Winds and *madhousensis* specimens. Similarly in the female analysis, a single cluster included the Taos Ski Valley troglomorph, Terrero Cave, and *cavicolens* specimens (FILE S5). As these clusters contain taxa from different species groups, they do not represent potential conspecific taxa clusters, but instead represent cases of morphological convergence across distantly related taxa. The two clusters including multiple taxa from the same species group (*nondimorphicus*/*idahoensis* and *robustus*/southern *glorietus*) were not delimited at this step and relied upon validation methods. Taken together, the morphometric and species trees analyses, coupled with previous genetic analyses, conservatively support *at least* eight putative species corresponding to *nondimorphicus*/*idahoensis*, *cavicolens*, *ungulatus* (two subspecies), Cave of the Winds/Mallory Cave, *robustus*, southern *glorietus*, northern *glorietus*, and Terrero Cave/Taos Ski Valley troglomorph.

### Species Delimitation

Results of the BPP species delimitation analyses are shown in FIGURE 5. All species, including all putative new species and morphometric clusters containing multiple putative species from the same species group, were delimited with 100% posterior probability in all runs under all prior combinations. The two subspecies of *ungulatus* are an exception. Morphological clustering methods showed a clear distinction between the two subspecies of *ungulatus*. However, in the delimitation analyses with higher values of theta, θ∼Γ(2, 10), indicative of large effective population sizes, the presence of the node was not supported. For cave species, which generally have small populations sizes, the analyses with low values of theta, θ∼Γ(2, 1000), may be more appropriate priors for delimitation. Additionally, these isolated cave populations are separated by over 240 kilometers of arid, uninhabitable terrain where dispersal and gene flow is impossible. This extreme isolation clearly supports these two subspecies as separately evolving lineages. As such, we elevate both subspecies to full species: *S. ungulatus* **comb. nov.** and *S. madhousensis* **comb. nov.**, **stat. nov.** Morphometric clustering methods (FIGURE 4) and genetic delimitation analyses (FIGURE 5) distinguished the new species *S. speoventus* **stat. nov.** (Cave of the Winds) from *S. steinmanni* **stat. nov.** (Mallory Cave). Although the *S. madhousensis* specimens clustered with *S. speoventus*, these two taxa are from different species groups (FIGURE 4) and are qualitatively morphologically different. Results of *gsi* analyses show exclusive ancestry for all species in at least 5 of 8 loci, with the exception of *S. robustus*, which was exclusive for only 3 of 8 loci and S. *nondimorphicus*, *S. idahoensis*, and *S. glorietus*, which were non-exclusive for all loci (TABLE 2). The *gsi_T_* is greater than 0.5 for all species and greater than 0.8 for 8 of 11 species, demonstrating that a high degree of genealogical sorting has occurred in these species and they are progressing towards exclusive ancestry.

**Table 2:** Results of *gsi* analyses.

	TPR	CHP1	CHP2	nrm	DNO	SKI	ADARB2	PGM	*gsi_T_*
*S. cavicolens*	1	1	1	1	1	1	1	1	1
*S. madhousensis*	1	1	1	1	1	1	1	1	1
*S. ungulatus*	1	1	1	1	1	1	0.5765	1	0.8221
*S. nondimorphicus*	0.4516	0.6232	0.5357	0.6562	0.5874	0.5203	0.6851	0.5814	0.5801
*S. idahoensis*	0.8607	0.6839	0.5357	0.3774	0.6878	0.8201	0.395	0.4783	0.6048
*S. robustus*	0.8712	0.9088	0.7041	1	0.8561	1	0.8421	1	0.8978
*S. glorietus*	0.4426	0.4694	0.5755	0.6405	0.5403	0.6344	0.64	0.6811	0.578
*S. klomax*	0.4925	1	1	1	1	0.4022	1	1	0.8618
*S. skywalkeri*	1	1	1	1	0.5786	0.5446	0.3769	1	0.8125
*S. speoventus*	1	1	1	1	1	1	1	1	1
*S. steinmanni*	1	1	1	1	1	1	1	1	1

**Notes:** Values for individual genes are from individual *gsi* analyses. All values are significant (p-value<0.05).

**Figure 6:**
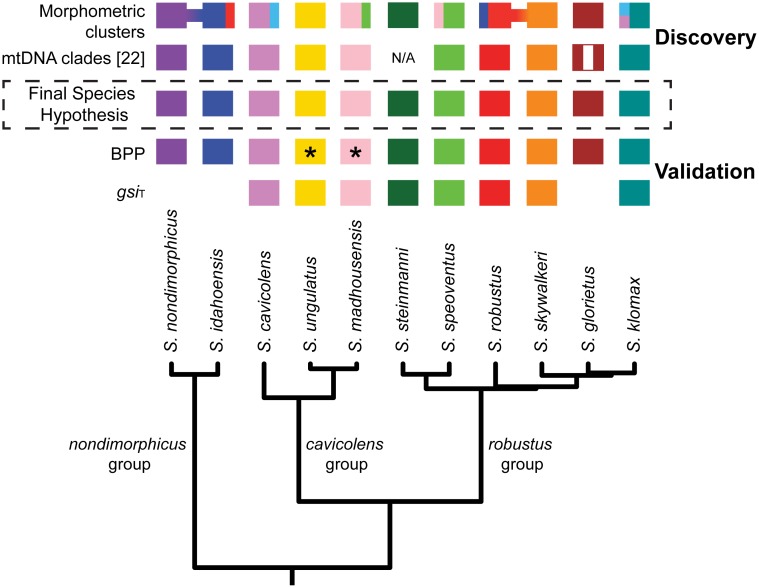
Results of integrative species delimitation. Solid color boxes indicate successful identification or delimitation of a species for the particular approach. For the morphometric clustering method, connected boxes indicate clusters including specimens from multiple species within the same species group. Clusters including specimens from multiple species from different species groups are indicated with multicolor boxes. For the mtDNA analyses [22], the vertically striped box for *S. glorietus* indicates paraphyly. For *S. steinmanni*, only one specimen was sequenced, so mitochondrial monophyly could not be assessed. For BPP analyses, asterisks indicate that *S. ungulatus* and *S. madhousensis* were not supported in analyses with certain prior combinations (see Results). Only species with *gsi_T_* values greater than 0.8 are indicated.

A summary of all species delimitation analyses in the context of an integrative taxonomic framework is shown in FIGURE 6. Results presented here support the presence of five new species and the elevation of all three subspecies of *S. robustus: S. robustus*, *S. glorietus* **stat. nov.**, and *S. idahoensis* **stat. nov.** The species *S. idahoensis*, a former subspecies of *S. robustus*, is not sister to other *S. robustus* subspecies, and was successfully delimited from *S. nondimorphicus* (see Taxonomy for details). Within the *S. glorietus* complex three species were delimited based on morphology (FIGURE 4): a northern clade (*S. glorietus* **stat. nov.**), a southern clade (*S. skywalkeri* **sp. nov.**), and the highly troglomorphic population from Taos Ski Valley (*S. klomax* **sp. nov.**). Although BFD analyses slightly favor including the Glorieta Canyon population with the southern *S. glorietus* clade (FILE S3), the support is not strong (both BF and posterior probability). Species tree analyses in which each population of *S. glorietus* are considered putative species resulted in little support for relationships within the complex, except for *S. skywalkeri* (FILE S3).

We also describe a new troglomorphic species from Terrero Cave, *S. jemez* **sp. nov.** Genetic data for this cave population were not available for this study and access to the cave to collect fresh specimens could not be granted, as the cave is culturally significant to the Jemez Pueblo. Although the *S. jemez* specimens clustered with the *S. cavicolens* specimens in male and female morphometric analyses (FIGURE 4, FILE S5), these taxa are from different species groups and easily distinguished morphologically. In the female morphometric analyses (FILE S5), the *S. jemez* specimens additionally clustered with the *S. klomax* specimens. These two species can be differentiated based on several characters (see below). Geographic evidence also supports this distinction as these caves are separated by over 90 km.

### Taxonomy

**Figure 7:**
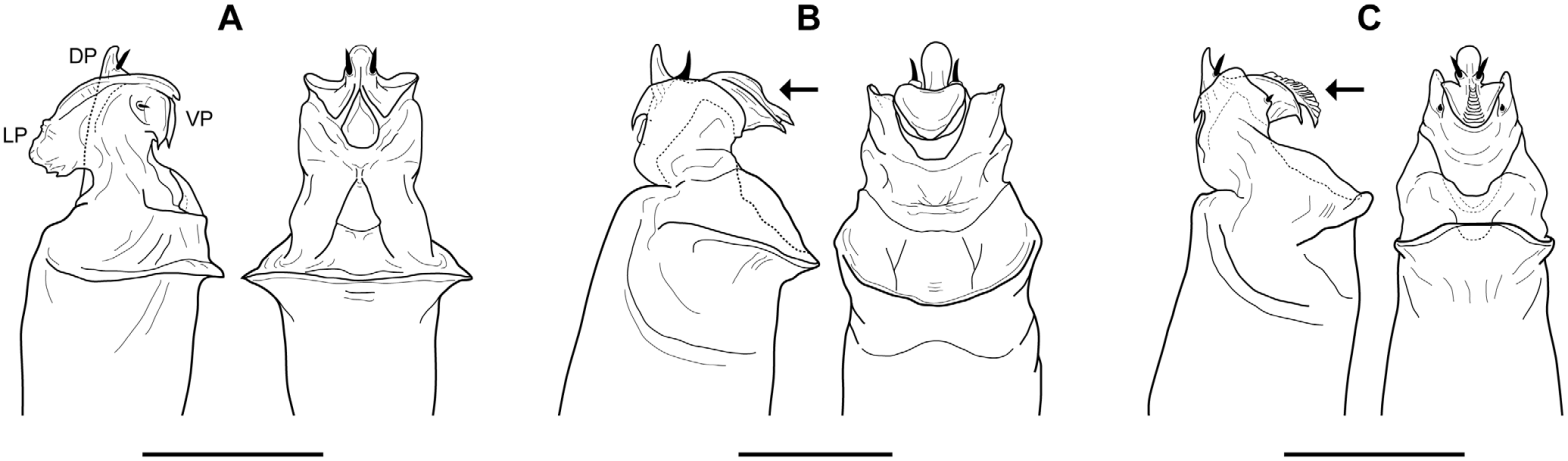
Comparative penis morphology for *Sclerobunus* species groups. A) *cavicolens* group - lateral and ventral views, *S. ungulatus* (Model Cave, NV). B) *nondimorphicus* group - lateral and ventral views, *S. idahoensis* (Meadow Creek, ID), arrow indicates smooth dorsal surface of the ventral plate. C) *robustus* group - lateral and ventral views, *S. robustus* (Chiricahua Mountains, AZ), arrow indicates dorsal surface of the ventral plate with many folds. DP = dorsal plate, LP = lateral plates, VP = ventral plate. Scale bars = 0.25 mm.

**Figure 8:**
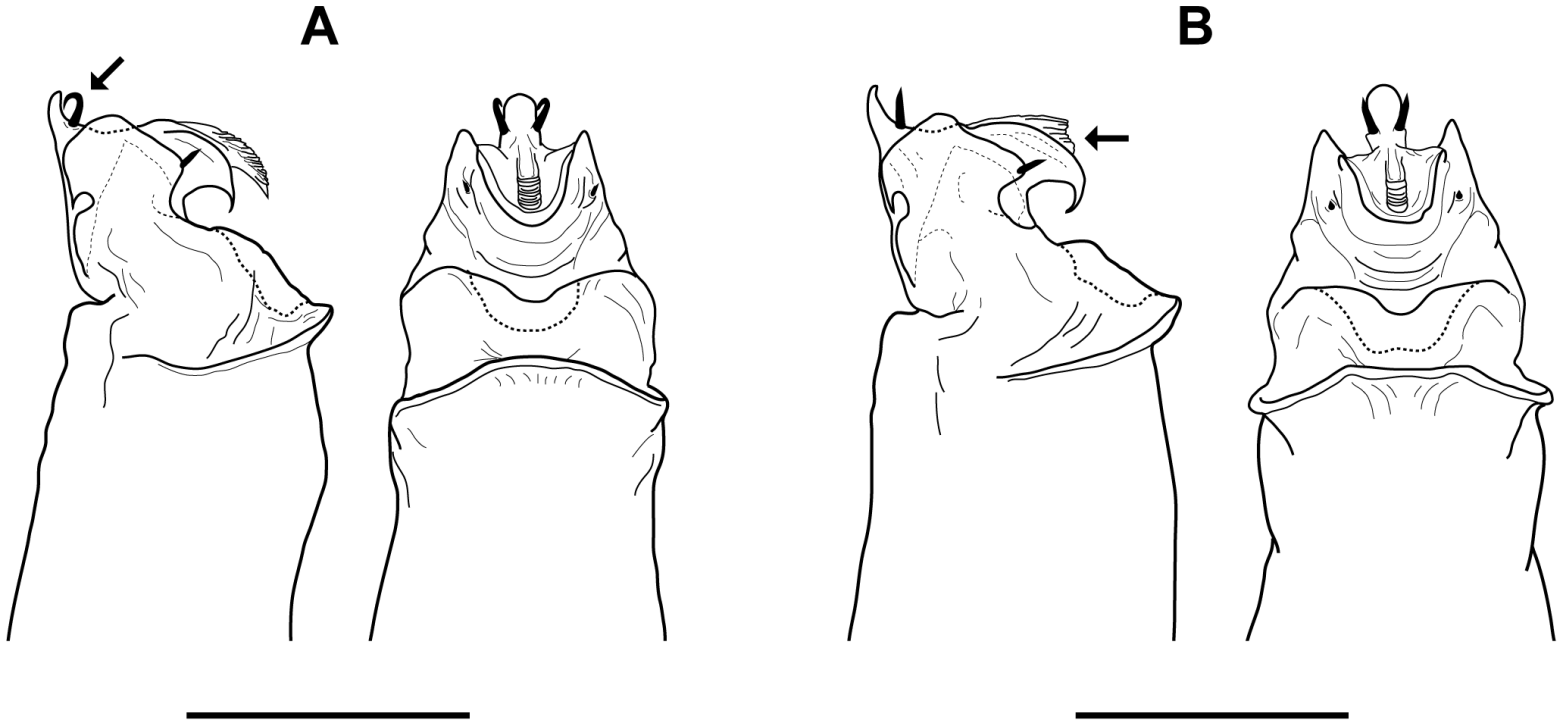
Comparative penis morphology for *S. speoventus* and *S. steinmanni*. A) lateral and ventral views, *S. speoventus*, paratype (Cave of the Winds, CO), arrow indicates curved subapical spines. B) lateral and ventral views, *S. steinmanni*, holotype (Mallory Cave, CO), arrow indicates dorsoventrally compressed ventral plate. Scale bars = 0.25 mm.

**Figure 9:**
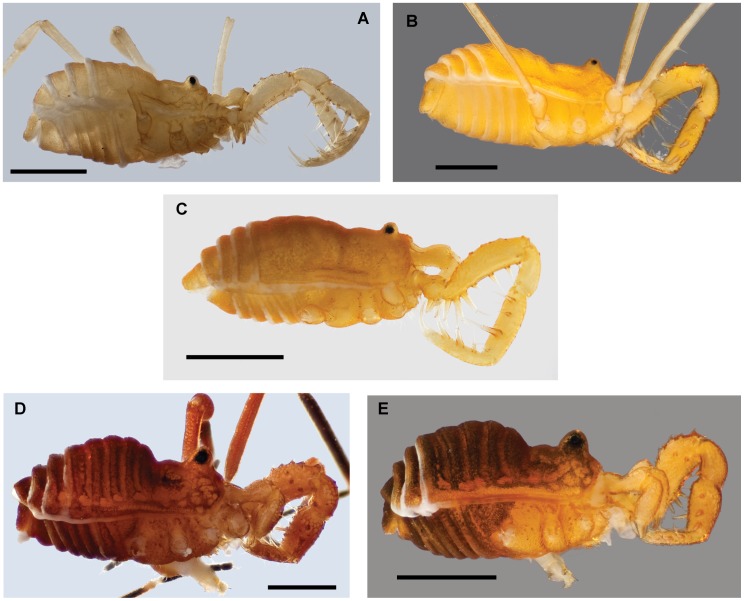
Habitus morphology of the *nondimorphicus* and *cavicolens* groups. A) *S. cavicolens*, male, image horizontally reflected (Lewis and Clark Caverns, MT) [835849]. B) *S. ungulatus*, male (Model Cave, NV) [835874]. C) *S. madhousensis*, male (North Madhouse Cave, UT) [835858]. D) *S. nondimorphicus*, male, image horizontally reflected (Iron Creek, WA) [835861]. E) *S. idahoensis*, male (Hobo Cedar Grove, ID) [835854]. Scale bars = 1 mm. Morphbank numbers indicated in brackets.

**Figure 10:**
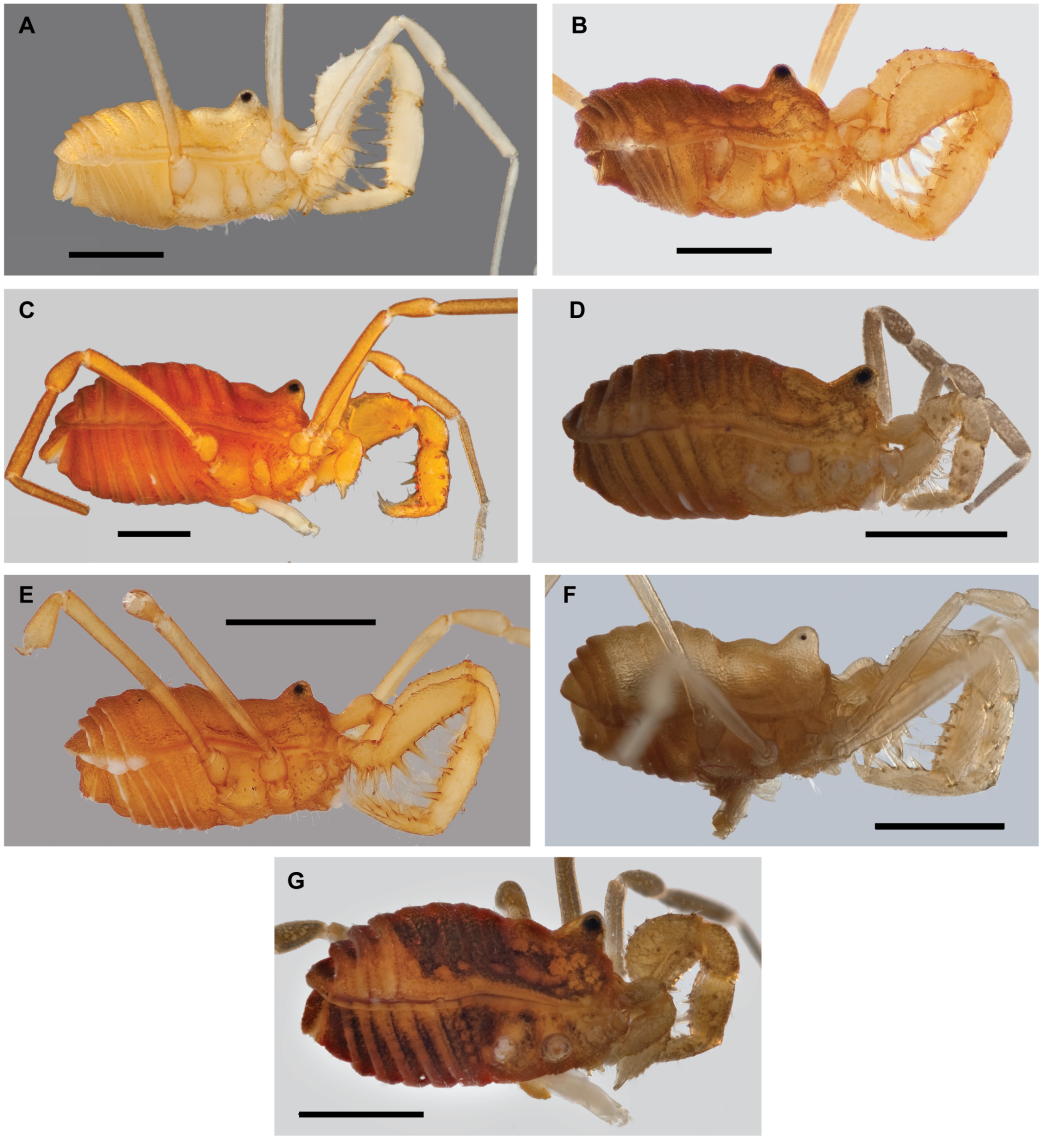
Habitus morphology of the *robustus* group. A) *S. speoventus*, paratype male (Cave of the Winds, CO) [835868]. B) *S. steinmanni*, holotype male (Mallory Cave, CO) [835871]. C) *S. robustus* male (Apex Valley, CO) [835865]. D) *S. glorietus*, male (Glorieta Canyon, NM) [835850]. E) *S. klomax*, holotype female (Taos Ski Valley, NM) [835857]. F) *S. jemez*, holotype male (Terrero Cave, NM) [835856]. G) *S. skywalkeri*, paratype male, image horizontally reflected (Manzano Mountains, NM) [835867]. Scale bars = 1 mm. Morphbank numbers indicated in brackets.

Here, we synonymize, redescribe, and elevate all *Cyptobunus* under *Sclerobunus* (*S. cavicolens* **comb. nov.**, *S. ungulatus* **comb. nov.**, and *S. madhousensis* **comb. nov.**, **stat. nov.**), redescribe and elevate all three *S. robustus* subspecies to full species (*S. robustus*, *S. glorietus* **stat. nov.**, and *S. idahoensis* **stat. nov.**), and describe five new *Sclerobunus* species (*S. jemez* **sp. nov.**, *S. klomax* **sp. nov.**, *S. skywalkeri* **sp. nov.**, *S. speoventus* **sp. nov.**, and *S. steinmanni* **sp. nov.**). Additionally, the previously unknown males of *S. madhousensis* are described. Penis morphology in *Sclerobunus* is relatively conserved within species groups; detailed examination within and between the *S. robustus* and *S. glorietus* species show very little variation [22]. However, diagnostic differences in penis morphology can be seen between the three species groups recovered in all phylogenetic analyses (FIGURE 7), hereafter referred to as the *cavicolens*, *nondimorphicus*, and *robustus* species groups. At the species level, only *S. speoventus* and *S. steinmanni* show diagnostic differences in penis morphology (FIGURE 8). Despite little genitalic divergence within species groups, conspicuous variation can be seen in somatic morphology (FIGURES 9 and 10, FILES S7, S8, and S9).

Abbreviations for museums and collections: AMNH = American Museum of Natural History, CAS = California Academy of Sciences, CHR = personal collection of Casey H. Richart, DMNS = Denver Museum of Nature and Science, SDSU = San Diego State University Terrestrial Arthropod Collection. DNA vouchers held in the SDSU collection are indicated by “OP” numbers. With the exception of commercial caves, GPS coordinates for cave localities, including type localities, are purposefully withheld for the protection of those cave habitats. Additionally, cave waypoints in FILE S4 are approximate.

Morphological abbreviations: CI = coxae I, CII = coxae II, DCS = distal cheliceral segment, GO = genital operculum, LI = leg I, LII = leg II, LII/SL = leg II total length to scute length ratio, OC = ocularium, PCS = proximal cheliceral segment, PF = pedipalpal femur, PT = pedipalpal tibia, SBT = spine-bearing tubercle. All measurements are in millimeters.

***Sclerobunus*** **Banks, 1893**

*Scotolemon* [part] Packard, 1877: 164 [25]

*Phalangodes* [part] Packard, 1888: 48 [47]

*Sclerobunus* Banks, 1893: 152 [26]; Banks, 1901: 672 [48]; Banks, 1911: 415 [49]; Roewer, 1915: 87 [31]; Roewer, 1923: 596–597 [50]; Crosby and Bishop, 1924: 104 [32]; Goodnight and Goodnight, 1943: 645–646 [33]; Dumitrescu, 1976: 20 [51]; Briggs, 1971: 8 [28]; Shear, 1977: 178 [52]; Edgar, 1990: 540 [53]; Kury, 2003: 18 [54]; Derkarabetian et al., 2010 [21]; Derkarabetian et al., 2011 [22]

*Cyptobunus*, Banks, 1905: 251–252 [30]; Roewer, 1915: 62 [31]; Roewer, 1923: 631 [50]; Roewer, 1931: 152–154 [27]; Briggs 1971: 8–9 [28]; Shear, 1977: 178 [52]; Edgar, 1990; 540 [53]

*Cryptobunus* [misspelling], Rambla and Juberthie, 1994: 222 [55]

**Type Species**: *Sclerobunus robustus* (Packard, 1977)

**Diagnosis**. *Sclerobunus* can be differentiated from other travunioid genera by the combination of a penis having: lateral plates extending dorsally; lingulate ventral plate extending perpendicular to dorsal plate, with acute apex (lateral view); dorsal plate rounded distally, slightly curved, with a single pair of subapical lateral spines.

**Description**. Body length 1.68–3.68, length of scute 1.58–2.69. Body surface structure microgranulate-rivulose, integument color reddish/brown, yellow/orange to yellow in troglomorphic species, with or without black pigment. Anterior margin of scute with 1–4 tubercles on each shoulder; highly troglomorphic species without tubercles. OC recessed from anterior margin. Pedipalps strongly armed with many spine-like tubercles each bearing elongate setae subapically; first 3 proximal ventral spines of femur larger followed by 3 or more smaller spines; patella with 2 medial SBTs at distal margin; tibia with row of 4–6 medial spines and row of 4–7 lateral spines; tarsus with 4 large lateral and medial spines. LI femur with ventral row of 3 or more SBTs.

**Sexual Dimorphism.** As noted by Briggs [28], the PF is noticeably thicker in males of all *Sclerobunus* (except *S. nondimorphicus*). We note two additional sexually dimorphic characters. First, in males the CII lobes found along the ventral midline possess 2–3 pairs of apophyses (FILE S7d), while female CII lobes do not possess apophyses. Second, males possess a small distal process on the ventral surface of the pedipalpal tarsus (FILE S7e). Males of the *S. nondimorphicus* group may additionally possess many smaller asetose tubercles in 2 rows extending proximally.

**Distribution.** Mountainous regions of western North America (Arizona, New Mexico, Colorado, Utah, Nevada, Montana, Idaho, Oregon, Washington, and British Columbia).

**Key to species groups of** ***Sclerobunus***

1. Penis with lateral plates extending dorsally into fan-shaped projections, ventral plate compressed dorsoventrally (FIGURE 7a); anterior margin of scute without tubercles on shoulders; isolated caves in Montana, Nevada, and Utah ( = *Cyptobunus*)… ***S. cavicolens*** **group**
  Penis with lateral plates extending dorsally to an elongate process with acute apex, ventral plate more elongate (FIGURE 7b,c); anterior margin of scute with at least one tubercle on shoulders (FILE S7f) … **2**
2. Penis with dorsal surface of ventral plate smooth (FIGURE 7b); Pacific Northwest (WA, OR, BC, ID, MT)… ***S. nondimorphicus*** **group**
  Penis with dorsal surface of ventral plate with many folds (FIGURES 7c and 8); southwestern North America (AZ, NM, UT, CO)… ***S. robustus*** **group**

***Sclerobunus cavicolens*** **group**

**Diagnosis.** Penis with lateral plates extending dorsally into fan-shaped projections and extending ventrally into bifurcate plate, ventral plate compressed dorsoventrally and reduced in size (FIGURE 7a). No tubercles on the anterior margin of the scute.

**Included species.** *S. cavicolens* (Banks), *S. ungulatus* (Briggs), *S. madhousensis* (Briggs).

**Distribution.** Isolated caves and cave systems in Montana, Nevada, and Utah.

**Key to adults of** ***S. cavicolens*** **group**

1. Scute length under 1.7 mm; PF with 6 ventral SBTs, pedipalpal tibia with 4 large retrolateral spines (FILE S8a); LII length less than 9 mm; Lewis and Clark Caverns, MT… ***S. cavicolens*** **comb. nov.**
  Scute length over 1.7 mm; PF with 7 or more ventral SBTs, pedipalpal tibia with 5 or more retrolateral spines (FILE S8b,c); LII length over 9 mm… **2**
2. Scute length 1.7–2 mm; LII length under 12 mm; caves near Provo, UT… ***S. madhousensis*** **comb. nov., stat. nov.**
  Scute length over 2 mm; LII length over 13 mm; caves in Great Basin National Park, NV… ***S. ungulatus*** **comb. nov.**

***Sclerobunus cavicolens*** **(Banks, 1905)**

Figures: **map** 3a; **habitus** 9a; **pedipalp** S8a; **leg I** S9a

*Cyptobunus cavicolus*, Banks 1905: 252 [30], fig 1; Roewer, 1915: 62 [31], 167; Roewer, 1923: 631 [50]; Roewer, 1931: 152 [27]

*Sclerobunus cavicolens*, Crosby and Bishop 1924: 104 [32]; Goodnight and Goodnight 1943: 646–647, fig 8–9 [33]; Goodnight and Goodnight, 1960; 37 [56]

*Sclerobunus robustus* [part], Roewer, 1931: 153 [27]

*Cytobunus cavicolens* [misspelling], Goodnight and Goodnight, 1943: 646 [33]

*Cyptobunus cavicolens*, Briggs 1971: 4–5, figs 1–9, map 1 [28]; Edgar, 1990: 540 [53]; Kury, 2003: 18 [54]

**Type Material. Holotype** male collected from a cave near Limespur, Montana (Lewis and Clark Caverns, formerly Morrison Cave: N45.8386, W111.8668), depth of 190 feet, juvenile, 1905 (MCZ, not examined). For descriptions, Briggs [28] and Goodnight and Goodnight [33] used additional specimens collected from the type locality: Big Spring Room and Cathedral Room of Morrison Cave (Lewis and Clark Caverns), 60 miles west of Bozeman, Montana, February 22, 1941 collected by H. B. Mills and A. L. Jellison (AMNH, not examined).

**Diagnosis.** Compared to other species in the *S. cavicolens* group, *S. cavicolen*s is less troglomorphic, with present but highly reduced lateral prongs on the hind claws. Diagnosed based on small body size (scute length <1.7 mm), shorter legs (LII <9 mm), and only 4 spines on pedipalpal tibia.

**Description.** MALE: (N = 3). Body length 1.8–2.4, scute length 1.58–1.68, greatest width of anterior scute 1.06–1.09, greatest width of opisthosoma 1.65–1.73. Integument yellow, with very faint black pigment. OC height 0.08, width 0.22–0.23. OC low, rounded. Pedipalpal coxae with 1 SBT at distal margin (lateral edge). CI with 7 spines, some on tubercles. CII lobes with 2 apophyses. GO length 0.3–0.32, width 0.37–0.38. PCS greatest width 0.21, single dorsal SBT at distal edge, 1–2 small ventrolateral SBTs at distal margin. DCS length 0.67–0.71, greatest width 0.15–0.21. PF height 0.3–0.31, with row of 5 small dorsal SBTs, 1–3 mesodorsal SBTs, 3 medial spines, row of 6 ventral spines; PT with 4 medial spines, 5 lateral spines. LI femur with row of 3–4 SBTs; tibia usually with 1 small tubercle, but can be without. LII total length 8.87–8.95: trochanter 0.34–0.35, femur 2.1, patella 0.63–0.64, tibia 1.79–1.82, metatarsus 1.88–1.94, tarsus 2.11–2.15. LII/SL 5.3–5.68.

FEMALE: (N = 1). Body length 2.23, scute length 1.67, greatest width of anterior scute 1.01, greatest width of opisthosoma 1.78. OC height 0.09, width 0.23. GO length 0.29, width 0.39. PCS width 0.21. DCS length 0.64, width 0.22. PF height 0.26. LII total length 8.38: trochanter 0.41, femur 1.94, patella 0.68, tibia 1.66, metatarsus 1.76, tarsus 1.93. LII/SL 5.02.

**Material Examined.** MONTANA: **Jefferson Co.:** Morrison Cave (Lewis and Clark Caverns), Big Spring Room, 60 miles west of Bozeman (N45.8386, W111.8668), 22 February 1941, H.B. Mills, 1 male, 2 adults (AMNH); Lewis and Clark Caverns, on walls in Paradise Room (N45.8386, W111.8668), 4 July 2008, S. Derkarabetian, C. Richart, J. Underwood, 2 males, 1 female, 1 juvenile (SDSU: OP2143–OP2145).

**Distribution.** Only known from the type locality.

**Genetic Data.** <3?tlsb=-.02w?>GenBank accession numbers: KJ585335–KJ585337, KJ585089–KJ585091, KJ585129–KJ585131, KJ585212–KJ585214, KJ585170–KJ585172, KJ585292–KJ585294, KJ585046–KJ585048, KJ585253–KJ585255.

***Sclerobunus madhousensis*** **(Briggs, 1971), comb. nov., stat. nov.**

Figures: **map** 3a; **habitus** 9c; **pedipalp** S8c; **leg I** S9c

*Cyptobunus ungulatus madhousensis*, Briggs 1971: 6, figs 19–25, map 1 [28]; Kury, 2003: 18 [54]

*Cytobunus ungulatus madhousensis* [misspelling], Edgar, 1990: 541, fig 19.18 [53]

*Cryptobunus ungulatus madhousensis* [misspelling], Rambla and Juberthie, 1994: 222 [55]

**Type Material. Holotype** female, North Madhouse Cave, near Provo, Utah (GPS withheld), 27 May 1965, Stan Moulton (AMNH, not examined).

**Diagnosis.** *S. madhousensis* can be differentiated from *S. cavicolens* by a complete lack of pigment, lack of prongs on hind claws, pedipalpal tibia with 5 or more medial spines. Diagnosed from *S. ungulatus* by the shorter length of LII (10–12 mm).

**Description.** MALE: (N = 2). Body length 2.16–2.46, length of scute 1.91–1.94, greatest width of anterior scute 1.29–1.32, greatest width of opisthosoma 1.92–1.98. Integument of body yellow-yellow/orange in color, faint to no pigment on anterior scute. OC height 0.08–0.12, width 0.22. OC low and rounded, wider than long in dorsal view. Pedipalpal coxae with 1 SBT at distal margin (lateral edge). CI with 7–8 spines. CII lobes with 1–2 apophyses. Both missing genital opercula. Chelicerae elongate. PCS greatest width 0.23–0.24, single dorsal SBT at distal edge, 2 small ventrolateral SBTs at distal margin. DCS length 0.75–0.82, greatest width 0.24–0.25. Pedipalps elongate. PF height 0.37–0.38, with dorsal row of 6–8 SBTs, 3–4 mesodorsal SBTs, 4–5 elongate medial spines, ventral row of 7–8 elongate spines; PT with 5 large medial spines, 5 large lateral spines. Legs extremely elongate. LI femur with ventral row of 4–5 SBTs, the third being smallest; tibia usually without ventral tubercles, but may have a single very small tubercle. LII total length 10.87–11.65: trochanter 0.42–0.43, femur 2.66–2.8, patella 0.77–0.79, tibia 2.37–2.44, metatarsus 2.2–2.56, tarsus 2.45–2.63. LII/SL 5.61–6.09.

**Material Examined.** UTAH: **Utah Co.:** North Madhouse Cave (GPS withheld), 27 April 2003, M. Porter, 1 male (SDSU: OP240); Professor Buss Cave (GPS withheld), 27 April 2003, M. Porter, 1 male (SDSU: OP239).

**Distribution.** Known only from North Madhouse and Professor Buss Caves near Provo, Utah.

**Genetic Data.** GenBank accession numbers: KJ585339, KJ585340, KJ585094, KJ585095, KJ585134, KJ585135, KJ585217, KJ585218, KJ585175, KJ585176, KJ585297, KJ585298, KJ585051, KJ585052, KJ585258, KJ585259.

***Sclerobunus ungulatus*** **(Briggs, 1971), comb. nov.**

Figures: **map** 3a; **penis** 7a; **habitus** 9b; **pedipalp** S8b; **leg I** S9b

*Cyptobunus ungulatus ungulatus*, Briggs 1971: 5, figs 10–18, map 1 [28]; Taylor et al. 2008: 314 [57]; Kury, 2003: 18 [54]

*Cyptobunus ungulatus*, Edgar, 1990: 540 [53]

**Type Material. Holotype** male, allotype female from Model Cave, near Baker, White Pine County, Nevada (GPS withheld), 24 August 1952, R. de Saussure (AMNH, not examined).

**Diagnosis.** *S. ungulatus* differentiated from *S. cavicolens* by a complete lack of pigment, lack of prongs on hind claws, pedipalpal tibia with 5 or more medial spines. Diagnosed from *S. madhousensis* by the longer length of LII (>13 mm).

**Description.** MALE: (N = 3). Body length 2.75–3.17, length of scute 2.15–2.17, greatest width of anterior scute 1.41–1.54, greatest width of opisthosoma 2.15–2.27. Integument of body yellow-yellow/orange in color, no pigment. OC height 0.08–0.09, width 0.27–0.29. OC low, rounded, wider than long in dorsal view. Pedipalpal coxae with 1 SBT at distal margin (lateral edge). CI with 8–9 spines. CII lobes with 1–2 apophyses. GO length 0.361, width 0.444 (most missing opercula). Chelicerae elongate. PCS greatest width 0.28, single dorsal SBT at distal edge, 2 small ventrolateral SBTs at distal margin. DCS length 0.91–0.94, greatest width 0.23–0.3. Pedipalps elongate. PF height 0.41–0.43, with dorsal row of 6 SBTs, 1–3 mesodorsal spines, 4–5 elongate medial spines, ventral row of 7–9 elongate spines; PT with 5–6 large medial spines, 5–6 large lateral spines. Legs extremely elongate. LI femur with ventral row of 3 SBTs, the third being smallest; tibia usually without ventral tubercles, but may have a single very small tubercle. LII total length 13.6–14.5: trochanter 0.52–0.54, femur 3.3–3.5, patella 0.91–0.94, tibia 2.9–3.1, metatarsus 3.0–3.3, tarsus 3.0–3.1. LII/SL 6.33–6.67.

**Material Examined.** NEVADA: **White Pine Co.:** Model Cave (GPS withheld), near Baker, 24 August 1952, R. de Saussure, 1 female, 1 adult (AMNH: A146, A147); Model Cave (GPS withheld), near Baker, total darkness, walls, 24 August 1952, R. de Saussure, 1 female, 1 adult (AMNH: A142); Model Cave (GPS withheld), near Baker, total darkness, wall and floor, 27 August 1952, R. de Saussure, 1 adult (AMNH: A133); Model Cave (GPS withheld), el. 2070 m, Great Basin National Park, on walls of cave, 25 June 2007, S. Derkarabetian, D. Elias, M. Hedin, L. Hedin, 3 males, 2 females (SDSU; GBPA:427 #8394–8398); Ice Cave (GPS withheld), el. 2070 m, Great Basin National Park, on walls of cave, 25 June 2007, S. Derkarabetian, D. Elias, M. Hedin, L. Hedin, 1 female, 2 juveniles (SDSU; GBPA:427 #8391–8393).

**Distribution.** Known from multiple caves in Great Basin National Park ([57], pg. 314).

**Genetic Data.** GenBank accession numbers: KJ585338, KJ585092, KJ585093, KJ585132, KJ585133, KJ585215, KJ585216, KJ585173, KJ585174, KJ585295, KJ585296, KJ585049, KJ585050, KJ585256, KJ585257.

***Sclerobunus nondimorphicus*** **group**

**Diagnosis.** The dorsal surface of the ventral plate of the penis is smooth, without pair of small ventral spines on lateral plates (FIGURE 7b).

**Included species.** *S. nondimorphicus* Briggs, *S. idahoensis* (Briggs).

**Distribution.** Pacific Northwest North America: Coast and Cascade ranges of northern Oregon, Washington, and southwestern British Columbia and the northern Rocky Mountains of Idaho and western Montana.

**Key to adults of** ***S. nondimorphicus*** **group**

1. Body with little to no black pigment (FIGURE 9d); OR, WA, BC… ***S. nondimorphicus***
  Body with much black pigment (FIGURE 9e); ID, MT… ***S. idahoensis*** **stat. nov.**

***Sclerobunus nondimorphicus*** **Briggs, 1971**

Figures: **map** 2; **habitus** 9d; **pedipalp** S8d; **leg I** S9d

*Sclerobunus robusta* [part], Banks, 1893: 152 [26]

*Sclerobunus robustus* [part], Banks 1901, 672 [48]; Banks, 1902: 593 [58]; Banks, 1911: 416 [59]; Roewer, 1923: 597, fig 746 [50]

*Sclerobunus nondimorphicus*, Briggs, 1971: 9, figs 42–53 [28]; Bragg and Leech, 1972: 70 [60]; Dumitrescu, 1976: 18, fig 14 [51]; Edgar, 1990: 540, fig 19.22 [53]; Kury, 2003: 18–19 [54]; Bragg and Holmberg, 2009: 30 [61]

**Type Material. Holotype** male and allotype female from 8.6 miles northwest of Easton on U.S. Highway 90, Kittitas County, Washington (N47.3101, W121.3148), collected on 23 June 1966 by T. Briggs, V. F. Lee, A. Jung, and K. Hom (CAS, not examined). **Paratypes:** 3 miles southeast of Rhododendron, near Mt. Hood, Clackamas County, Oregon (N45.3052, W121.87), 5 September 1976, T. Briggs, K. Hom, R. Lem, W. Lum, J. Nishio, 1 male (CAS, examined); 1 mile south Saddle Mountain State Park, Clatsop County, Oregon (N45.9639, W123.6885), under logs on ground, Sitka spruce forest biome, 7 August 1967, T. Briggs, A. Jung, 1 male (CAS, examined); 5.5 miles south Clatskanie, Columbia County, Oregon (46.0985, W123.2052), Sitka spruce log, 8 August 1967, K. Hom, 1 female, 1 juvenile (CAS, examined); 5.5 miles South Clatskanie, Columbia County, Oregon (46.0985, W123.2052), under surface of bark, Sitka spruce, 8 August 1967, K. Hom, 1 male, 2 females (CAS, examined); 5.5 miles south Clatskanie, Columbia County, Oregon (46.0985, W123.2052), bark and cut wood on ground, Sitka spruce forest, 8 August 1967, T. Briggs, 1 male (CAS, examined); 20.8 miles east of Queets on US 101, Grays harbor County, Washington (N47.4788, W123.9867), 22 June 1966, T. Briggs, V. Lee, A. Jung, 5 males, 2 females (CAS, examined); 6.8 miles south Neilton, Grays Harbor County, Washington (N47.3203, W123.9094), 22 June 1966, T. Briggs, V. Lee, A. Jung, K. Hom, 1 male (CAS, examined); 4.5 miles southwest Hoh Rainforest Road on US 101, Kings County, Washington, 22 June 1968, A. Jung, 1 male (CAS, examined); 16.4 miles northwest Hyak on US 90, Kings County, Washington (N47.4439, W121.6751), 23 June 1966, T. Briggs, V. Lee, A. Jung, K. Hom, 1 juvenile (CAS, examined); Ohanapecosh Campground, Mt. Rainier National Park, Lewis County, Washington (N46.7344, W121.5703), under surface of log, Douglas fir and cedar forest, 8 August 1967, T. Briggs, 1 male, 1 female (CAS, examined); 17.8 miles east of Hope, Manning Park, British Columbia (N49.2403, W121.1489), dense forest, 23 August 1969, T. Briggs, 3 males, 4 juveniles (CAS, examined).

**Diagnosis**. Compared to *S. idahoensis*, with much less black pigmentation on the scute and males do not have obvious swelling of the pedipalpal femur.

**Description.** MALES: (N = 7). Body length 2.84–3.16, length of scute 2.28–2.5, greatest width of anterior scute 1.58–1.68, greatest width of opisthosoma 2.31–2.59. Integument of body orange, with some black pigment, anterior scute with some pigment, lighter color directly behind OC. Anterior margin with 3–4 small tubercles (sometimes 1–2). OC height 0.17–0.19, width 0.38–0.41. OC variable, angled slightly forward, typically equal length and width in dorsal view, but can be wider, eyes connected with lighter black pigment. Pedipalpal coxae with a single SBT at distal margin. CI with 10 or more spines, some on tubercles. CII lobes generally with 3 apophyses, sometimes 2 or 4. GO length 0.41–0.47, width 0.47–0.52. Chelicerae lighter in color, without pigment. PCS greatest width 0.28–0.31, 1–2 small dorsal SBTs at distal edge, 1–2 small ventrolateral SBTs at distal edge. DCS length 0.87–0.91, greatest width 0.31–0.34. Pedipalps lighter in color, without pigment. PF height 0.52–0.6, with dorsal row of 5–7 SBTs, 2–3 mesodorsal spines, row of 3–4 medial spines, row of 6–7 ventral spines (rarely 5); PT with row of 4–5 medial spines, 5 lateral spines. Legs with black pigment on tibia, metatarsus, and tarsus. LI femur with row of 3–4 ventral SBTs; tibia with 1–2 ventral SBTs. LII total length 9.14–9.56: trochanter 0.47–0.54, femur 2.24–2.53, patella 0.76–0.83, tibia 1.93–2.03, metatarsus 2.14–2.38, tarsus 1.45–1.6. LII/SL 3.75–4.16.

FEMALES: (N = 4). Body length 2.92–3.66, scute length 2.36–2.53, greatest width of anterior scute 1.54–1.62, greatest width of opisthosoma 2.66–2.75. OC height 0.17–0.22, width 0.37–0.4. CII with 0–2 tubercles at posterior margin. GO length 0.42–0.47, width 0.55–0.56. PCS width 0.28–0.3. DCS length 0.85–0.88, greatest width 0.3–0.31. PF height 0.4–0.44. LII total length 8.25–9.16: trochanter 0.48–0.57, femur 2.03–2.2, patella 0.7–0.79, tibia 1.64–1.97, metatarsus 1.92–2.3, tarsus 1.33–1.4. LII/SL 3.41–3.66.

**Other Material Examined.** OREGON: **Benton Co.**: Mary's Peak Road, Mary's Peak, 0.1 miles west of FR 30 (N44.4964, W123.5457), el. 790 m, *Picea* forest, bark piles, 1 October 2010, S. Derkarabetian, M. McCormack, 2 females (SDSU); Mary's Peak Campground, Mary's Peak (N44.5087, W123.5582), el. 1070 m, *Picea* forest, bark piles, 1 October 2010, S. Derkarabetian, M. McCormack, 4 males, 1 female, 1 juvenile (SDSU). **Clackamas Co.**: 2.3 miles southeast of Rhododendron on FR 12 (N45.301, W121.8959), under bark and logs, 11 May 2011, S. Derkarabetian, A. Smith, 1 female (SDSU); Mermaloose Trail, 12 miles south of SR 224 along FR 45 (Mermaloose Creek Road) (N45.0986, W122.2219), el. 1100 m, mixed forest, woody debris, 16 August 2011, S. Derkarabetian, 1 male, 2 females (SDSU). **Clatsop Co.**: Lee Wooden Park, SR 202 4.3 miles north of SR 103 (N45.9576, W123.5815), el. 219 m, 16 June 2007, mixed forest, C. Richart, A. Fusek, 1 male (CHR1695), 1 female (CHR1696) (CHR). **Tillamook Co.**: Munson Creek Falls State Natural Area, 1.5 miles east of US 101 on Munson Creek Road (N45.3650, W123.7730), el. 100 m, mixed forest woody debris, 1 August 2011, S. Derkarabetian, 2 males, 1 juvenile (SDSU).

WASHINGTON: **Jefferson Co.**: Ruby Beach, Olympic National Park (N47.7098, W124.4137), el. 24 m, mixed forest, 2 July 2007, C. Richart, D. Richart, 2 males, 2 females, 1 juvenile (CHR). **King Co.**: Rattlesnake Lake, along Rattlesnake Ledge Trail, south of North Bend at US 90 (N47.4346, W 121.7722), el. 300 m, 30 December 2002, M. Hedin, C. Talbot, 1 female (SDSU). **Lewis Co.**: Rainbow Falls State Park, SR 6 16.1 miles west of US 15 (N46.6301, W123.233), streamside woody debris, C. Richart, 1 male (CHR); tributary of Iron Creek, FR 25 4.6 miles south of FR 300 (N46.4033, W121.9902), el. 6676 m, streamside woody debris, mixed forest, 6 August 2008, C. Richart, 1 male (CHR2483), 1 female (CHR). **Pacific Co.**: Ellsworth Creek Preserve TNC, along Ellsworth Creek (N46.4139, W123.8922), el. 69 m litter and woody debris, 2 April 2008, S. Derkarabetian, C. Richart, W. Leonard, 1 male (SDSU); along tributary of North Nemah River (N46.4916, W123.8242), el. 25 m, Berlese extraction of moss and woody debris, 20 September 2008, C. Richart 1 female (CHR).

**Distribution.** Coast and Cascade ranges of Oregon, Washington, and southern British Columbia.

**Genetic Data.** GenBank accession numbers: KJ585327–KJ585330, KJ585081–KJ585084, KJ585122–KJ585124, KJ585205–KJ585208, KJ585162–KJ585165, KJ585284–KJ585287, KJ585038–KJ585041, KJ585246–KJ585249, KJ585386, KJ585372, KJ585375, KJ585388, KJ585378, KJ585383, KJ585369, KJ585380.

***Sclerobunus idahoensis*** **(Briggs, 1971), stat. nov.**

Figures: **map** 2; **penis** 7b; **habitus** 9e; **pedipalp** S8e; **leg I** S9e

*Sclerobunus robustus idahoensis* Briggs, 1971: 11–12, figs. 61–66, map 1 [28]; Kury, 2003: 19 [54]

**Type Material. Holotype** male and allotype female from 2.8 miles northwest of Clarkia on State Highway 3, Shoshone County, Idaho (N47.0316, W116.2205), 11 August 1967, T. Briggs, K. Hom, A. Jung (CAS, not examined). **Paratypes:** 6.3 miles north of Headquarters, Clearwater County, Idaho (N46.7019, W115.8031), under surface of log, cedar, fir and spruce, 12 August 1967, T. Briggs, 1 male, 1 juvenile (CAS, examined); opposite Apgar Campground, Clearwater National Forest, Idaho County, Idaho (N46.214, W115.5374), under surface of log, cedar, fir and spruce, 12 August 1967, T. Briggs, 1 male, 1 female (CAS, examined); 17.25 miles southwest of Little North Fork of Clearwater River on Clearwater Road, Shoshone County, Idaho, 11 August 1967, T. Briggs, K. Hom, A. Jung, 1 male, 2 female, 4 juveniles (CAS, examined).

**Diagnosis.** Distinguished from *S. nondimorphicus* by the presence of more black pigment.

**Description.** MALE: (N = 15). Body length 2.69–3.24, scute length 2.16–2.55, greatest width of anterior scute 1.44–1.73, greatest width of opisthosoma 2.24–2.73. Integument of body deep orange in color, scute with heavy black pigment, sometimes solid, extremities lighter in color. Anterior margin of scute with 3–5 tubercles. OC height 0.14–0.26, width 0.34–0.43. OC rounded to somewhat pointed, angled forward slightly, equal length and width in dorsal view, eyes connected with black pigment, anterior scute with patterned pigment. Pedipalpal coxae with single large SBT at distal margin (lateral edge). CI with 10 or more spines (up to 18), some on tubercles. CII lobes with either 2 or 3 apophyses, the third being smallest, if present. GO length 0.41–0.48, width 0.44–0.58. PCS greatest width 0.22–0.32, 2 (rarely a single) small dorsal SBTs at distal edge, 2 (rarely a single) small ventrolateral SBTs at distal edge. DCS length 0.79–0.93, greatest width 0.29–0.34. Pedipalps typically without black pigment. PF height 0.47–0.58, with dorsal row of 5–7 SBTs (sometimes 4), 2–4 mesodorsal spines, 3–4 medially, ventral row of 7–8 spines (sometimes 5 or 9); PT with row of 4–5 medial spines (sometimes 6), 5 lateral spines (rarely 6). Legs with black pigment on the tibia, metatarsus, and tarsus. LI femur with row of 3–5 ventral SBTs; tibia with 1–2 ventral SBTs. LII total length 7.82–9.58: trochanter 0.45–0.52, femur 1.91–2.35, patella 0.71–0.87, tibia 1.6–2.04, metatarsus 1.8–2.4, tarsus 1.26–1.5. LII/SL 3.23–4.17.

FEMALES: (N = 11). Body length 2.8–3.68, scute length 2.3–2.69, greatest width of anterior scute 1.41–1.69, greatest width of opisthosoma 2.4–2.83. OC height 0.16–0.23, width 0.36–0.41. CII and CIII can have fewer tubercles. GO length 0.37–0.46, width 0.46–0.53. PCS greatest width 0.26–0.32. DCS length 0.8–0.91, greatest width 0.28–0.32. PF height 0.39–0.45. LII total length 7.09–9.0: trochanter 0.44–0.52, femur 1.71–2.2, patella 0.66–0.82, tibia 1.48–1.9, metatarsus 1.64–2.2, tarsus 1.12–1.42. LII/SL 2.86–3.74.

**Other Material Examined.** IDAHO: **Clearwater Co.**: Rhodes Creek, 2.0 miles southeast of SR 11 on FSR 250 (N46.4767, W115.7809), el. 960 m, *Abies grandis* forest litter, 19 July 2008, C. Richart, 10 males, 2 females (CHR). **Idaho Co.**: tributary of Crooked Creek, 14.1 miles south of Red River Road on FSR 222 (N45.5791, W115.4431), el. 1870 m, *Abies* forest, leaf litter, 7 July 2008, S. Derkarabetian, C. Richart, J. Underwood, 3 males, 3 females (CHR); Grouse Creek, 1.9 miles southwest of Hungary Ridge Road on FSR 1299 (N45.812, W115.953), el. 1005 m, *Abies* forest, leaf litter, 6 July 2008, S. Derkarabetian, C. Richart, J. Underwood, 7 males, 3 females (CHR); 0.8 miles south of Selway River Road on FSR 443 (N46.0385, W115.2943), el. 535 m, conifer forest, forest litter and streamside woody debris, 6 July 2008, S. Derkarabetian, C. Richart, J. Underwood, 4 males, 2 females, 2 juveniles (CHR); DeVoto Memorial Cedar Grove (N46.4293, W115.1335), el. 1100 m, old growth *Thuja plicata* forest, leaf litter, 5 July 2008, S. Derkarabetian, C. Richart, J. Underwood, 9 males, 3 females, 2 juveniles (CHR). **Kootenai Co.**: Rose Lake, 1.5 miles north of US 23 on South Rose Creek Road (N47.5531, W116.4967), el. 660 m, *Tsuga heterophylla* and *Abies grandis* forest, woody debris, 23 July 2011, S. Derkarabetian, C. Richart, 8 males, 5 females, 1 juvenile (SDSU). **Shoshone Co.**: Hobo Cedar Grove Botanical Area, St. Joe National Forest (N47.0860, W116.1129), el. 1250 m, old growth *Thuja plicata*, *Taxus brevifolia*, along creeks/seeps, 25 July 2011, S. Derkarabetian, C. Richart, 2 male, 3 female, 1 juvenile (SDSU); Placer Creek Road, 4.6 miles southeast of High Street (N47.4303, W115.8913), el. 1110 m, *Tsuga heterophylla*, *Thuja plicata*, *Abies grandis* forest litter, 29 July 2008, C. Richart, 3 males, 2 females (CHR).

MONTANA: **Mineral Co.**: 9.3 miles south of Frontage Road of I-90 on FSR 320 (N47.1102, W115.0095), el. 1156 m, *Pseudotsuga menziesii*, *Thuja plicata* forest, streamside woody debris, 10 July 2012, C. Richart, 2 males (SDSU); Goose Creek, 9.7 miles south of Mullan Gulch Road on FSR 282 (N47.2279, W115.2464), el. 1305 m, *Thuja plicata*, *Abies grandis*, *Picea* forest litter, 30 July 2008, C. Richart, 1 male, 3 females, 1 juvenile (CHR); Deep Creek, 9.7 miles southwest of Diamond Match Road on Trout Creek Road (N47.0464, W114.9503), el. 1106 m, *Pseudotsuga menziesii*, *Thuja plicata* forest, streamside woody debris, 30 July 2008, C. Richart, 1 female (SDSU); 9 miles west of St. Regis (N47.332, W115.2625), 10 August 1929, 1 male (AMNH). **Missoula Co.**: Spring Gulch, 0.3 kilometers northwest of US 12 (N46.7378, W114.5351), el. 1279 m, decomposing log, 18 June 2009, P. Marek, 2 males, 1 female (SDSU).

**Distribution.** Mountainous regions of northern Idaho (northern Rocky Mountains north of the Salmon River) and extreme western Montana (Bitterroot Range).

**Genetic Data.** GenBank accession numbers: KJ585331–KJ585334, KJ585085–KJ585088, KJ585125–KJ585128, KJ585209–KJ585211, KJ585166–KJ585169, KJ585288–KJ585291, KJ585042–KJ585045, KJ585250–KJ585252.

**Comments.** Both *S. nondimorphicus* and *S. idahoensis* were delimited as separate species. Differences exist in somatic morphology, morphometric analyses largely supports two distinct clusters corresponding to the two species (FIGURE 4), and coalescent-based analyses support the presence of a species tree node (FIGURE 5). Despite this, these two species are non-monophyletic in all gene trees (FILE S6) and *gsi* statistics do not support monophyly for any genes (TABLE 2). Multispecies coalescent models, like that implemented in BPP, may still support species status despite the non-monophyly of gene trees, as incongruence among gene trees is attributed to deep coalescence [62], [63]. Previous analyses based on mitochondrial COI support the reciprocal monophyly of these two species [22] providing additional evidence for distinct species and the deep coalescence of nuclear genes. Biogeography also supports species status as these species occupy regions separated by a well-known biogeographical break seen in numerous species [64]. This situation requires further study with denser geographic and genetic sampling, especially to determine if more fine-scale geographical patterns exist within either *S. nondimorphicus* or *S. idahoensis*.

***Sclerobunus robustus*** **group**

**Diagnosis.** The dorsal surface of the ventral plate of the penis has many folds, with a pair of small ventral spines on lateral plates (FIGURES 7c and 8).

**Included species.** *S. robustus* Briggs, *S. glorietus* (Briggs), *S. jemez* sp. nov., *S. klomax* sp. nov., *S. skywalkeri* sp. nov., *S. speoventus* sp. nov., and *S. steinmanni* sp. nov.

**Distribution.** Mountainous regions of Arizona, New Mexico, Colorado, and southeastern Utah, including caves.

**Key to adults of** ***S. robustus*** **species group**

1. OC not angled forward (FILE S7a)… **2**
  OC angled forward to some degree (FILE S7b,c)… **3**
2. Body with very little, if any, black pigment (FIGURE 10a); LI tibia with one or two ventral SBTs (FILE S9f); subapical spines of penis strongly curved, ventral plate normal (FIGURE 8a); Cave of the Winds, CO… ***S. speoventus*** **sp. nov.**
  Body and legs with considerable faint black pigment (FIGURE 10b); LI tibia with three or more ventral SBTs (FILE S9g); subapical spines of penis normal, ventral plate dorsoventrally compressed (FIGURE 8b); Mallory Cave, CO… ***S. steinmanni*** **sp. nov.**
3. LII length over 8 mm… **4**
  LII length under 8 mm… **5**
4. Pedipalpal tibia with 4 prolateral and 5 retrolateral SBTs (FILE S8j); 6–7 SBTs on CI; LI femur with 3 ventral SBTs (FILE S9j); northern NM… ***S. klomax*** **sp. nov.**
  Pedipalpal tibia with 5 prolateral and 5–7 retrolateral SBTs (FILE S8k); 10 or more SBTs on CI; LI femur with 4–6 ventral SBTs (FILE S9k); Terrero Cave, NM… **S.** ***jemez*** **sp. nov.**
5. Scute length usually under 1.8 mm; male PF height under 0.5 mm; northern NM… ***S. glorietus*** **stat. nov.**
  Scute length usually over 1.8 mm; male PF height over 0.5 mm… **6**
6. Male LII length generally over 7 mm; scute length generally over 2.2 mm; OC apex rounded in profile (FILE S7b); AZ, NM, CO, UT… ***S. robustus***
  Male LII length generally under 6.2 mm; scute length generally under 2.2 mm; some populations with OC apex pointed in profile (FILE S7c); central NM… ***S. skywalkeri*** **sp. nov.**

***Sclerobunus robustus*** **(Packard, 1877)**

Figures: **map** 3b; **penis** 7c; **habitus** 10c, S7b,e,f; **pedipalp** S8h; **leg I** S9h

*Scotolemon robustum*, Packard, 1877: 164, fig 8 [25]

*Phalangodes robusta*, Simon, 1879a: 185 [65]; Simon, 1879b: 156 [66]

*Phalangodes robustus*, Packard, 1888: 48, fig 13 [47]

*Sclerobunus robusta* [part], Banks, 1893: 152 [26]

*Sclerobunus robustus*, [part] Banks, 1894: 431 [67]; Banks, 1901, 672 [48]; Banks, 1902: 593 [58]; Banks, 1911: 416 [59]; Roewer, 1915: 87, fig 13 [31]; Roewer, 1923: 597, fig 746 [50]; Ekpa et al., 1984 [68]

*Sclerobunus robustus robustus*, Briggs, 1971: 10–11, figs 54–60, map 1 [28]; Cokendolpher et al., 1993 [69]; Kury, 2003: 19 [54]

**Type Material. Paratypes:** 1 specimen, West Cliff, Colorado (MCZ: Cat # 39045, not examined); 4 specimens, West Cliff, Colorado (MCZ: Cat # 39048, not examined). **Syntype**s: 2 specimens, Colorado (MCZ: Cat # 39044, not examined).

**Diagnosis.** Diagnosed from all other surface species in the *S. robustus* group by its larger body size (scute length generally >2.2 mm) and longer legs (LII generally >7 mm). Some populations of *S. robustus* are very similar to *S. skywalkeri* in morphological characteristics (i.e., Bradford Canyon), but can be distinguished based on geographical distribution. Differentiated from cave-adapted species of the *S. robustus* group by its lack of troglomorphic features. Several populations of cave-inhabiting *S. robustus* are known, however they can be diagnosed from all troglomorphic species of the *S. robustus* group based on LII length (<8 mm in *S. robustus*, >8 mm in troglomorphic species).

**Description.** MALE: (N = 13). Body length 2.44–3.18, length of scute 1.91–2.63, greatest width of anterior scute 1.28–1.66, greatest width of opisthosoma 1.89–2.72. Integument of body orange in color, lighter in cave populations, presence of black pigment ranges from faint (Northeast clade) to much (Southeast clade), anterior scute may have pigment (Southeast and Southwest clades). Anterior margin with 2–4 tubercles. OC height 0.12–0.18, width 0.25–0.37. OC rounded, angled forward. Pedipalpal coxae usually with two SBTs at distal margin, one larger, some with only a single larger tubercle (Haviland clade). CI with 10 or more spines, some on tubercles. CII with 1–4 distal posterior tubercles, 5–6 in some (Northeast clade). CII lobes with 2–3 apophyses. GO length 0.31–0.39, width 0.37–0.43. Chelicerae lighter in color, usually without black pigment, some with pigment (Southeast and Southwest clades). PCS greatest width 0.22–0.32, a single small dorsal SBT at distal edge, 1–2 small ventrolateral SBTs at distal edge, some with 3–4 (Southwest clade). DCS length 0.7–0.98, width 0.36–0.35. Pedipalps lighter in color, some with pigment (Central eastern, Southeastern, and Southwestern clades). PF height 0.53–0.82, with dorsal row of 6–8 SBTs, 9–11 in some (Southwest clade), 3–4 medial spines, row of 6–8 ventrally, most with small spine inserted within the first 3 larger spines; PT with row of 4–5 large medial spines, 5–6 lateral spines, some with 3–4 (Southeast clade). Legs lighter in color, generally with black pigment on femur, patella, tibia, metatarsus, tarsus. LI femur generally with row of 3 ventral SBTs; tibia with 1 or 2 ventral SBTs. LII total length 5.63–7.87: trochanter 0.37–0.52, femur 1.31–2.14, patella 0.56–0.75, tibia 1.18–1.79, metatarsus 1.28–2.09, tarsus 0.93–1.33. LII/SL 2.86–3.9.

**Variation.** Considerable variation exists within this species. Clades noted in parentheses above are those named in Derkarabetian et al. [22].

**Material Examined.** ARIZONA: **Pima Co.**: Santa Catalina Mountains, vic. Sunset Trailhead (N32.4265, W110.7424), el. 2377 m, mixed conifer forest, north-facing slope, 15 July 2006, J. Deas, S. Derkarabetian, M. Hedin, S. Thomas, 4 males, 8 females, 3 juveniles (SDSU).

COLORADO: **Custer Co.**: HWY 165, south of McKenzie junction, Wet Mountains (N38.1336, W105.1791), el. 2710 m, mixed aspen/conifer forest, above small stream, 2 July 2007, S. Derkarabetian, D. Elias, M. Hedin, 7 males, 6 females (SDSU). **Garfield Co.**: Hanging Lake Park, Hanging Lake Trail, along Dead Horse Creek (N39.5985, W107.191), el. 2118 m, under woody debris, mixed forest, 1 August 2009, S. Derkarabetian, M. McCormack, 2 males (SDSU). **Gilpin Co.**: Apex Valley Road, junction with HWY 119, 1.7 miles north of Black Hawk on 119 (N39.8192, W105.5132), el. 2580 m, north-facing slope, pine forest, 31 July 2009, S. Derkarabetian, M. McCormack, 2 males (SDSU). **La Plata Co.**: vic. Haviland Lake Campground, off HWY 550, north of Durango (N37.5329, W107.807), el. ∼2440 m, north-facing hillside, mixed conifer, 28 June 2007, S. Derkarabetian, D. Elias, M. Hedin, 11 males, 5 females, 12 juveniles (SDSU). **Rio Grande Co.**: Church Creek Trailhead, southwest of South Fork, off HWY 160 (N37.6481, W106.652), el. 2530 m, mixed aspen/conifer ravine, 28 June 2007, S. Derkarabetian, D. Elias, M. Hedin, 27 males, 9 females (SDSU).

NEW MEXICO: **Otero Co.**: HWY 244, southwest of Silver Springs in Bradford Canyon, northeast of Cloudcroft (N32.978, W105.7087), el. ∼2650 m, Douglas fir/aspen forest on shallow north-facing slope, 19 July 2006, J. Deas, S. Derkarabetian, M. Hedin, S. Thomas, 1 male, 3 females (SDSU). **Sandoval Co.**: Jemez Mountains, HWY 4, 3.9 miles west of junction with HWY 501 at Los Alamos (N35.8384, W106.4044), el. ∼2743 m, mixed aspen/spruce/pine flats, J. Deas, S. Derkarabetian, M. Hedin, S. Thomas, 14 males, 7 females.

UTAH: **San Juan Co.**: La Sal Mountains, La Sal Pass Road (N38.4155, W109.2242), el. ∼2900 m, aspen grove, 27 June 2007, S. Derkarabetian, D. Elias, M. Hedin, 9 males, 8 females (SDSU).

**Distribution.** Known from Arizona, Colorado, New Mexico, and southeastern Utah.

**Genetic Data.** GenBank accession numbers: KJ585356–KJ585367, KJ585112–KJ585121, KJ585150–KJ585161, KJ585235–KJ585245, KJ585193–KJ585204, KJ585315–KJ585326, KJ585069–KJ585080, KJ585274–KJ585283, KJ585387, KJ585373, KJ585376, KJ585389, KJ585379, KJ585384, KJ585370, KJ585381.

***Sclerobunus glorietus*** **(Briggs, 1971), stat. nov.**

Figures: **map** 3b; **habitus** 10d, S7d; **pedipalp** S8i; **leg I** S9i

*Sclerobunus robustus glorietus*, Briggs, 1971: 12, figs 67–72, map 1 [28]; Kury, 2003: 19 [54]

**Type Material. Holotype** male and allotype female from 4 miles southeast of Glorieta Baldy Lookout, Santa Fe County, New Mexico (N35.608, W105.769), collected on 14 August 1968 by T. Briggs, K. Hom, and D. Owyang (CAS, not examined). **Topotypes** (10 males, 1 female) with same locality information as holotype (CAS, examined).

**Diagnosis.** This is the smallest species of *Sclerobunus* and is diagnosed from other *S. robustus* group members based on body size (male scute length <1.8 mm). The lesser degree of black pigmentation on the body can help diagnose this taxon from smaller specimens of *S. skywalkeri*.

**Description.** MALE: (N = 5). Body length 1.91–2.17, length of scute 1.69–1.83, greatest width of anterior scute 1.06–1.15, greatest width of opisthosoma 1.69–1.82. Integument of body orange, with some black pigment, anterior scute with lighter, patterned pigment, without pigment behind OC. Anterior margin with 3–4 tubercles. OC height 0.09–0.13, width 0.22–0.27. OC tall, rounded, angled forward. Pedipalpal coxae with 2 SBTs at distal margin. CI with 8 spines, some on tubercles. CII with 7 posterior distal tubercles. CII lobes with 2 apophyses. GO length 0.27, width 0.33. Chelicerae lighter in color. PCS width 0.21–0.22, 1 small dorsal SBT at distal edge, 1–2 small ventrolateral SBTs at distal edge. DCS length 0.6–0.65, greatest width 0.23–0.24. PF height 0.37–0.43, with dorsal row of 6–8 tubercles, row of 6–7 ventral spines; PT with row of 4 medial spines, row of 4–5 lateral spines. Legs with light black pigment on femur, patella, tibia, metatarsus, tarsus. LI femur with row of 3 ventral SBTs; tibia with 1–2 ventral SBTs. LII total length 4.7–5.1: trochanter 0.31–0.34, femur 1.08–1.13, patella 0.47–0.5, tibia 0.96–1.06, metatarsus 0.99–1.12, tarsus 0.78–0.93. LII/SL 2.71–2.83.

FEMALE: (N = 3). Body length 2.13–2.36, scute length 1.74–1.8, greatest width of anterior scute 1.01–1.03, greatest width of opisthosoma 1.72–1.88. OC height 0.09–0.12, width 0.22–0.24. GO 0.23–0.27, width 0.33–0.37. PCS width 0.2–0.21. DCS length 0.58–0.59, greatest width 0.22–0.23. PF height 0.28–0.29. LII total length 4.45–4.78: trochanter 0.3–0.33, femur 1.04–1.16, patella 0.44–0.46, tibia 0.9–0.98, metatarsus 0.94–0.96, tarsus 0.81–0.9. LII/SL 2.56–2.66.

**Material Examined.** NEW MEXICO: **Santa Fe Co.**: north of Glorieta Baptist Camp, Glorieta Canyon (N35.6118, W105.7703), in north-facing Douglas fir thicket, el. 2347 m, 20 July 2006, S. Derkarabetian, J. Deas, M. Hedin, S. Thomas, 2 males, 2 females (SDSU: OP890–892); north of Aspen Ranch on Rio Nambe (N35.8281, W105.8251), el. 2500 m, 30 April 1978, D.C. Lowrie (AMNH); near ski area northeast of Santa Fe (N35.7883, W105.7959), C.C. Hoff, 3 females (AMNH); Santa Fe Baldy near Santa Fe (N35.8329, W105.7586), C.C. Hoff, 1 male (AMNH). **Taos Co.**: Taos Ski Valley road (HWY 150, northeast of Arroyo Seco), 7.7 miles from junction of SR 150 and SR 230, forested talus slope, along SR 150 (N35.6118, W105.7703), 20 July 2006 by J. Deas, S. Derkarabetian, M. Hedin, S. Thomas, 2 males, 2 females (SDSU: OP881–883); same collecting locality as previous, 3 July 2007, S. Derkarabetian, D. Elias, M. Hedin, L. Hedin, 1 male, 3 females (SDSU: OP1169–1171); same collecting locality as previous, 4 June 2008, S. Derkarabetian, R. Fawcett, 1 male, 1 female, I juvenile (SDSU: OP2119); same collecting locality as previous, 30 July 2009, S. Derkarabetian, M. McCormack, 1 female (SDSU).

**Distribution.** Known from the southern Sangre de Cristo Mountain Range in New Mexico.

**Genetic Data.** GenBank accession numbers: KJ585351–KJ585353, KJ585107–KJ585109, KJ585146–KJ585147, KJ585230–KJ585232, KJ585188–KJ585190, KJ585310–KJ585312, KJ585064–KJ585066, KJ585269–KJ585271.

**Comments.** In prior analyses [21] the *S. glorietus* complex only included 4 samples with only one highly supported branch, a sister relationship between *S. klomax* and the *S. glorietus* population from Taos Ski Valley. In previous COI-only analyses [22], *S. skywalkeri* is monophyletic and highly supported, *S. klomax* and the *S. glorietus* from Taos Ski Valley were reciprocally monophyletic with low support, and *S. glorietus* from Glorieta Canyon was recovered sister to the entire *S. robustus* species group. Similar difficulties occurred in the present study in which morphological clusters were not supported by gene trees. In most gene trees, the Glorieta Canyon population of *S. glorietus* was recovered either within or sister to *S. skywalkeri* populations, however, support for these relationships was low (FILE S6). Despite this uncertainty, and given that BPP analyses are sensitive to guide tree topology [8], we argue that defining three species within the *S. glorietus* complex based on the morphometric clustering and qualitative morphological similarity is the most conservative and intuitive choice and unlikely to result in future synonymy with increased sampling. Further sampling focused on the geographic gap between the two populations of *S. glorietus* may eliminate the discrepancy between the morphological and genetic data seen here.

***Sclerobunus jemez*** **sp. nov.**

urn:lsid:zoobank.org:act:748E456F-F63E-47E5-889C-5E2331557EA2

Figures: **map** 3b; **habitus** 10f; **pedipalp** S8k; **leg I** S9k

**Type Material. Holotype** male from Terrero Cave, Santa Fe County, New Mexico (GPS withheld), collected on 18 June 1975 by W. C. Welbourn (CAS, examined). **Paratypes:** 1 male, 2 females same collecting data as holotype (CAS, examined). Holotype and paratypes deposited at CAS.

**Etymology**. The specific epithet is a noun in apposition named in honor of the Jemez Pueblo for whom Terrero Cave is culturally and spiritually significant.

**Diagnosis**. Diagnosed from all other species in the *S. robustus* group by a combination of scute length of 1.9–2.0 mm and LII length between 8.0–9.0 mm. *S. jemez* is generally less troglomorphic than *S. steinmanni*, *S. speoventus* and *S. klomax*. The first three proximal ventral spines of the PF are stouter and shorter than other troglomorphic species. *S. jemez* has more spines on CI (8–12) compared to *S. klomax* (6–7).

**Description**. MALE: Holotype (1 paratype). Body length 2.11 (2.18), length of scute 2.0 (1.93), greatest width of anterior scute 1.25 (1.23), greatest width of opisthosoma 1.88 (1.96). Integument of body yellowish in color, anterior scute slightly darker, no pigment present. Anterior margin of scute with 2–3 tubercles. OC height 0.94(1.0), width 0.23 (0.23). OC tall, rounded, anteriorly directed, as wide as long. Pedipalpal coxae with a single SBT at distal margin (lateral edge). CI with 10 spines, some on large tubercles. CII with 4–5 distal posterior tubercles. CII with 2 small, thin apophyses (left apophysis damaged on holotype, paratype with 3). GO length 0.29 (0.3), width 0.32 (0.33). Chelicerae elongate. PCS greatest width 0.21 (0.23), single small spine at anterior dorsal margin, 2 small ventrolateral SBTs at distal margin. DCS length 0.75 (0.74), width 0.24 (0.25). Pedipalps elongate. PF height 0.42 (0.41), with dorsal row of 7–8 small SBTs, 4 small mesodorsal SBTs, 3 elongate medial spines, row of 8 elongate ventral spines; PT with row of 5 large medial spines, 6–7 large lateral spines. Legs elongate. LI trochanter with several ventral setae, one on small tubercle; femur with ventral row of 5–6 SBTs, the second being smallest; tibia with ventral row of larger 2–3 SBTs. LII total length 9.09 (8.78): trochanter 0.4 (0.41), femur 2.04 (2.08), patella 0.71 (0.69), tibia 1.87 (1.72), metatarsus 1.96 (1.86), tarsus 2.11 (2.02). LII/SL 4.55 (4.55).

FEMALE: 2 paratypes. Body length 2.16–2.3, scute length 1.94–1.97, greatest width of anterior scute 1.23, greatest width of opisthosoma 1.94. OC height 0.09–0.11, width 0.21–0.22. GO length 0.31, width 0.36–0.38. PCS width 0.22–0.23. DCS length 0.71–0.88, width 0.24. PF height 0.32–0.33. LII total length 8.08–8.34: trochanter 0.4–0.42, femur 1.96–2.04, patella 0.62–0.64, tibia 1.62–1.64, metatarsus 1.67–1.73, tarsus 1.79–1.93. LII/SL 4.1–4.32.

**Distribution**. Known only from type locality.

***Sclerobunus klomax*** **sp. nov.**

urn:lsid:zoobank.org:act:2156504D-0E52-4AD5-8DDD-56A45CB1ED24

Figures: **map** 3b; **habitus** 10e; **pedipalp** S8j; **leg I** S9j

**Type Material. Holotype** female, from rock pile on forested talus slope, el. 2865 m, along SR 150, 7.7 miles from junction of SR 150 and SR 230, vic. Taos Ski Valley, Taos County, New Mexico (N35.6118, W105.7703), collected on 3 July 2007 by S. Derkarabetian, D. Elias, M. Hedin, L. Hedin, (deposited at CAS; SDSU: OP1171). GenBank accession numbers: KJ585355, KJ585111, KJ585149, KJ585234, KJ585192, KJ585314, KJ585068, KJ585273. **Paratypes:** 1 female, same collecting data as holotype, collected on 20 July 2006 by J. Deas, S. Derkarabetian, M. Hedin, S. Thomas (SDSU DNA voucher OP972). GenBank accession numbers: KJ585354, KJ585110, KJ585148, KJ585233, KJ585191, KJ585313, KJ585067, KJ585272; 1 female, same collecting data as holotype, collected on 30 July 2009 by S. Derkarabetian, M. McCormack (SDSU).

**Etymology.** The specific epithet is a noun in apposition from the Greek word *klomax*, meaning “heap of stones, stony place”. This refers to the known habitat of this species, from montane rock piles.

**Diagnosis.** Differentiated from all surface *Sclerobunus* species and its nearest relative, (syntopic) *S. glorietus*, by its troglomorphic features. Differs from all currently known troglomorphic *Sclerobunus* by a combination of a low, anteriorly directed OC, near absence of black pigmentation, smaller body size, and fewer setae on CI.

**Description.** FEMALE: Holotype (2 paratypes). Body length 2.12 (1.95–2.18), length of scute 1.85 (1.63–1.82), greatest width of anterior scute, 1.17 (1.08–1.16), greatest width of opisthosoma 1.94 (1.72–1.95). Integument of body uniformly orange/yellow in color, anterior scute with very little faint pigment, scute margins slightly darker. Anterior margin of scute with 3 tubercles. OC height 0.08 (0.09–0.1), width 0.19 (0.2). OC low, anteriorly directed, triangular in profile, roughly as long as wide, black pigment connecting eyes. Pedipalpal coxae with 1 SBT at distal margin. CI with 6–7 setae, some on tubercles. GO length 0.29 (0.21), width 0.36 (0.32–0.34). Chelicerae elongate. PCS greatest width 0.22 (0.2–0.21), with a single distal mesodorsal seta, single distolateral seta. DCS length 0.72 (0.67–0.7), width 0.23 (0.22). Pedipalps elongate. PF height 0.33 (0.29–0.32), with dorsal row of 5–6 small SBTs, 2–3 small mesodorsal spines, a row of 3 elongate medial spines, ventral row of 8–9 elongate spines; patella with a single distolateral tubercle with seta at anterior margin; PT with row of 4 large medial spines, 5 lateral spines. Legs elongate. LI femur with ventral row of three SBTs, the third being smallest; tibia with ventral row of larger setae, two arising from tubercles. Holotype female missing both LII past femur. LII total length ? (8.03–8.63): trochanter 0.41 (0.38–0.41), femur 2.09 (1.93–2.06), patella ? (0.57–0.63), tibia ? (1.55–1.77), metatarsus ? (1.57–1.67), tarsus ? (2.04–2.09). LII/SL ? (4.74–4.93). Lateral prongs of hind tarsal claws somewhat reduced.

MALE: Unknown.

**Variation.** There is some variation in the spination counts on the PF.

**Natural History.** All specimens of this species were collected from beneath rock piles in a small area of forested, south-facing talus slope. Only three specimens have been found, despite four separate visits over four years to the type locality. It is possible that specimens inhabit deeper layers of the talus slope (superficial subterranean habitats) and might have a broader regional distribution in similar microhabitats.

**Distribution.** Known only from the type locality.

**Remarks.** In *Sclerobunus*, there is only a single case of sympatry: at Taos Ski Valley where the highly troglomorphic *S. klomax* can be found syntopic with a population of *S. glorietus*. This location has been discussed in detail elsewhere [21], however we reiterate that the troglomorphic *S. klomax* has only been collected from beneath rock piles, while *S. glorietus* are found underneath logs and rocks resting on the surface. This sympatry with an absence of known hybrids additionally supports the status of two separate species.

***Sclerobunus skywalkeri*** **sp. nov.**

urn:lsid:zoobank.org:act:28C72714-D99A-44FD-9F1D-5EE3FBBB7113

Figures: **map** 3b; **habitus** 10g, S7c; **pedipalp** S8l; **leg I** S9l

**Type Material. Holotype** male, road to Capilla Peak in Canon Huevo, Manzano Mountains, Cibola NF, Torrance County, New Mexico (N34.6658, W106.3907), 19 July 2006, J. Deas, S. Derkarabetian, M. Hedin, S. Thomas (deposited at CAS). **Paratypes:** 1 male, 1 female, same collecting locality information as holotype (deposited at CAS); 4 males, 1 female, same locality information as holotype (SDSU: OP907–911). OP908 GenBank accession numbers: KJ585348, KJ585104, KJ585143, KJ585227, KJ585185, KJ585307, KJ585061, KJ585267; 1 male, 5 females, FR 193, 4 miles east of HWY 547 in Lobo Canyon, northeast of Grants, Mt. Taylor, Cibola Co., New Mexico (N35.2318, W107.6388), 21 July 2006, J. Deas, S. Derkarabetian, M. Hedin, S. Thomas (deposited at CAS); 1 male, 1 female, 2 juveniles, FR 193, 4 miles east of HWY 547 in Lobo Canyon, northeast of Grants, Mt. Taylor, Cibola Co., New Mexico (N35.2318, W107.6388), 21 July 2006, J. Deas, S. Derkarabetian, M. Hedin, S. Thomas (SDSU: OP901–904). OP903 GenBank accession numbers: KJ585350, KJ585106, KJ585145, KJ585229, KJ585187, KJ585309, KJ585063; 3 males, 10.2 miles from SR 14 along Sandi Crest Road, north-facing mixed forest, Sandia Mountains, Bernalillo County, New Mexico (N35.2099, W106.4308), 3 June 2008, S. Derkarabetian, R. Fawcett (SDSU: OP2101–2103). OP2101 GenBank accession numbers: KJ585349, KJ585105, KJ585144, KJ585228, KJ585186, KJ585308, KJ585062, KJ585268.

**Etymology.** The specific epithet is in reference to the character Luke Skywalker and is used to recognize the multi-generational influence and significance of the original Star Wars trilogy. This name also reflects the fact that this species is found only at high elevations in “sky island” montane habitats, and thus appears to “walk the sky”.

**Diagnosis.** Differentiated from *S. glorietus* by greater PF height (>0.5 mm) and larger body size (scute length >1.8 mm) and differentiated from *S. robustus* by generally smaller body size (scute length <2.2 mm) and shorter legs (<7 mm). Some populations of *S. robustus* (i.e., Bradford Canyon) are very similar to *S. skywalkeri* in morphological characteristics, but can be differentiated based on geographical distribution. Differentiated from the cave-dwelling species of *S. robustus* group based on lack of troglomorphic characters.

**Description.** MALE: Holotype (5 paratypes). Body length 2.4 (2.28–2.51), scute length 2.05 (1.91 2.19), greatest width of anterior scute 1.27 (1.23–1.32), greatest width of opisthosoma 1.98 (1.92–2.1). Integument of body deep orange-orange/red, with much black pigment, anterior scute with patterned black pigment. Anterior margin of scute with 3 tubercles. OC height 0.16 (0.1–0.2), width 0.28 (0.26–0.29). OC tall, rounded, angled forward, eyes connected with black pigment. Pedipalpal coxae with 2 SBTs at distal margin. CI with 8 spines, some on tubercles. CII with 5 distal posterior tubercles. With 3 apophyses at CII complex. GO length 0.29 (0.29–0.31), width 0.35 (0.36–0.39). Cheliceral integument lighter, with some black pigment. PCS width 0.23 (0.23–0.27), single dorsal SBT at distal edge, single ventrolateral seta at distal margin. DCS length 0.74 (0.63–0.73), greatest width 0.26 (0.24–0.27). Pedipalpal integument lighter, with some black pigment. PF height 0.54 (0.5–0.59), with dorsal row of 6 small SBTs, 5 small mesodorsal tubercles, row of 3 medial spines, ventral row of 7 spines, with small spines between second and third spines; PT with row of 4 large medial spines, row of 5 lateral spines. All leg segments with black pigment. LI femur with row of 3 ventral SBTs; tibia with ventral row of 2 SBTs. LII total length 6.13 (5.49–6.07): trochanter 0.4 (0.38–0.41), femur 1.48 (1.33–1.44), patella 0.54 (0.5–0.57), tibia 1.25 (1.15–1.28), metatarsus 1.46 (1.18–1.42), tarsus 0.99 (0.91–1.02). LII/SL 2.99 (2.68–3.03).

FEMALES: 3 paratypes. Body length 2.06–2.56, scute length 1.81–2.0, greatest width of anterior scute 1.08–1.22, greatest width of opisthosoma 1.84–1.93. OC height 0.13–0.17, width 0.22–0.24. GO length 0.27–0.3, width 0.36–0.38. PCS greatest width 0.21–0.23. DCS length 0.6–0.66, greatest width 0.22–0.24. PF height 0.29–0.32. LII total length 4.4–5.19: trochanter 0.29–0.36, patella 0.47–0.51, tibia 0.91–1.05, metatarsus 0.94–1.11, tarsus 0.75–0.89. LII/SL 2.46–2.59.

**Variation.** Variation exists in the shape of the OC: individuals from Sandia Mountains have an acute pointed OC, those from Mt. Taylor are rounded, while Manzano Mountain individuals are intermediate.

**Other Material Examined.** NEW MEXICO: **Bernalillo Co.**: Sandia Mountains, C.C. Hoff, 1 female (AMNH: S354); Tejano Canyon, Sandia Mountains, 1 female (AMNH: S-275). **Cibola Co.**: Mt. Taylor (N35.2412, W107.6082), el. 3307 m, under rocks, 10 August 2013, Garrett B. Hughes, 1 male, 2 females (SDSU); near and at top of Mt. Taylor, C.C. Hoff, 1 female (AMNH); near the Lillies, Mt. Taylor (N35.2694, W107.6315), CC. Hoff, 1 male (AMNH: S-1543). **Sandoval Co.**: Sandia Mountains, C.C. Hoff, 4 males, 1 female (AMNH: S-310). **Torrance Co.**: 1 mile south Capillo Peak, NW of Manzano (N34.6883, W106.3999), C.C. Hoff, 1 female (AMNH).

**Distribution.** Known from three isolated mountain ranges in central New Mexico (Manzano Mountains, Sandia Mountains, and San Mateo Mountains).

***Sclerobunus speoventus*** **sp. nov.**

urn:lsid:zoobank.org:act:750E9704-830D-4623-AE11-FD612A200C61

Figures: **map** 3b; **penis** 8a; **habitus** 10a, S7a; **pedipalp** S8f; **leg I** S9f

**Type Material. Holotype** male from Thieves Canyon, Cave of the Winds, on moist ground and rocks, el. 2135 m, El Paso County, Colorado (N38.8728, W104.9203), collected on 12 March 2009 by D. Steinmann (DMNS 2009-44). **Paratypes:** 4 males, 1 female, 1 juvenile, same collecting data as holotype (DNMS 2009-44). Holotype male and 6 paratypes deposited at DMNS.

**Etymology.** The specific epithet is masculine and is a combination of the Latin words for cave (*speo-*) and wind (*vent-*), and refers to the type locality of Cave of the Winds.

**Diagnosis.** Differentiated from all surface *Sclerobunus* by a highly troglomorphic appearance. Distinguished from *S. steinmanni* by a relative lack of black pigment on the dorsum, slightly smaller size, the presence of a single ventral tubercle bearing spine on LI tibia and strongly curved subapical spines on the penis.

**Description.** MALE: Holotype (6 paratypes). Body length 2.34 (2.11–2.63), length of scute 1.94 (1.93–2.2), greatest width of anterior scute 1.35 (1.38–1.45), greatest width of opisthosoma 2.1 (2.1–2.3). Integument of body uniformly yellow/orange in color, anterior scute with very little, extremely faint, black pigment. Anterior margin of scute with 3–4 tubercles. OC height 0.09 (0.07–0.12), width 0.24 (0.25–0.28). OC tall, rounded, slightly wider than long, eyes connected with black pigment. Pedipalpal coxae with 2 SBTs at distal margin (lateral edge), posterior spine larger than anterior. CI with 11 spines, several large tubercles. With 2 apophyses at CII complex. GO length 0.32 (0.33–0.36), width 0.37 (0.36–0.39). Chelicerae elongate. PCS greatest width 0.25 (0.25–0.28), single dorsal SBT at distal edge, single small ventrolateral SBT at distal margin. DCS length 0.83 (0.87–0.93), width 0.28 (0.29–0.32). Pedipalps elongate. PF height 0.53 (0.49–0.61), with dorsal row of 7 small SBTs, 4 small mesodorsal spines, a row of 3 elongate medial spines, ventral row of 8 elongate spines; PT with row of 5 large medial spines, 6 lateral spines. Legs elongate. LI femur with ventral row of five SBTs, the second and third being smallest; tibia with ventral row of larger setae, one arising from a small tubercle. LII total length 10.82 (10.33–11.33): trochanter 0.42 (0.43–0.47), femur 2.5 (2.4–2.66), patella 0.8 (0.83–0.89), tibia 2.26 (2.11–2.34), metatarsus 2.59 (2.45–2.74), tarsus 2.25 (2.0–2.3). LII/SL 5.58 (4.74–5.77).

FEMALE: (N = 5; 1 paratype). Body length 2.14–2.63, scute length 1.96–2.1, greatest width of anterior scute 1.27–1.4, greatest width of opisthosoma 1.88–2.38. OC height 0.08–0.13, width 0.21–0.26. GO length 0.3–0.33, width 0.34–0.39. PCS width 0.24–0.27. DCS length 0.82–0.89, width 0.27–0.29. PF height 0.38–0.43. LII total length 9.65–10.11: trochanter 0.38–0.46, femur 2.24–2.33, patella 0.74–0.82, tibia 1.96–2.15, metatarsus 2.2–2.39, tarsus 1.93–2.08. LII/SL 4.65–5.04.

**Variation.** Variation exists in the spination of the pedipalp. The CII lobes show variation in the number of apophyses (2–3), even within an individual.

**Other Material Examined.** COLORADO: **El Paso Co.**: Cave of the Winds (N38.8728, W104.9203), Grand Concert Hall, on moist walls, 2 July 2007, S. Derkarabetian, M. Hedin, J. Reiter, 3 males (SDSU: OP1128; one used for SEM; one dissected for genitalia), 3 females (SDSU: OP1127, OP1129; one dissected for genitalia); Manitou Springs, Cave of the Winds, Manitou Grand Caverns Section, Old Water Barrel (N38.8728, W104.9203), 2 February 1984, J. W. Meacham, 1 female, 1 juvenile (CAS).

**Distribution.** Known only from type locality.

**Genetic Data.** GenBank accession numbers: KJ585343-: KJ585346, KJ585099–KJ585102, KJ585138–KJ585141, KJ585222–KJ585225, KJ585180–KJ585183, KJ585302–KJ585305, KJ585056–KJ585059, KJ585262–KJ585265.

***Sclerobunus steinmanni*** **sp. nov.**

urn:lsid:zoobank.org:act:008ED6F7-05A9-49AF-9F49-CF96B8F6E8CC

Figures: **map** 3b; **penis** 8b; **habitus** 10b; **pedipalp** S8g; **leg I** S9g

**Type Material. Holotype** male from twilight zone on wall in Mallory Cave, el. 2135 m, Boulder County, Colorado (GPS withheld), collected on 29 November 2008 by D. Steinmann (DMNS; SDSU: OP2568). OP2568 GenBank accession numbers: KJ585341, KJ585096, KJ585136, KJ585219, KJ585177, KJ585299, KJ585053. **Paratypes:** 1 male paratype, 3 female paratypes from dark zone on wall in Mallory Cave, Boulder County, Colorado, collected on 15 November 2012 by D. Steinmann (DMNS; SDSU: OP3108–OP3109). OP3108 and OP3109 GenBank accession numbers: KJ585342, KJ585097–KJ585098, KJ585137, KJ585220–KJ585221, KJ585178–KJ585179, KJ585300–KJ585301, KJ585054–KJ585055, KJ585260–KJ585261. Holotype male and paratypes deposited at DMNS.

**Etymology.** This species is named in honor of David B. Steinmann who collected all known individuals of this species as well as discovered and collected many other new populations of cave-dwelling *Sclerobunus* throughout Colorado.

**Diagnosis.** Differentiated from all surface *Sclerobunus* by a highly troglomorphic appearance. Distinguished from *S. speoventus* by the presence of some black pigment on the dorsum and legs, slightly larger size, the presence of a more than one ventral tubercle bearing setae on LI tibia, penis with normal subapical spines and dorsoventrally compressed ventral plate.

**Description.** MALE: Holotype (male paratype). Body length 2.63 (2.74), length of scute 2.38 (2.38), greatest width of anterior scute 1.74 (1.63), greatest width of opisthosoma 2.64 (2.48). Integument of body uniformly yellow/orange in color, anterior scute with faint patterned black pigment, patch of lighter pigment posterior to OC, opisthosoma with faint black pigment evenly spread. Anterior margin with 4–5 tubercles. OC height 0.09 (0.1), width 0.24 (0.26). OC tall, roughly as long as wide, with black pigment connecting eyes. Pedipalpal coxae with 1 SBT at distal margin. CI with 14 setae, some large and on tubercles. With 2 apophyses at CII complex. GO length (0.38), width (0.41). Chelicerae elongate. PCS greatest width 0.3 (0.29), 2 small dorsal STs at distal edge, 2 small ventrolateral SBTs at distal edge. DCS length 0.97 (0.93), width 0.32 (0.32). Pedipalps elongate. PF height 0.68 (0.61), with dorsal row of 6–7 small SBTs, 4 small mesodorsal spines, a row of 3–4 elongate medial spines, ventral row of 8 elongate spines; PT with row of 5 large medial spines, 5–6 lateral spines, 2 dorsal rows of small spines, several small ventral spines. Legs elongate, with faint black pigment. LI femur with ventral row of 6 SBTs, alternating between larger and smaller tubercles, the larger being more developed than in other *Sclerobunus*; tibia with ventral row of larger setae, 3 arising from tubercles, the middle being largest. LII total length 12.43 (11.84): trochanter 0.53 (0.55), femur 2.94 (2.77), patella 1.03 (0.96), tibia 2.64 (2.45), metatarsus 3.0 (2.81), tarsus 2.3 (2.3). LII/SL 5.22 (4.96). Lateral prongs of hind tarsal claw slightly reduced.

FEMALE: 3 paratypes. Body length 2.63–3.02, scute length 2.33–2.46, greatest width of anterior scute 1.58–1.59, greatest width of opisthosoma 2.58–2.69. OC height 0.12–0.13, width 0.3–0.31. GO length 0.34–0.38, width 0.42–0.44. PCS width 0.26–0.28. DCS length 0.93–0.97, width 0.29–0.31. Pedipalpal coxae with 2 distal tubercles bearing setae. PF height 0.49–0.52. LII total length 10.41–10.89: trochanter 0.54–0.55, femur 2.45–2.61, patella 0.88–0.93, tibia 2.18–2.33, metatarsus 2.4–2.5, tarsus 1.94–1.99. LII/SL 4.42–4.48.

**Distribution.** Known only from the type locality.

## Discussion

### Species Delimitation

Species delimitation is becoming increasingly objective and taxonomy more integrative, utilizing both discovery and validation approaches employing multiple lines of evidence [3]. Here, multiple lines of evidence are used to support all species of *Sclerobunus*: morphometric clustering, genetic analyses utilizing multiple nuclear loci, previously conducted mitochondrial analyses [21], [22], and general morphology. To date, only two studies have used the method of Ezard et al. [7], the original study and a second by Pearson and Ezard [70], both examining morphological differentiation in fossil foraminifera. We implemented this method on extant taxa as a means to develop *a priori* species hypotheses to be tested using hypothesis-based genetic analyses. Here, the method was used on a dataset consisting of multiple species where it successfully delimited clusters representing multiple putative species. Any clusters containing more than one species were easily differentiated using phylogenetic analyses, as either the species were not sister taxa or validation analyses confirmed genetic distinctiveness. This dataset represents a relatively simple situation in which to apply this method considering that most species of *Sclerobunus* can be distinguished by eye. However, we believe this method has great utility in modern species delimitation studies and testing its efficacy is essential, especially with regards to species complexes with little obvious morphological differentiation.

Other protocols have been developed that rely on morphological data to identify morphometric clusters that are later used as *a priori* species hypotheses. For example, Seifert et al. [71] developed a hypothesis-free protocol that uses several morphometric clustering methods, which may be used in combination with other lines of evidence, to develop species hypotheses. The “nest-centroid” clustering method was specifically developed for and demonstrated with eusocial organisms where, in the case of ants, nest samples are treated as distinct classes in multivariate statistical analyses. The application and utility of this protocol using only morphometric data was shown by the identification and description of a new cryptic ant species [72]. Given the increasing number of cryptic species being described [4], using the recently developed morphological-based methods [7], [71] as a tool for identifying putative species, particularly when used in conjunction with genetic data, is promising.

The integrative nature of this study provides support for the utility of coalescent-based methods in species delimitation, which are becoming increasingly relevant given the common discovery of cryptic species complexes [4]. Although mitochondrial genes have been used with great utility in animal systematics, many concerns have been raised about relying on mtDNA as the sole marker in phylogenetic and species delimitation analyses [73]. Similarly, some studies have demonstrated that species trees derived from multilocus nuclear datasets can provide higher levels of resolution and support relative to analyses based solely on mitochondrial genes (e.g., [74]), empirically demonstrating the need for less reliance on mitochondrial data. Under an integrative taxonomic framework, mitochondrial and nuclear datasets represent independent lines of evidence [3] and should be analyzed separately. We did not reanalyze or include previously published COI data for *Sclerobunus* for several reasons. Thorough phylogenetic analyses of COI have already been conducted, including divergence dating and *gsi* analyses, documenting deep genetic divergences between species and populations [22]. On average, animal mitochondrial genes sort four times faster than nuclear genes and tend to show more extreme levels of genetic divergence, particularly in low vagility, microhabitat specialist taxa. As such, nuclear genes provide a more conservative estimate for species delimitation decisions. We believe that for our system and dataset, including mitochondrial genes in multilocus nuclear validation analyses (BPP) would inflate genetic divergences between putative species and potentially result in a more liberal number of species delimited. For example, 37 of 41 *populations* sampled showed COI *gsi* values of 1 (monophyly), indicative of the extreme levels of mitochondrial genetic divergence seen in this genus [22]; however, at the species level, most nuclear genes still show some degree of non-monophyly (TABLE 2).

### Delimiting Cave-obligate Species

The importance of genetic data in the systematics of cave-adapted species is well documented [75], [76]; however, studies using explicit species delimitation methodology in cave taxa are scarce (e.g., [12]). All cave species in this study do not experience gene flow with other cave species given the vast distances between caves. The cave species that are recovered as sister taxa in these analyses (the *S. cavicolens* group and *S. steinmanni*/*S. speoventus*) are not likely the result of speciation after an initial cave-invasion by an ancestral species. These cave species are from distinct, unconnected cave systems and suitable habitat is not present in the intervening regions between caves. Similarly, and in addition to morphological differences, we believe this same reasoning allows us to elevate the two subspecies of *S. ungulatus* to separate species. It is probable that these cave populations are each independently derived from different, now-extinct surface populations. Even with the discovery and increasing recognition of the importance of non-cave subterranean habitats (superficial subterranean habitats) [77], post-invasion speciation is still unlikely given the vast distances between caves and likely impossible as the desert basin habitats involved do not possess superficial subterranean habitats. Gene flow with surface forms is possible in some cases, but most focal caves are surrounded by unsuitable surface habitats.

### Cave Conservation and Exploration

This study highlights the need for further conservation efforts and exploration in western North American caves. For example, *S. klomax* and *S. steinmanni* are only known from a few specimens despite multiple collecting attempts. Most other cave species of *Sclerobunus*, and many North American arachnids in general, are rare in collections. However, despite the general rarity and numerous destructive threats facing caves and the organisms inhabiting them, only a handful of taxa are listed as endangered [78]. Notably, all but one of the 12 arachnid species listed as endangered in the United States is a cave-obligate species (http://ecos.fws.gov/tess_public/SpeciesReport.do?groups=J&listingType=L&mapstatus=1).

Given the large number of caves in western North America not yet explored from a biological perspective, the potential for discovery of new cave populations of *Sclerobunus* is high. Records exist of cave-dwelling *Sclerobunus robustus* from several caves in isolated mountain ranges in Arizona [79], but these likely represent recent, local cave invasions within *S. robustus*. More importantly, several unsampled records represent potentially new species, in particular, a juvenile *Sclerobunus* collected from Crystal Falls Cave, Idaho [80]. Additional research would help clarify the interesting situation uncovered at Taos Ski Valley. To date, only 3 female specimens of *S. klomax* have been found from rock piles within a very small area (∼100 m^2^) of south-facing forested-talus slope. Further fieldwork is needed to determine if this species has a broader geographic distribution and whether it only exists in superficial subterranean habitats or can be found in any nearby caves. Taken together, the description of four new, highly troglomorphic *Sclerobunus* from Colorado and northern New Mexico, and the possibility of other new cave-adapted species and populations, portray a picture of active troglomorphic evolution in a region that is not typically considered a hotspot for cave biodiversity.

## Supporting Information

Table S1 **Taxon sampling and voucher information for all samples included in morphological (Sheet 1) and genetic analyses (Sheet 2).**
(XLSX)

Table S2 **Character key (Sheet 1), and male (Sheet 2) and female (Sheet 3) morphometric datasets.** Values highlighted in grey are missing values estimated via Multiple Imputation (see methods).
(XLSX)

Table S3 **GenBank accession numbers (Sheet 1) and genetic sampling for this study (Sheet 2).** Voucher specimens in bold are those included in the 4-taxon panel (see methods).
(XLSX)

File S1 **Results of the traditional principal components analyses.**
(PDF)

File S2 **Additional molecular methodology including RNA extraction and sequencing, primer design, PCR conditions, and Sanger sequencing.** Supplemental table including locus and primer information.
(PDF)

File S3 **Results of the Grummer et al.** [**46**] **analyses on the** ***S. glorietus*** **complex, including Bayes Factor values and likelihood scores.** Additionally, the *BEAST species tree and BPP analyses in which each population of the *S. glorietus* complex are treated as an individual species are shown.
(PDF)

File S4 **Google Earth kmz file including georeferenced locations for all recent fieldwork including collecting localities included in this study, museum records (AMNH, CAS, DMNS), and all publication records.** To maintain protection of certain caves, GPS coordinates for cave localities are not exact.
(KML)

File S5 **Statistical results of the Ezard et al.** [**7**] **morphometric analyses for male and female datasets, including retained components, group assignments (clusters), outlier assignments (significant outliers highlighted), BIC model choice (model chosen is highlighted), squared eigenvalues (retained components are highlighted), and component loadings.** The female spreadsheet also includes resulting plots for cluster and species assignments.
(XLSX)

File S6 **Maximum likelihood gene trees estimated using RAxML. Asterisks correspond to nodes recovered with a bootstrap value >80.**
(PDF)

File S7 **Comparative male habitus and ocularium morphology. In D and E, arrows indicate sexually dimorphic structures.**
(PDF)

File S8 **Comparative pedipalpal morphology.**
(PDF)

File S9 **Comparative leg I morphology. “Morph” numbers correspond to those in Table S2.**
(PDF)
