# Correction: Integrative Taxonomy and Species Delimitation in Harvestmen: A Revision of the Western North American Genus Sclerobunus (Opiliones: Laniatores: Travunioidea)

**DOI:** 10.1371/journal.pone.0112001

**Published:** 2014-10-22

**Authors:** The PLOS ONE Staff

There is an error in the funding statement. Please refer to the complete funding statement here:

This research was supported by funds from the Cave Conservancy Foundation (Doctoral Fellowship in Karst Studies), Society of Systematic Biologists (Graduate Student Research Award), and the American Arachnological Society (Vincent Roth Fund for Systematics Research) awarded to S Derkarabetian. This research was also supported by an NSF grant awarded to M Hedin (DEB 1354558). The funders had no role in study design, data collection and analysis, decision to publish, or preparation of the manuscript.
