# Supplementary material for: Integrative Taxonomy and Species Delimitation in Harvestmen: A Revision of the Western North American Genus Sclerobunus (Opiliones: Laniatores: Travunioidea)
Source: PLoS One. 2014 Aug 21;9(8):e104982. doi: 10.1371/journal.pone.0104982 (PMC4140732; doi:10.1371/journal.pone.0104982)

STANDARD PRINCIPAL COMPONENTS RESULTS

We used SPSS v. 20 (IBM Corp.) to conduct principal components (PCA) and discriminant function analyses (DFA) on datasets in which missing values were estimated using Multiple Imputation. PCA analyses were done on both correlation and covariance matrices for separate male and female datasets. To increase sample size for cave populations, we conducted a “cave only” PCA analyses on all samples from cave populations regardless of sex. DFA analyses were conducted on the entire (both male and female) dataset.

PCA - Male Specimens

The correlation analysis, recovering 3 principal components explaining 92.8% of the variation (PC1: 59.5%, PC2: 25.2%, PC3: 8.1%), is shown in FIGURE 1. In 3-dimensional morphospace, all species are recovered in distinct clusters, with the exception of a population of *Sclerobunus robustus* from Bradford Canyon clustering with the *S. glorietus* complex. In addition, *S. glorietus* is recovered as two distinct clusters, which correspond to the northern (*S. glorietus*) and southern split (*S. skywalkeri*). The covariance analysis (FIGURE 1) recovered 2 principal components accounting for 97.5% of the variation (PC1: 81.4%, PC2: 16.1%). Similarly, the covariance analysis recovers distinct northern and southern *S. glorietus* clusters, with the Bradford Canyon *S. robustus* individuals clustering with the southern *S. r. glorietus* (*S. skywalkeri*).

PCA - Female Specimens

The correlation and covariance analyses (FIGURE 2) each had two principal components explaining 88% (PC1: 66.5%, PC2: 21.6%) and 97.1% (PC1: 71.9%, PC2: 25.2%) of the variation, respectively, and show similar clustering as in the male analyses. The 3 *S. klomax* specimens cluster near *S. jemez* and *S. cavicolens* samples in the correlation analysis and overlap with *S. cavicolens* in the covariance analysis.

PCA - Cave Species

The correlation and covariance analyses (FIGURE 3) each had two principal components explaining 81.3% (PC1: 67.1%, PC2: 14.2) and 95.4% (PC1: 80.2%, PC2: 15.2%) of the variation, respectively. In the correlation analysis, all species form exclusive clusters, while in the covariance analysis *S. klomax*, slightly overlaps with both *S. cavicolens* and *S. jemez*.

DFA - All Species

Using separate-group covariance matrices for classification, all individuals are predicted to occur within *a priori* species assignments. When using a within-group covariance matrix, several specimens are not predicted to occur within the *a priori* species groupings (FIGURE 4). The two specimens from Bradford Canyon (*S. robustus*) were predicted to occur in *S. skywalkeri*, which is not surprising as they are the smallest population of *S. robustus* known. The two female specimens from Mt. Taylor (*S. skywalkeri*) were predicted to occur with *S. glorietus*. Again, this is not surprising as females are generally smaller than males, and *S. glorietus* as a species is smaller than *S. skywalkeri*. Additionally, a single *S. idahoensis* (a male from Goose Creek, MT) was predicted within *S. nondimorphicus*. This population has the largest body size of all *S. idahoensis* sampled.

FIGURE 1. PCA of male specimens

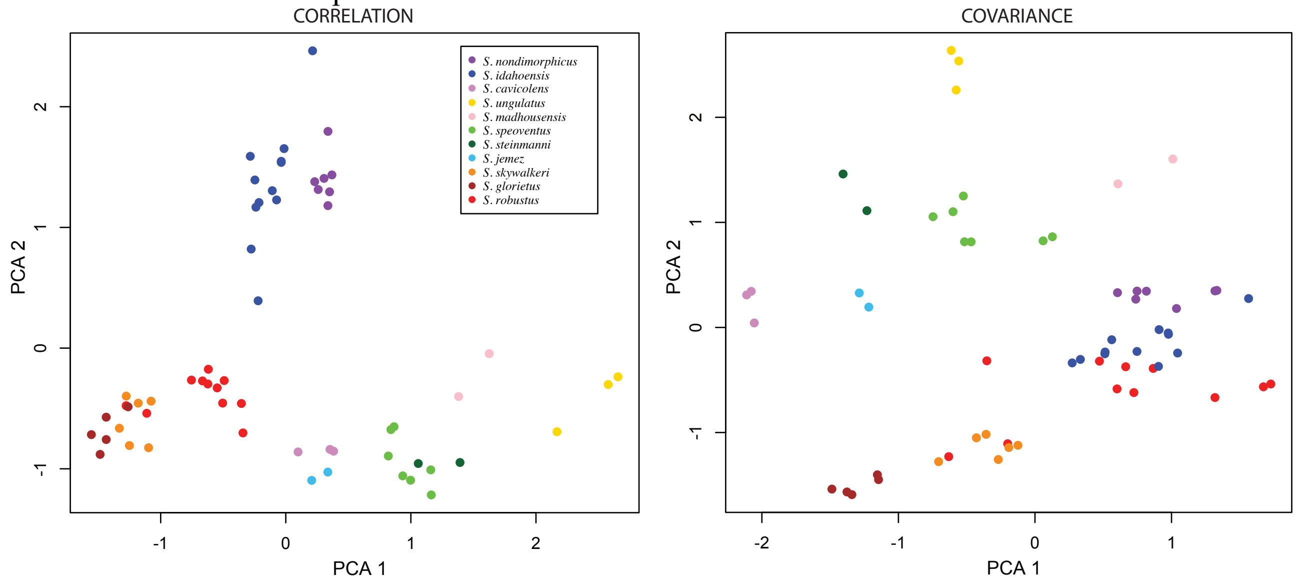

FIGURE 2. PCA of female specimens

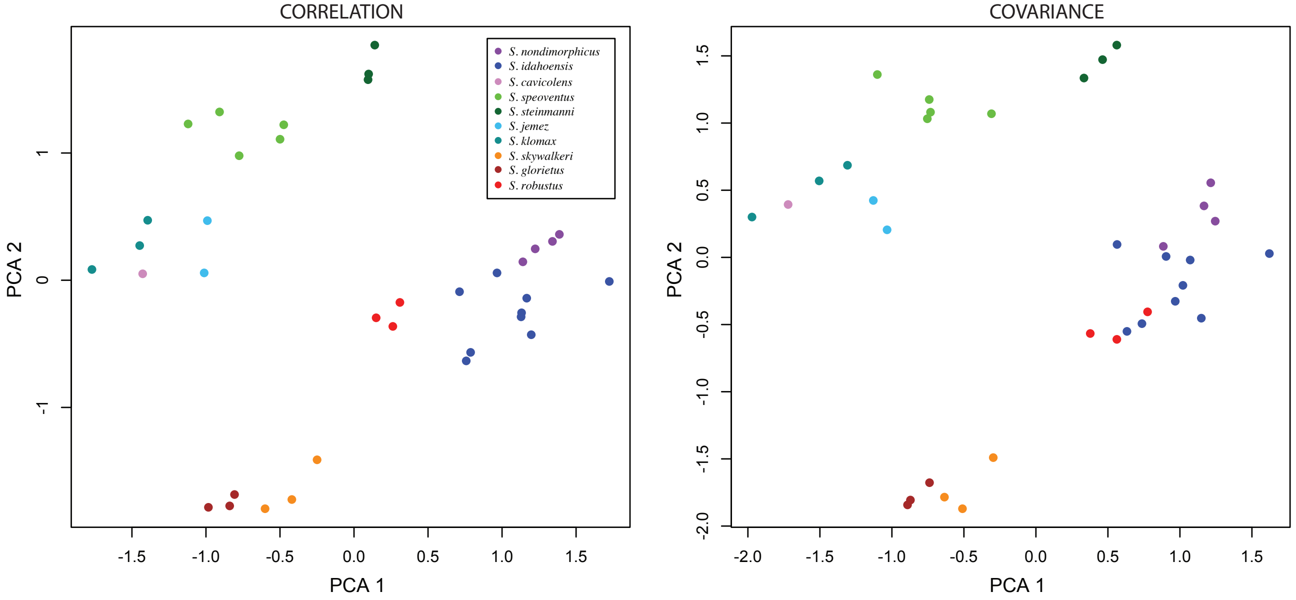

FIGURE 3. PCA of all cave specimens

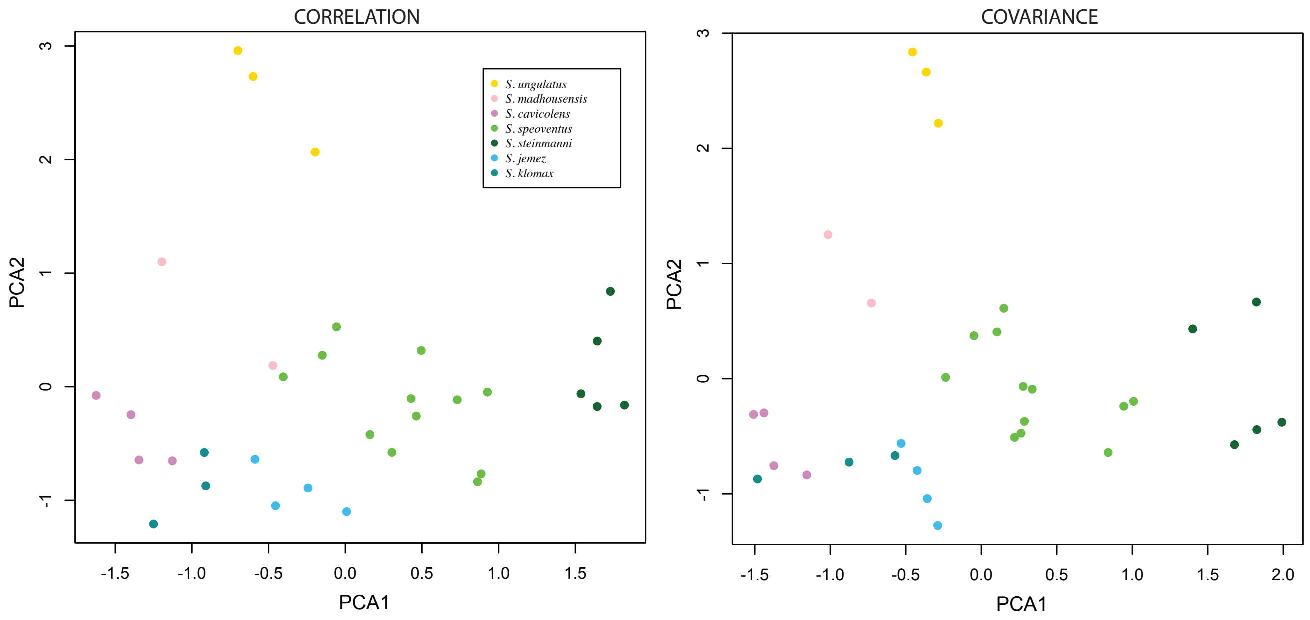

FIGURE 4. DFA of all specimens

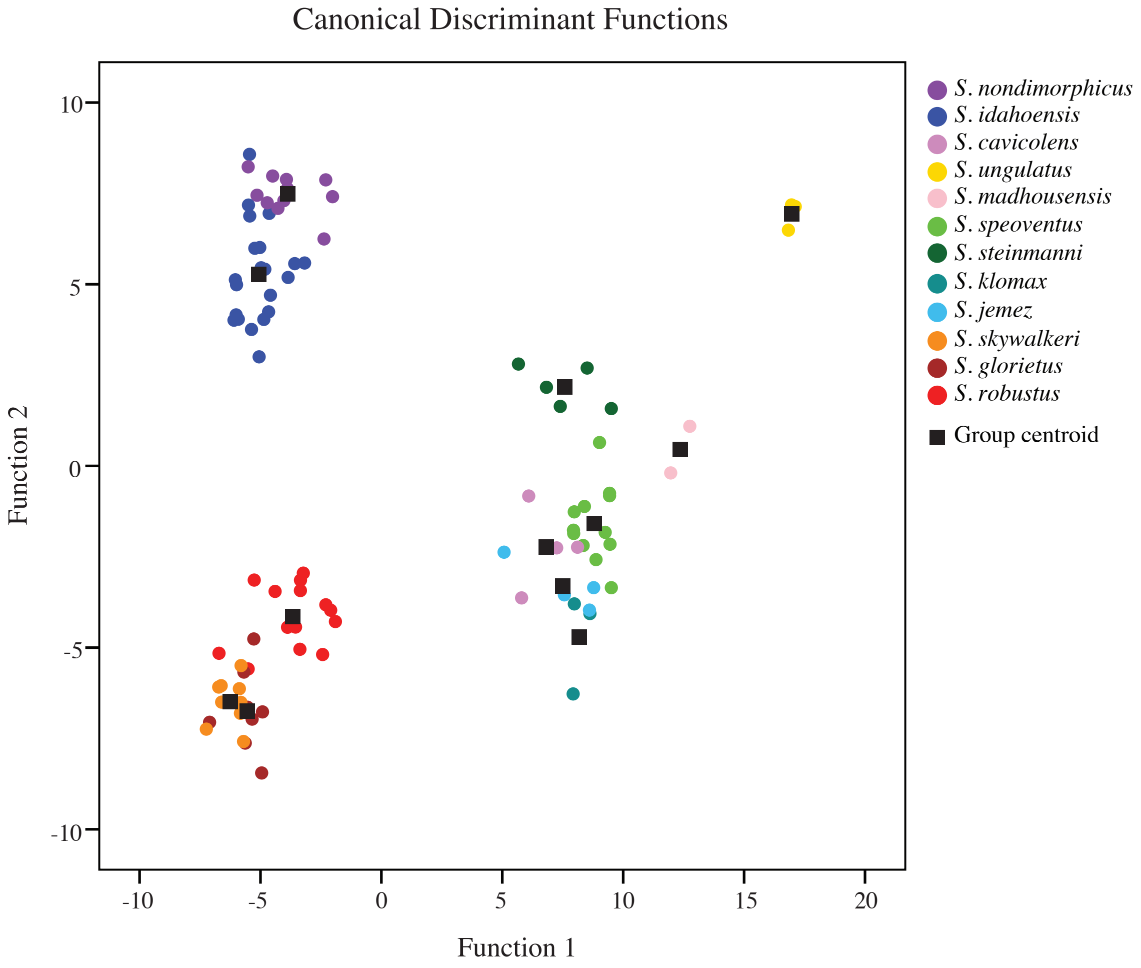

Supplement: File S1 — Results of the traditional principal components analyses. (PDF) [file pone.0104982.s004.pdf]
