# Supplementary material for: Integrative Taxonomy and Species Delimitation in Harvestmen: A Revision of the Western North American Genus Sclerobunus (Opiliones: Laniatores: Travunioidea)
Source: PLoS One. 2014 Aug 21;9(8):e104982. doi: 10.1371/journal.pone.0104982 (PMC4140732; doi:10.1371/journal.pone.0104982)
Supplement: File S2 — Additional molecular methodology including RNA extraction and sequencing, primer design, PCR conditions, and Sanger sequencing. Supplemental table including locus and primer information. (PDF) [file pone.0104982.s005.pdf]

Recent studies have shown the potential for high throughput data in shallow level phylogenetics [1-3] and the utility of transcriptomic data in phylogenetics [e.g., 4,5] including studies in harvestmen [6]. Illumina transcriptome data were already available for *Sclerobunus nondimorphicus* and the outgroup genus *Theromaster* [6]. To supplement these data, total RNA was extracted from a population of *S. robustus* from the Huachuca Mountains, Arizona. Prior to RNA extraction, the midgut was dissected out while immersed in RNAlater to reduce potential microbial contamination. Total RNA was extracted from whole specimens (midgut removed) using TRIzol (Invitrogen, Carlsbad, CA) and the RNeasy MiniElute Cleanup Kit (Qiagen, Valencia, CA). RNA samples were sent to the HudsonAlpha Institute for Biotechnology (www.hudsonalpha.org) where normalized libraries were prepared and 50 base pair paired-end reads were sequenced using Illumina HiSeq technology. The raw read data were quality filtered using Trim Galore! (http://www.bioinformatics.babraham.ac.uk/projects/trim_galore/) and Prinseq [7] and assembled using default parameters in Trinity [8] on an Intel XEON server with 100 GB of SDRAM. Raw reads have been deposited in the Sequence Read Archive [BioProject ID PRJNA254507: accessions SRS654678, SRS654733, SRS654734]. Assembled transcriptomes were searched locally against known single copy, single exon *Ixodes* proteins (http://www.vectorbase.org) to identify orthologous genes shared in all three harvestmen transcriptomes in Geneious Pro 6 (http://www.geneious.com). Via comparison of these orthologs, specifically between the two congeneric *Sclerobunus* transcriptomes, hundreds of rapidly evolving exon and UTR gene regions were identified, and PCR primers were designed targeting 8 of these gene regions for analyses (see table below).

Genomic DNA was extracted from specimens (2-3 legs) using the Qiagen Dneasy Kit (Qiagen, Valencia, CA), per manufacturer's protocol. All PCR reactions contained 0.8 µl of genomic DNA mixed into a cocktail containing 2.5 µl of each primer (10.0 µl), 2.5 µl 10X PCR buffer, 0.5 µl 10mM dNTP mixture (0.2 mM of each dNTP), 0.75 µl 50 mM Magnesium Chloride, 0.1 µl of Platinum *Taq* DNA polymerase (Invitrogen), and 15.3 µl of tissue culture water bringing the total reaction to 25 µl. All 8 nuclear gene regions were amplified using a *single* set of PCR cycle conditions: 94ºC for 3 min, (94ºC for 60 sec, 62ºC for 75 sec, 72ºC for 90 sec) repeated 10 times, followed by (94ºC for 15 sec, 57ºC for 75 sec, 72ºC for 90 sec) repeated 30 times, then 72ºC for 5 min. PCR products from successful experiments were plate purified (Millipore) and sent to Macrogen USA for direct Sanger sequencing.

1. Lemmon AR, Lemmon EM (2012) High-throughput identification of informative nuclear loci for shallow-scale phylogenetics and phylogeography. Syst Biol 61: 745-761.
2. McCormack JE, Hird SM, Zellmer AJ, Carstens BC, Brumfield RT (2011) Applications of next-generation sequencing to phylogeography and phylogenetics. Mol Phylogenet Evol 66: 526-538.
3. McCormack JE, Maley JM, Hird SM, Derryberry EP, Graves GR, Brumfield RT (2012) Next-generation sequencing reveals phylogeographic structure and a species tree for recent bird divergences. Mol Phylogenet Evol 62: 397-406.
4. Kocot KM, Cannon JT, Todt C, Citarella MR, Kohn AB, et al. (2011) Phylogenomics reveals deep molluscan relationships. Nature 477: 452-456.
5. Hartmann S, Helm C, Nickel B, Meyer M, Struck TH, et al. (2012). Exploiting gene families for phylogenomic analysis of myzostomid transcriptome data. PLOS One 7(1): e29843.
6. Hedin M, Starrett J, Akhter S, Schönhofer AL, Shultz, JW (2012) Phylogenomic resolution of Paleozoic divergences in harvestmen (Arachnida, Opiliones) via analysis of next-generation transcriptome data. PLOS One 7(8): e42888.
7. Schmieder R, Edwards R (2011) Quality control and preprocessing of metagenomic datasets. Bioinformatics 27: 863-864.
8. Grabherr MG, Haas BJ, Yassour M, Levin JZ, Thompson DA, et al. (2011) Full-length transcriptome assembly from RNA-Seq data without a reference genome. Nature Biotechnol 29: 644-652.

Locus Name	Abbreviation	Region	Primer Name	Direction	Primer Sequence	ISCW vectorbase
Tetratricopeptide repeat protein	TPR	Exon	ScleroA1	Forward	CAACGAGAACCAATGCACATTGAAGGC	ISCW000183
			ScleroA2	Reverse	TTGATGTCTACCAATCTATGGAATGCG	
Conserved hypothetical protein	CHP1	Exon	ScleroA5	Forward	TGAATCGTCAAATGATTATTGGACCAGC	ISCW000678
			ScleroA6	Reverse	TTGTTGACTATCACGACCAGCTATYACC	
Conserved hypothetical protein	CHP2	Exon	ScleroB5	Forward	CGCTGGACGAATTGGAGTCATCGGAG	ISCW000678
			ScleroB6	Reverse	CATGCTTCTCGAATGCTTGATATATGC	
Neuromusculin	nrm	3' UTR	ScleroD7	Forward	CGATTTGTACGATGATGTGAAGACTGGC	ISCW006547
			ScleroD8	Reverse	GCATGTTACGCGAGAAGTGATAACAGTC	
1,4-dihydroxyl-2-napthoate octaprenyltransferase	DNO	Exon	ScleroE7	Forward	GCTGCCTTAGCSTACAAGTGTGCCCAAG	ISCW005714
			ScleroE8	Reverse	GTCTCGGAACCCTTTCTCCAAGTGGAAG	
Ski-interacting protein	SKI	Exon	ScleroF10	Forward	AACGTCTAGCAGCCGACGGCAGAGG	ISCW021146
			ScleroF11	Reverse	TYGGACGCTTGCTCGCCTTCTTAGC	
Double-stranded RNA-specific editase B2	ADARB2	Exon	ScleroG7	Forward	AATCGYAAGCGCCGTAGTGGAATGGATGG	ISCW018470
			ScleroG8	Reverse	GCAATCGTTAAGGGCAGCACCATTCATAC	
Phosphoglycerate mutase	PGM	3' UTR	ScleroH8	Forward	TCACAATGCMGCGCTGTGTGACGTGGC	ISCW020443
			ScleroH9	Reverse	ATACCTAACAATAGRAACTAGCCTGG	
