# Supplementary material for: Integrative Taxonomy and Species Delimitation in Harvestmen: A Revision of the Western North American Genus Sclerobunus (Opiliones: Laniatores: Travunioidea)
Source: PLoS One. 2014 Aug 21;9(8):e104982. doi: 10.1371/journal.pone.0104982 (PMC4140732; doi:10.1371/journal.pone.0104982)
Supplement: File S3 — Results of the Grummer et al. [46] analyses on the S. glorietus complex, including Bayes Factor values and likelihood scores. Additionally, the *BEAST species tree and BPP analyses in which each population of the S. glorietus complex are treated as an individual species are shown. (PDF) [file pone.0104982.s006.pdf]

Results of the species limits testing using the Grummer et al. [1] method. GlorCan = Glorieta Canyon. TSV surf = Taos Ski Valley surface population. TSVtrog = *S. klomax*. glorS = *S. skywalkeri*.

**Species Limits Testing**	**-lnL**	**BF**
GlorCan + TSVsurf; TSVtrog; glorS	-19481.2477	2.3138
GlorCan + glorS; TSVtrog + TSVsurf	-19480.0908	0
GlorCan + glorS; TSVtrog; TSVsurf	-19481.2280	2.2744
GlorCan + TSVtrog + TSVsurf; glorS	-19481.753	3.3244

Species tree and BPP results from analyses in which all populations of the *glorietus* complex are each treated as putative species.

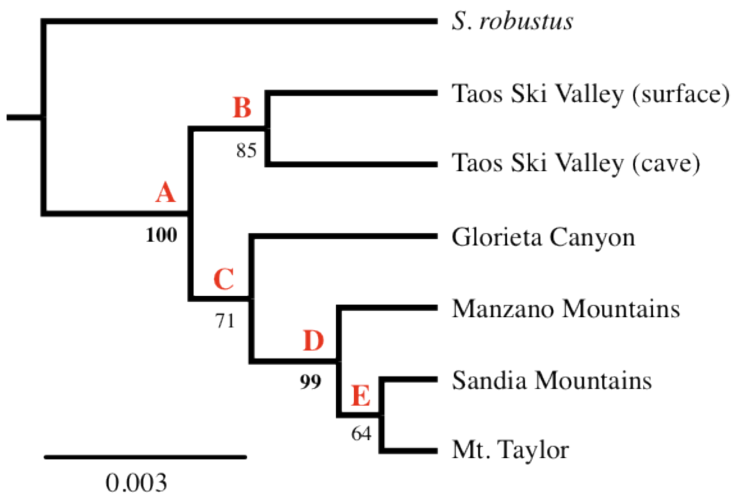

BP&P	A	B	C	D	E
$\theta \sim \Gamma(2,10), \tau \sim \Gamma(2,10)$	1	1	0.99	0.86	0.32
$\theta \sim \Gamma(2,10), \tau \sim \Gamma(2,1000)$	1	1	0.99	0.85	0.32
$\theta \sim \Gamma(2,1000), \tau \sim \Gamma(2,10)$	1	1	1	1	0.99
$\theta \sim \Gamma(2,1000), \tau \sim \Gamma(2,1000)$	1	1	1	1	0.99

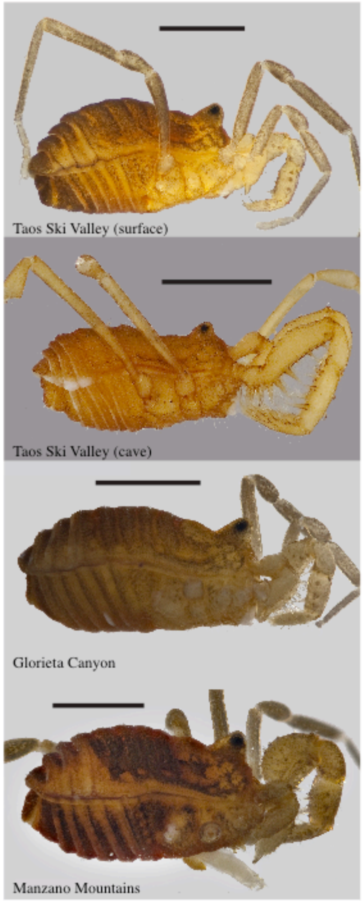

1. Grummer JA, Bryson RW, Reeder TW (2014) Species delimitation using Bayes factors: simulations and application to the *Sceloporus scalaris* species group (Squamata: Phrynosomatidae). Syst Biol 63: 119-133.
