# Supplementary material for: Integrative Taxonomy and Species Delimitation in Harvestmen: A Revision of the Western North American Genus Sclerobunus (Opiliones: Laniatores: Travunioidea)
Source: PLoS One. 2014 Aug 21;9(8):e104982. doi: 10.1371/journal.pone.0104982 (PMC4140732; doi:10.1371/journal.pone.0104982)
Supplement: File S6 — Maximum likelihood gene trees estimated using RAxML. Asterisks correspond to nodes recovered with a bootstrap value >80. (PDF) [file pone.0104982.s009.pdf]

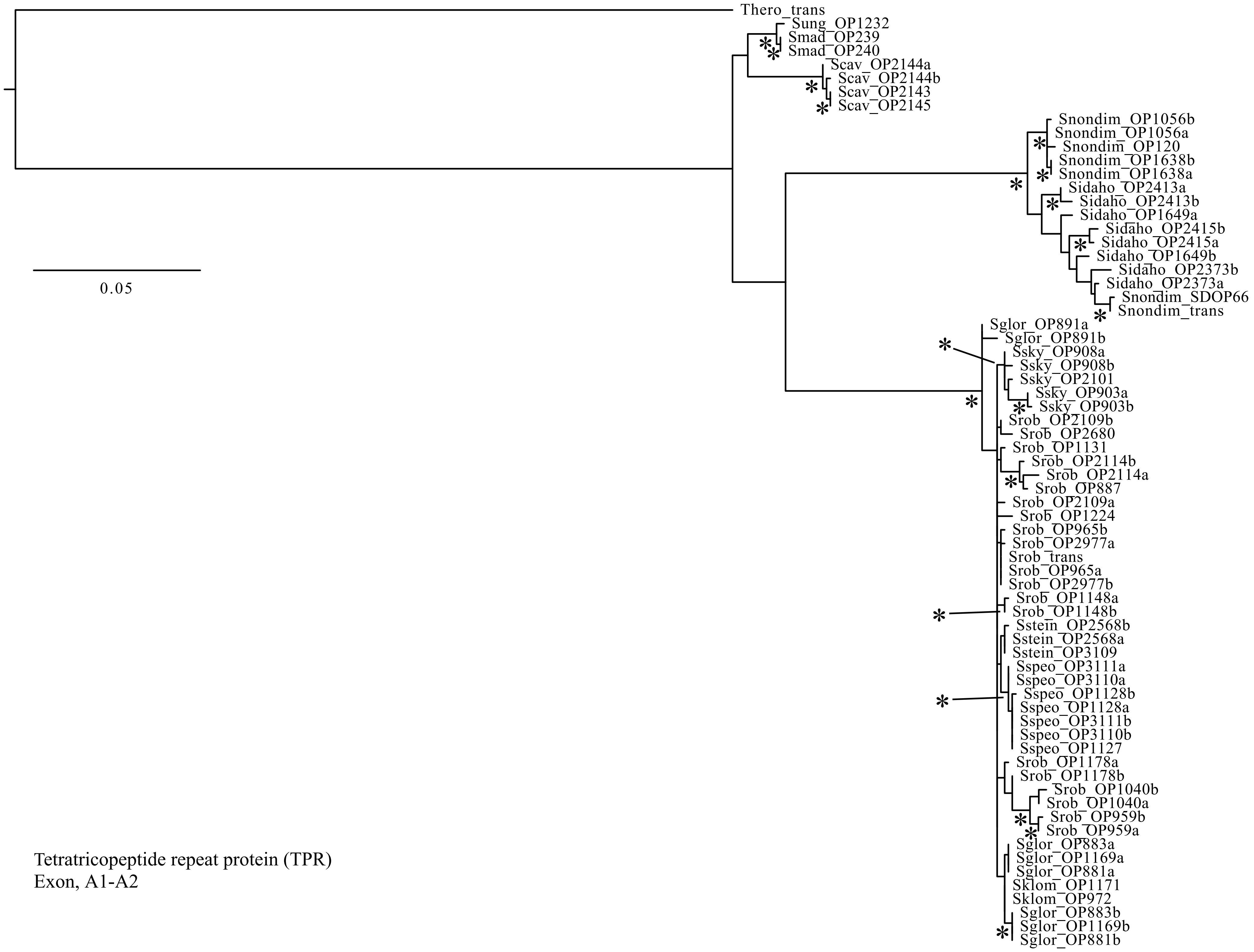

Tetratricopeptide repeat protein (TPR)
Exon, A1-A2

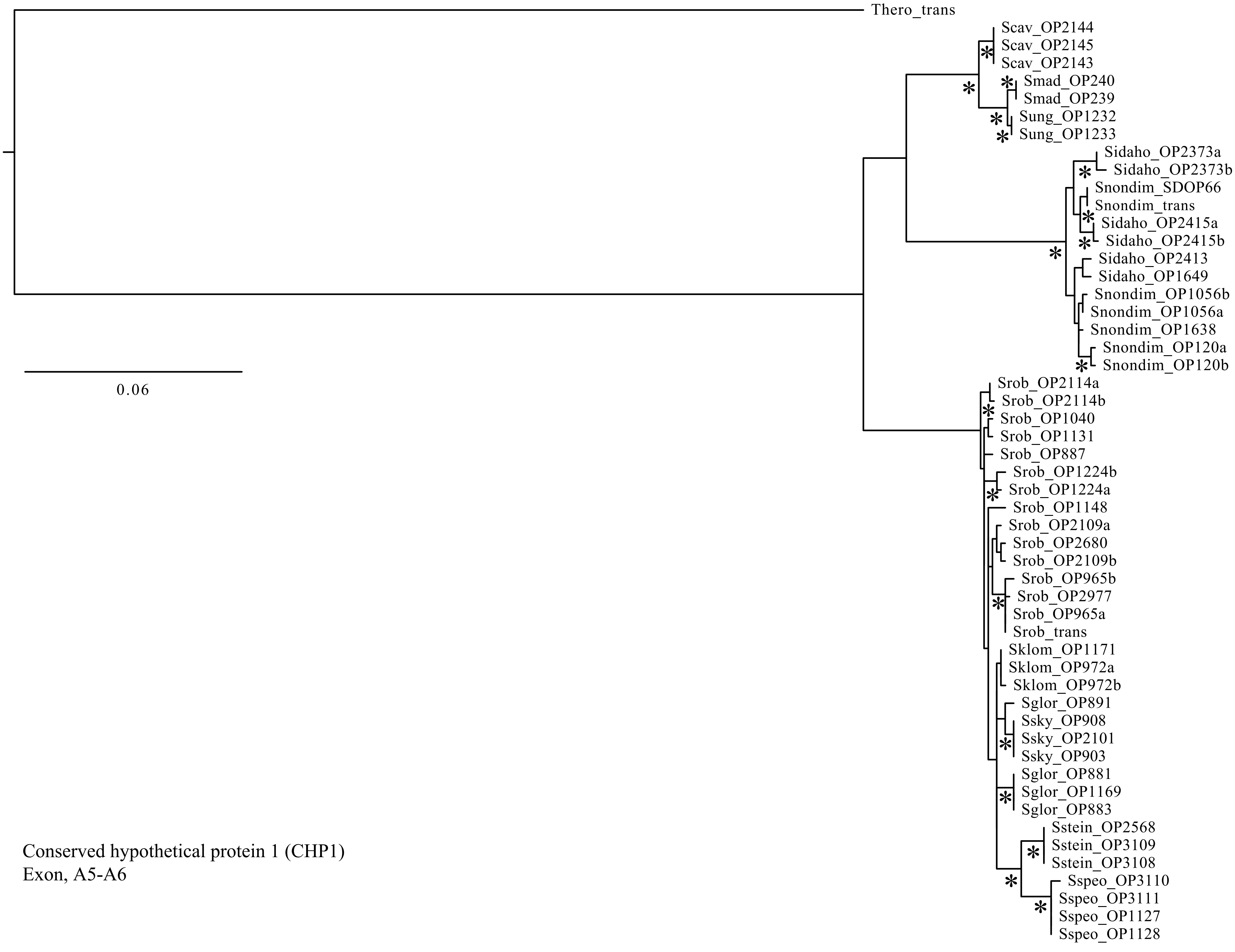

Conserved hypothetical protein 1 (CHP1)
Exon, A5-A6

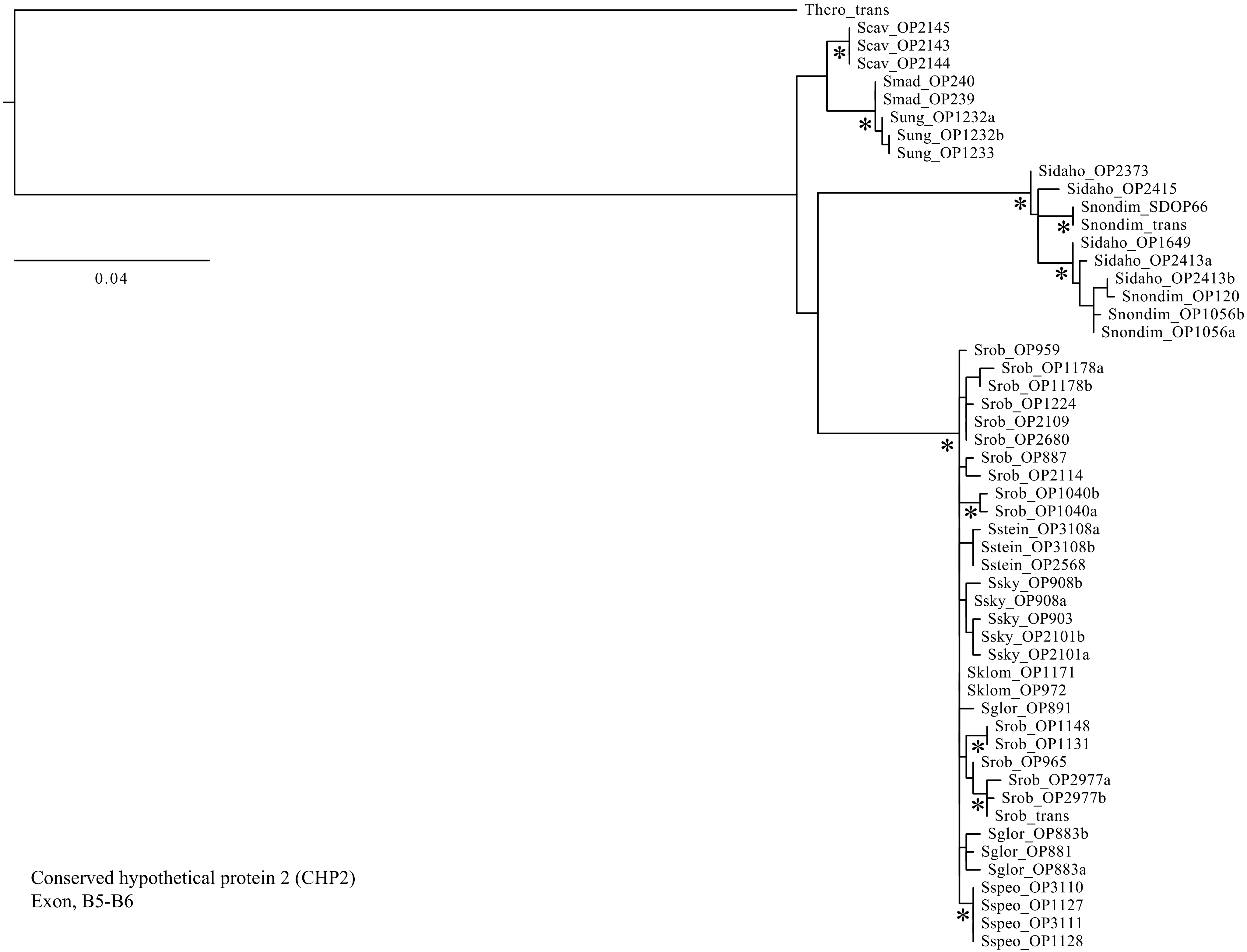

Conserved hypothetical protein 2 (CHP2)
Exon, B5-B6

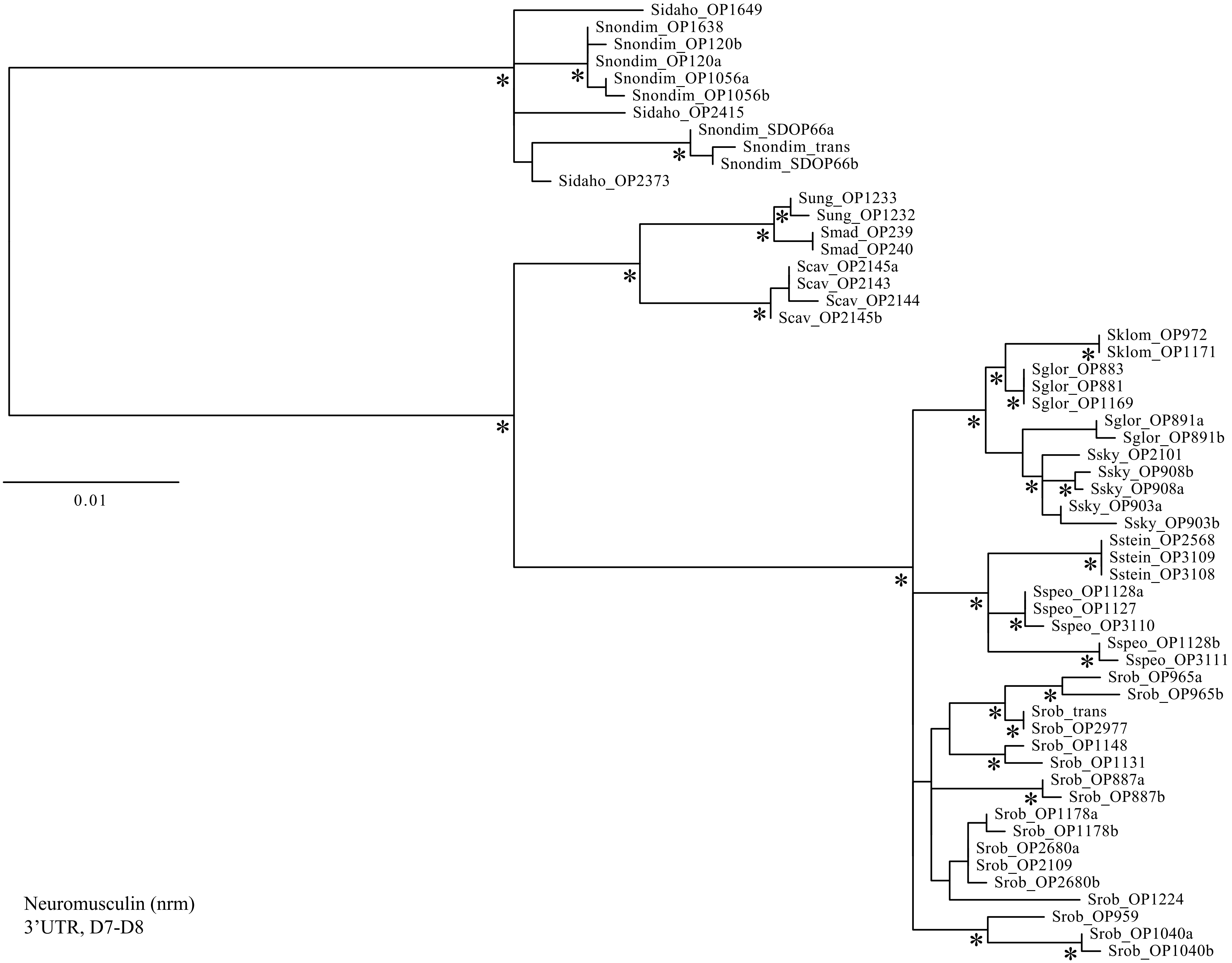
Sidaho_OP1649
Snondim_OP1638
Snondim_OP120b
Snondim_OP120a
Snondim_OP1056a
Snondim_OP1056b
Sidaho_OP2415
Snondim_SDOP66a
Snondim_trans
Snondim_SDOP66b
Sidaho_OP2373
Sung_OP1233
Sung_OP1232
Smad_OP239
Smad_OP240
Scav_OP2145a
Scav_OP2143
Scav_OP2144
Scav_OP2145b
Sklom_OP972
Sklom_OP1171
Sglor_OP883
Sglor_OP881
Sglor_OP1169
Sglor_OP891a
Sglor_OP891b
Ssky_OP2101
Ssky_OP908b
Ssky_OP908a
Ssky_OP903a
Ssky_OP903b
Sstein_OP2568
Sstein_OP3109
Sstein_OP3108
Sspeo_OP1128a
Sspeo_OP1127
Sspeo_OP3110
Sspeo_OP1128b
Sspeo_OP3111
Srob_OP965a
Srob_OP965b
Srob_trans
Srob_OP2977
Srob_OP1148
Srob_OP1131
Srob_OP887a
Srob_OP887b
Srob_OP1178a
Srob_OP1178b
Srob_OP2680a
Srob_OP2109
Srob_OP2680b
Srob_OP1224
Srob_OP959
Srob_OP1040a
Srob_OP1040b
0.01
Neuromusculin (nrm)
3'UTR, D7-D8

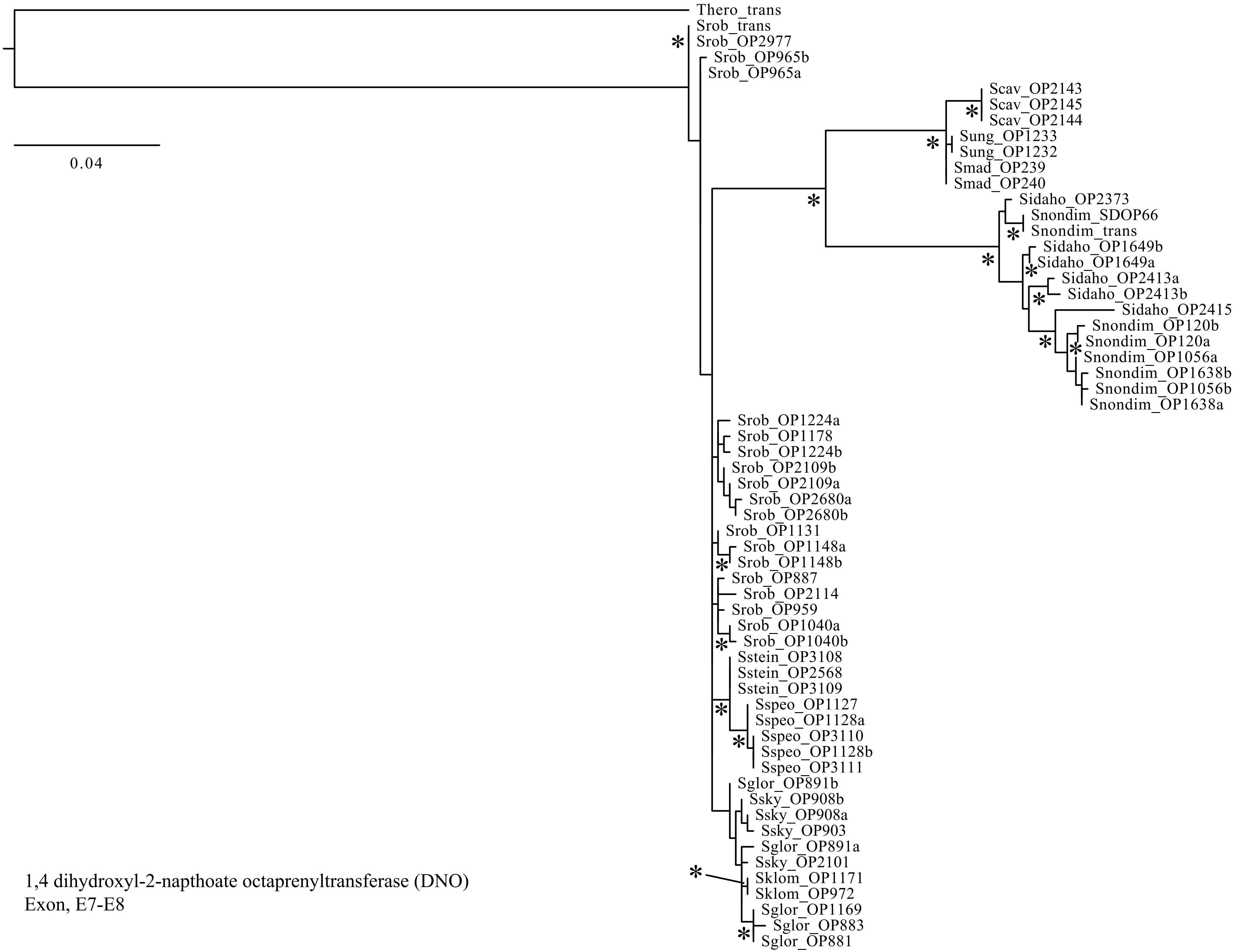

1,4 dihydroxyl-2-napthoate octaprenyltransferase (DNO)
Exon, E7-E8

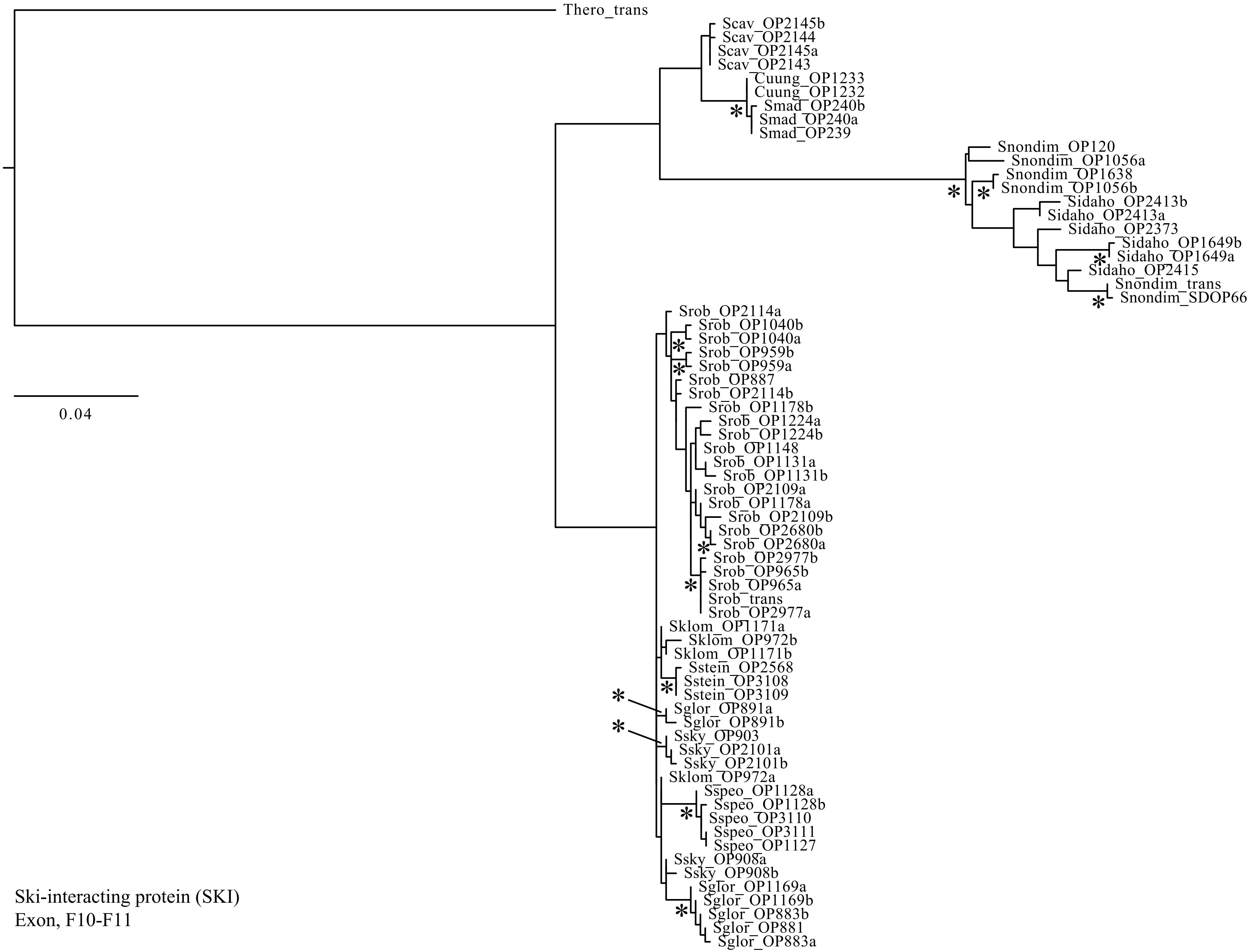

Ski-interacting protein (SKI)
Exon, F10-F11

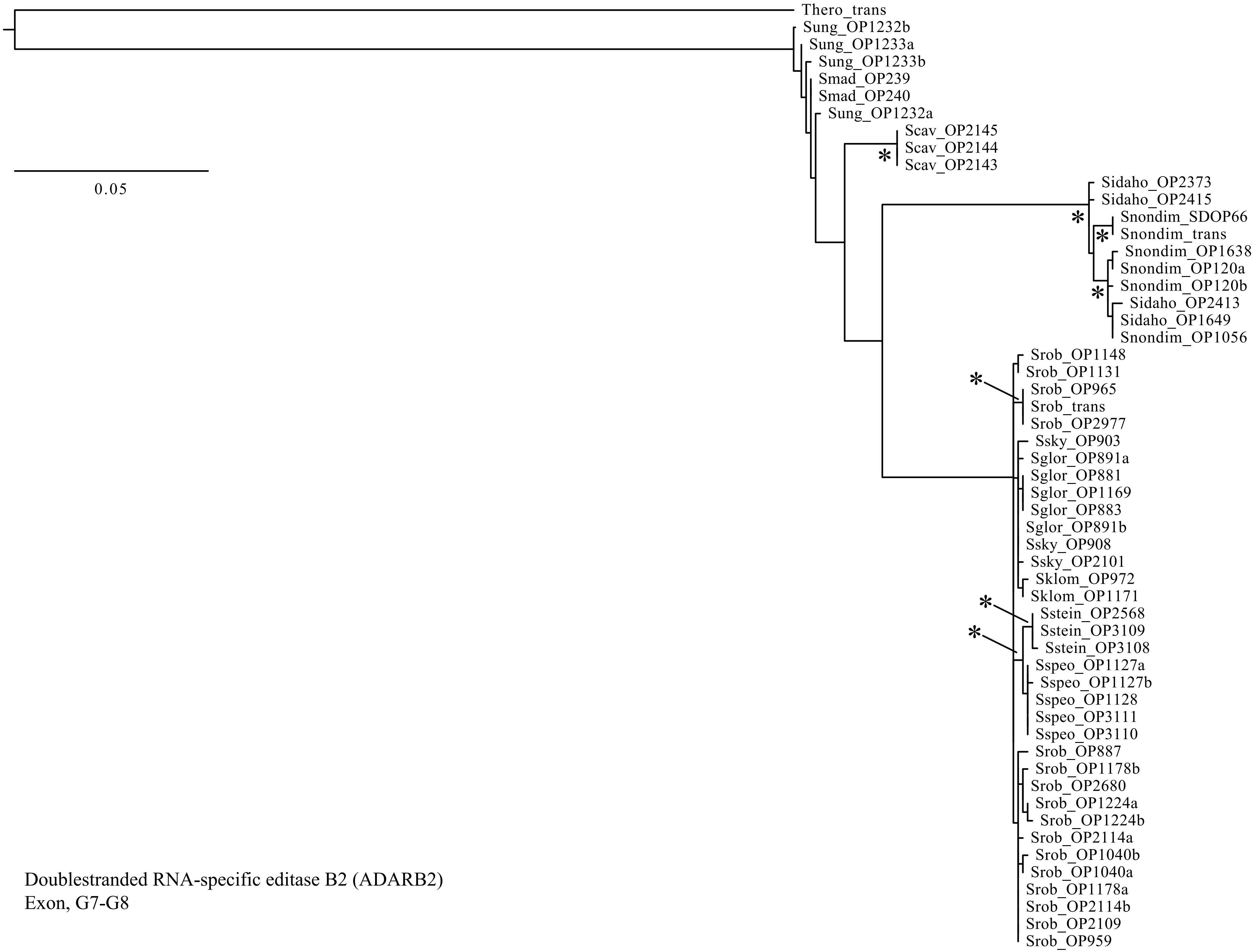

Doublestranded RNA-specific editase B2 (ADARB2)
Exon, G7-G8

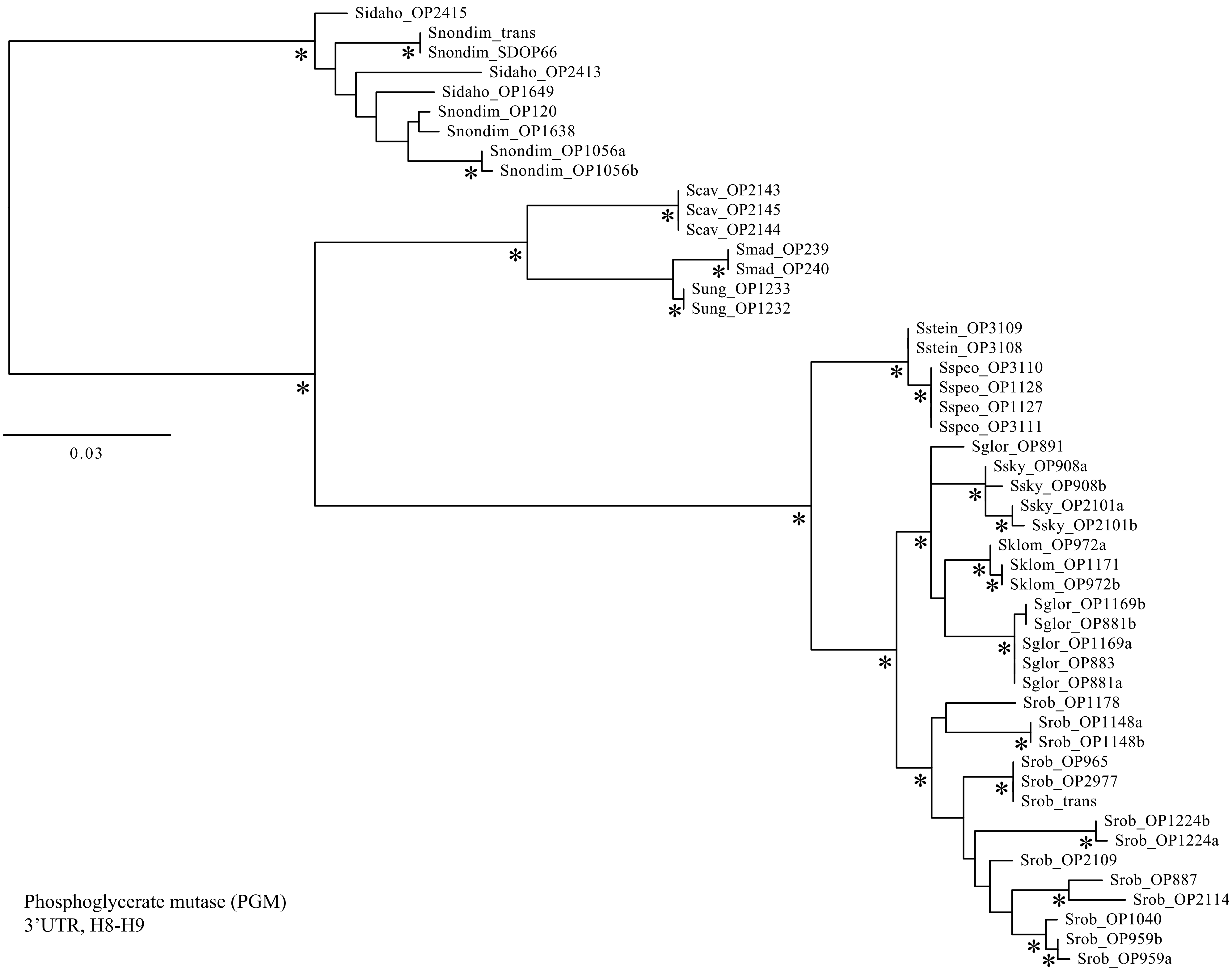

Phosphoglycerate mutase (PGM)
3'UTR, H8-H9
