# Supplementary material for: Integrative Taxonomy and Species Delimitation in Harvestmen: A Revision of the Western North American Genus Sclerobunus (Opiliones: Laniatores: Travunioidea)
Source: PLoS One. 2014 Aug 21;9(8):e104982. doi: 10.1371/journal.pone.0104982 (PMC4140732; doi:10.1371/journal.pone.0104982)
Supplement: File S7 — Comparative male habitus and ocularium morphology. In D and E, arrows indicate sexually dimorphic structures. (PDF) [file pone.0104982.s010.pdf]

Comparative male habitus morphology

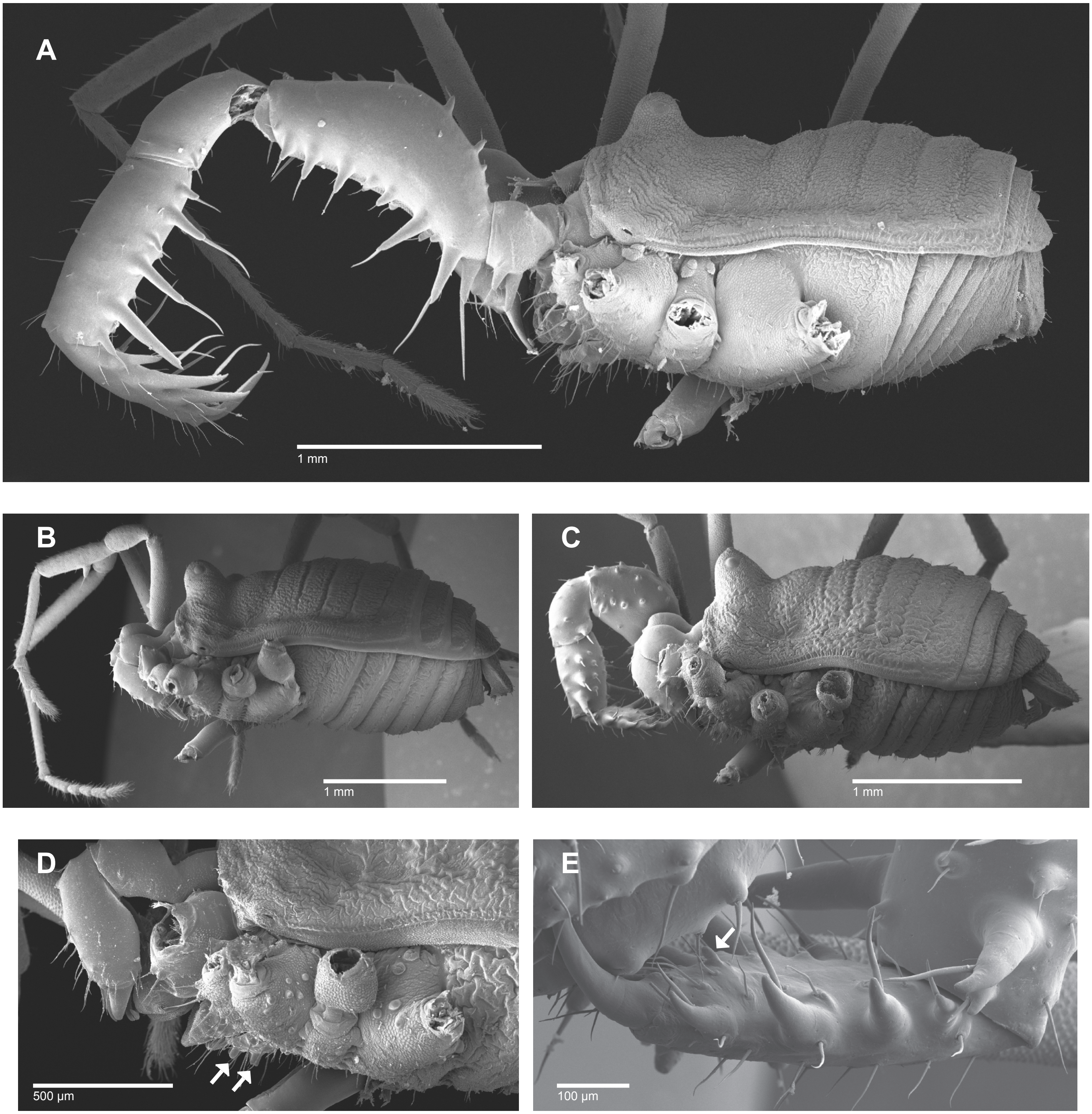

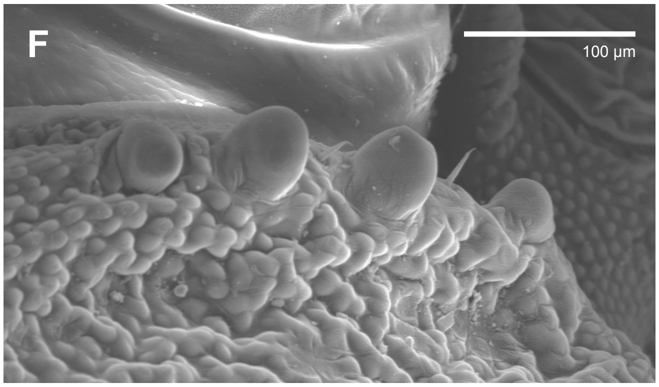

A *S. speoventus* (Cave of the Winds)

B *S. robustus* (Haviland Lake)

C *S. skywalkeri* (Manzano Mtns.)

D *S. glorietus* (Taos Ski Valley), CII lobe apophyses, 2 pairs

E *S. robustus* (North of Chama), right palpal tarsus apophysis, retrolateral

F *S. robustus* (Deer Creek Trail), right shoulder tubercles, dorsal
