# Supplementary material for: Integrative Taxonomy and Species Delimitation in Harvestmen: A Revision of the Western North American Genus Sclerobunus (Opiliones: Laniatores: Travunioidea)
Source: PLoS One. 2014 Aug 21;9(8):e104982. doi: 10.1371/journal.pone.0104982 (PMC4140732; doi:10.1371/journal.pone.0104982)
Supplement: File S8 — Comparative pedipalpal morphology. (PDF) [file pone.0104982.s011.pdf]

Comparative pedipalpal morphology

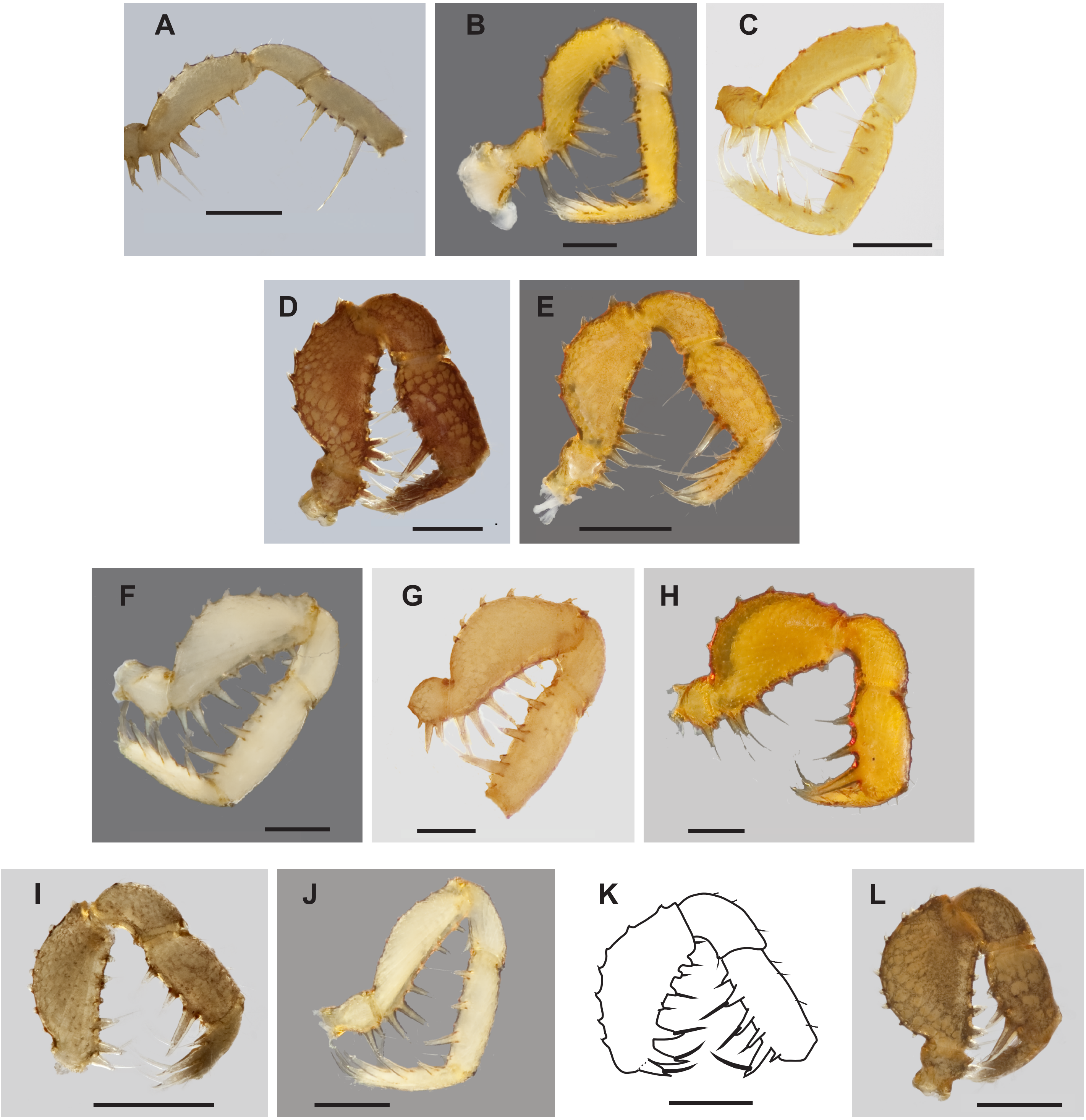

A *S. cavicolens* (Lewis and Clark Caverns)
B *S. ungulatus* (Model Cave)
C *S. madhousensis* (North Madhouse Cave)
D *S. nondimorphicus* (Iron Creek)
E *S. idahoensis* (Hobo Cedar Grove)
F *S. speoventus* (Cave of the Winds)
G *S. steinmanni*, holotype (Mallory Cave)
H *S. robustus* (Apex Valley)
I *S. glorietus* (Glorieta Canyon)
J *S. klomax*, paratype female (Taos Ski Valley)
K *S. jemez*, holotype (Terrero Cave)
L *S. skywalkeri*, paratype (Manzano Mtns.)

Images reflected horizontally: A,D,K
Scale bars = 0.5 mm
