# Supplementary material for: Integrative Taxonomy and Species Delimitation in Harvestmen: A Revision of the Western North American Genus Sclerobunus (Opiliones: Laniatores: Travunioidea)
Source: PLoS One. 2014 Aug 21;9(8):e104982. doi: 10.1371/journal.pone.0104982 (PMC4140732; doi:10.1371/journal.pone.0104982)
Supplement: File S9 — Comparative leg I morphology. “Morph” numbers correspond to those in Table S2. (PDF) [file pone.0104982.s012.pdf]

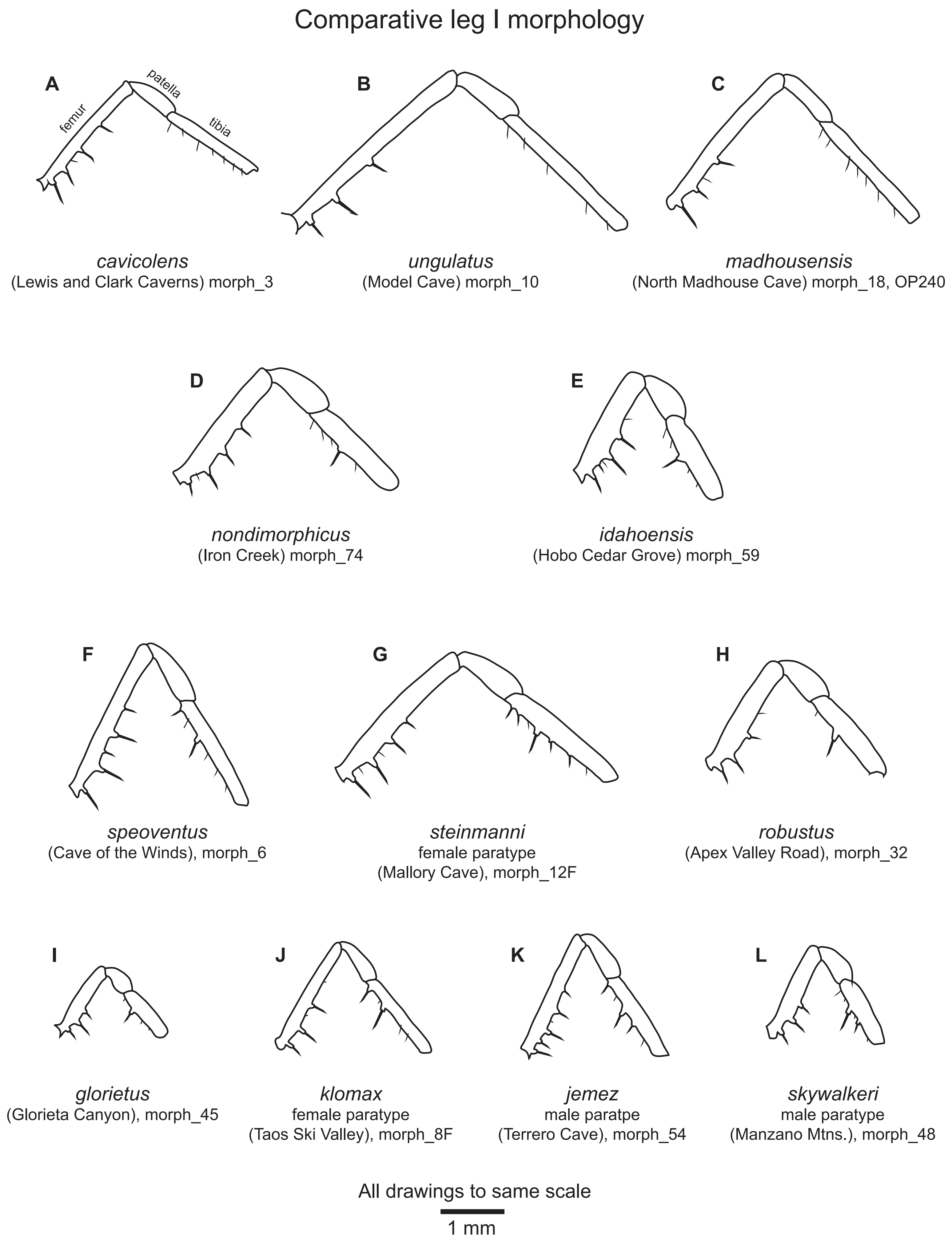
Comparative leg I morphology
A
femur
patella
tibia
cavicolens
(Lewis and Clark Caverns) morph_3
B
ungulatus
(Model Cave) morph_10
C
madhousensis
(North Madhouse Cave) morph_18, OP240
D
nondimorphicus
(Iron Creek) morph_74
E
idahoensis
(Hobo Cedar Grove) morph_59
F
speoventus
(Cave of the Winds), morph_6
G
steinmanni
female paratype
(Mallory Cave), morph_12F
H
robustus
(Apex Valley Road), morph_32
I
glorietus
(Glorieta Canyon), morph_45
J
klomax
female paratype
(Taos Ski Valley), morph_8F
K
jemez
male paratpe
(Terrero Cave), morph_54
L
skywalkeri
male paratype
(Manzano Mtns.), morph_48
All drawings to same scale
1 mm
